# Formation of Ruthenium Carbenes by *gem*-Hydrogen Transfer to Internal Alkynes: Implications for Alkyne *trans*-Hydrogenation

**DOI:** 10.1002/anie.201506075

**Published:** 2015-08-31

**Authors:** Markus Leutzsch, Larry M Wolf, Puneet Gupta, Michael Fuchs, Walter Thiel, Christophe Farès, Alois Fürstner

**Affiliations:** Max-Planck-Institut für Kohlenforschung 45470 Mülheim/Ruhr (Germany) E-mail: fuerstner@kofo.mpg.de

**Keywords:** alkynes, carbenes, density functional calculations, hydrogenation, NMR spectroscopy, ruthenium

## Abstract

Insights into the mechanism of the unusual *trans*-hydrogenation of internal alkynes catalyzed by {Cp*Ru} complexes were gained by para-hydrogen (p-H_2_) induced polarization (PHIP) transfer NMR spectroscopy. It was found that the productive *trans*-reduction competes with a pathway in which both H atoms of H_2_ are delivered to a single alkyne C atom of the substrate while the second alkyne C atom is converted into a metal carbene. This “*geminal* hydrogenation” mode seems unprecedented; it was independently confirmed by the isolation and structural characterization of a ruthenium carbene complex stabilized by secondary inter-ligand interactions. A detailed DFT study shows that the *trans* alkene and the carbene complex originate from a common metallacyclopropene intermediate. Furthermore, the computational analysis and the PHIP NMR data concur in that the metal carbene is the major gateway to olefin isomerization and over-reduction, which frequently interfere with regular alkyne *trans*-hydrogenation.

Although it has been known for some time that the cationic complex [Cp*Ru(MeCN)_3_]PF_6_ (**1**; Cp*=pentamethylcyclopentadienyl) catalyzes the stereochemically uncommon *trans*-hydrosilylation of internal alkynes,[1–3] it was recognized only recently that the scope of the underlying reactivity mode is actually much larger. Specifically, this and related catalysts are also able to effect highly selective *trans*-hydroboration,[4] *trans*-hydrogermylation,[3, 5] *trans*-hydrostannation,[3, 6, 7] and even *trans*-hydrogenation reactions.[8, 9] In many cases, the use of neutral precatalysts, such as [Cp*RuCl(cod)] (**2**; cod=cycloocta-1,5-diene) or [{Cp*RuCl}_4_] (**3**), in lieu of the cationic species **1** allows significantly better regioselectivities to be imposed on such *trans*-additions when working with non-symmetric alkyne substrates.[3]

Whereas the preparative significance of these transformations stems from their stereochemical complementarity to the existing arsenal,[10] little is known about their mechanism. They formally violate the reigning paradigm that metal-catalyzed additions to π-bonds proceed through suprafacial delivery of H_2_, H–BR_2_, or H–ER_3_ (E=Si, Ge, Sn).[11] The unorthodox stereochemical course may arise from the intervention of ruthenacyclopropenes (η^2^-vinyl complexes)[12] as suggested by an in-depth computational study for the hydrosilylation manifold.[13] Although it seems reasonable to assume that the other transformations mentioned above follow similar pathways,[14] secured information is largely missing, and alternative mechanisms cannot be ruled out. Specifically, scenarios involving more than one metal center have been proposed in early studies.[15, 16]

Catalytic *trans*-hydrogenation constitutes a favorable alternative to dissolving metal reductions of alkynes[17] and as such holds the promise of being highly enabling. Yet, its full potential can only be harnessed if side reactions, such as olefin isomerization and over-reduction, are suppressed.[8, 9] To this end, a better understanding of the entire reaction manifold—including the competing pathways—will be necessary. Therefore, we embarked on a mechanistic study that commenced with NMR investigations using para-hydrogen (p-H_2_) induced polarization (PHIP) transfer. This hyperpolarization method has the potential of selectively enhancing the signals originating from the reacting H_2_ by up to four orders of magnitude over the conventional Boltzmann-governed NMR polarization.[18] This massive effect allows the fate of the hydrogen nuclei to be surveyed and (fleeting)[19] intermediates to be detected, characterized, and tracked—provided that the H atoms are transferred in a pairwise fashion; moreover, they have to be magnetically inequivalent and mutually coupled.[20, 21] In the present study, the spectra were recorded at 11.7 T (500 MHz (^1^H)) using zero-field (ALTADENA)[18b] and high-field (PASADENA)[18c] experiments with a double-quantum OPSY filter introduced in all pulse sequences.[22]

As a first foray, we showed that the *trans*-alkenes formed upon semi-hydrogenation of a set of representative alkyne substrates with enriched p-H_2_ (ca. 5 bar) in the presence of **1** or **2** (ca. 5 mol %) in a high-pressure NMR tube invariably exhibit PHIP-enhanced olefinic ^1^H signals; a representative example is shown in Figure 1. This result confirms that both H atoms of a single H_2_ molecule are transferred pairwise to the substrate and thus corroborates a conclusion reached for a different ruthenium source in an earlier spectroscopic study.[15] In stark contrast to this previous report, however, which had failed to detect any intermediates, additional PHIP-enhanced signals were observed in the aliphatic region. In all cases investigated, these resonances showed large negative scalar coupling constants in the range of −15 to −17 Hz, suggesting the presence of geminally coupled diastereotopic methylene protons; this assignment was confirmed by ^1^H-OPSY-COSY spectra. Interestingly, alkyne **4 a** (R=H) led to two sets of such signals (in addition to the signals of the *E*-alkene product **5 a**; Figure 1), the different multiplicities of which are consistent with the presence of two regioisomers **6 a** and **7 a**.[23] As a flanking OR group (R=H, Me) seemed to increase the lifetime of such intermediates, we went on to study sterically encumbered substrates of type **8** with two potentially stabilizing substituents (Figure 2). As expected, these alkynes gave rise to a single intermediate each, which was stable enough for full characterization by conventional NMR spectroscopy. The recorded data left no doubt that the resulting complexes **9** are ruthenium carbenes that must have been formed by geminal hydrogenation of the triple bond, as the PHIP NMR data confirmed that both transferred H atoms definitely arose from the very same H_2_ molecule.

**Figure 1:**
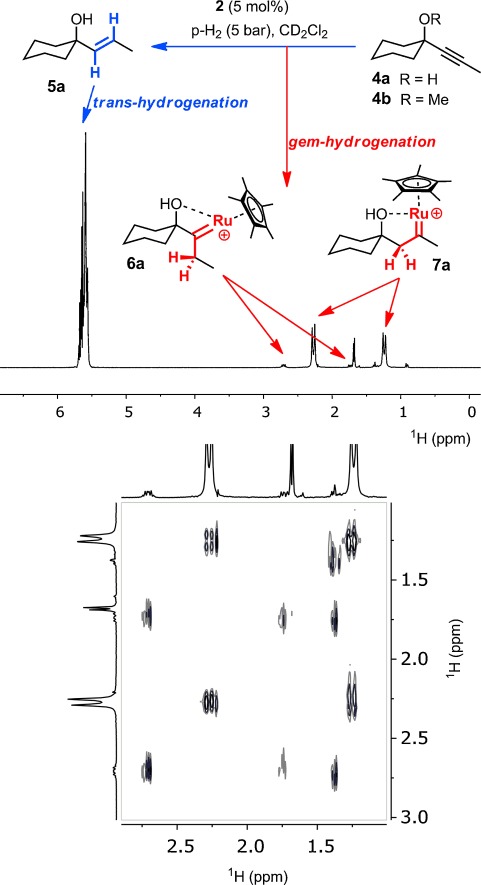
Top: ^1^H-OPSY NMR spectrum taken during the catalytic hydrogenation of 4 a (R=H) with p-H_2_. Bottom: aliphatic region of a ^1^H-OPSY-COSY spectrum, confirming the spin system of the carbene isomers 6 a/7 a causing the PHIP-enhanced signals. For the assignment of all visible peaks, see the Supporting Information.

**Figure 2:**
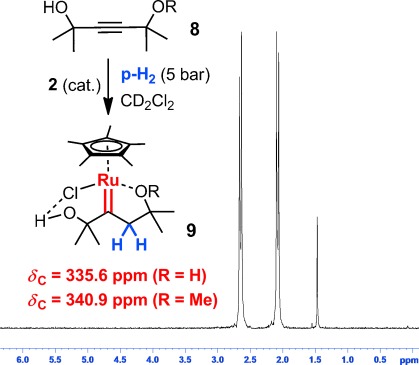
Aliphatic region of the ^1^H-OPSY NMR spectrum showing the *gem*-coupled protons that must have been delivered pairwise during the hydrogenation of 8 a (R=H) with p-H_2_ in the presence of catalytic amounts of complex 2; characteristic ^13^C NMR data of the resulting carbenes 9 a (R=H) and 9 b (R=Me).

In a formal sense, a carbene such as **9** can be thought of as being derived from an alkyne substrate that has reacted as a species with 1,2-dicarbene character: One of the vicinal carbene sites gets trapped by the ruthenium fragment, whereas the other one oxidatively inserts into the H–H bond of the reagent (Scheme 1). The resulting *geminal* delivery of H_2_ to a single carbon center is highly unorthodox in the realm of organic chemistry,[24] even though it mimics nothing but the prototype reactivity mode of transition metals vis-à-vis hydrogen gas.[25]

**Scheme 1:**
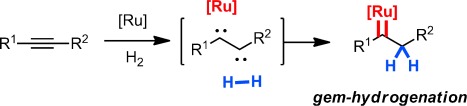
Formalism underlying the *gem*-hydrogenation of an alkyne.

The long lifetimes of the intermediates observed by NMR spectroscopy suggested that even their isolation in pure form might be possible. Our attempts were met with success for the slightly modified substrate **8 b** (R=Me) bearing one OH and one OMe substituent. The structure of the derived carbene **9 b** (*δ*_C_=340.9 ppm) in the solid state confirms the conclusions drawn from the NMR data (Figure 3). The carbene nature is evident from the almost perfectly planar coordination geometry about the C1 center and the short Ru1–C1 bond (1.883(2) Å), which is only slightly longer than the Ru=CHPh bond in prototype Grubbs carbenes (1.79–1.85 Å).[26, 27] Secondary interactions confine the carbene site of **9 b** within a cyclic array, which precludes an optimal orbital overlap with either vicinal C–H bond and hence renders a conceivable 1,2-H shift unfavorable. The stabilizing peripheral contacts consist of a donor–acceptor bond between the ether oxygen atom O2 and the Ru center (2.230 Å) and a hydrogen bond between the OH and the chloride ligand (3.19 Å).[28] It is interesting to note that the carbene site in **9 b** resides next to the alcohol function and distal to the ether; this observation is in line with our previous conclusion that inter-ligand hydrogen bonding exerts a massive directing effect on *trans*-additions to non-symmetric alkynes as long as catalysts comprising polarized Ru–Cl bonds are used.[3]

**Figure 3:**
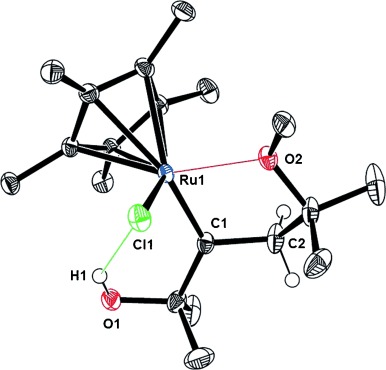
Structure of the ruthenium carbene 9 b (R=Me) in the solid state.

The remarkable stabilities of **6** and **9** raise the question as to whether these species play any role in the *trans*-hydrogenation or whether they are merely thermodynamic sinks off the catalytic cycle. The latter possibility was ruled out by additional PHIP NMR experiments with the metastable carbene **7 b** (Figure 4). Specifically, cross peaks in a 2D ^1^H-OPSY-EXSY experiment[29] definitely linked this hyperpolarized species to *E*-alkene **5 b**;[30] importantly though, the same spectrum also unmistakably connected **7 b** to the isomerized product **10** as well as to alkane **11**, which is formed by over-reduction.[31]

**Figure 4:**
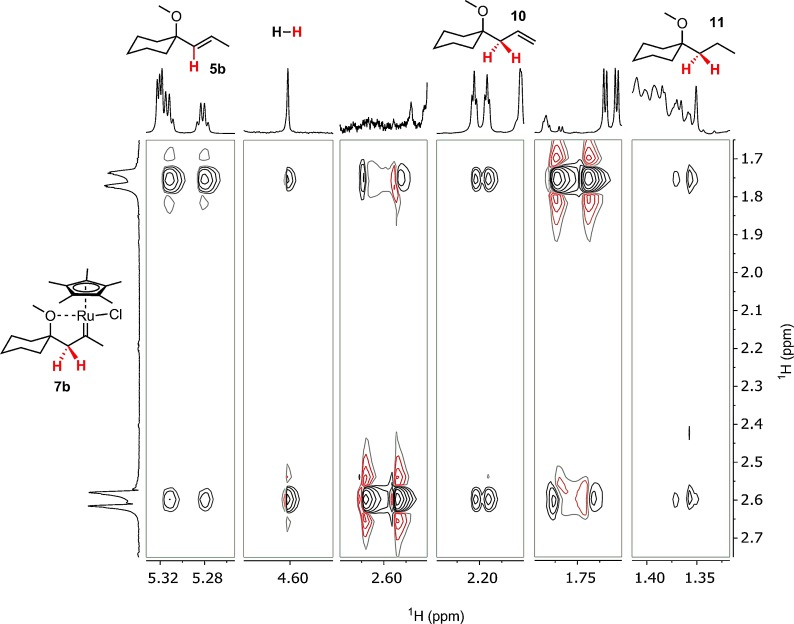
Strips of the 2D ^1^H-OPSY-EXSY spectrum (CD_2_Cl_2_, 298 K) showing characteristic cross peaks that link the carbene 7 b with products 5 b, 10, and 11.

To better understand the role and fate of the carbene intermediates, a detailed computational study was carried out at the M06/def2-TZVP/SMD(CH_2_Cl_2_)//M06/def2-TZVP level of theory (for computational details, see the Supporting Information). Complex **9 b** served as a calibration point, the molecular structure and reactivity of which were well reproduced.[32] After this validation, a more detailed mechanistic analysis was carried out for the general 2-butyne substrate (Scheme 2). Starting from **2** (=**A0**), a heteroleptic complex **A1** is formed upon substrate binding and coordination of H_2_ through its σ-bond. This notion is in excellent accord with our previous experimental finding that the σ-hydrogen complex [Cp*Ru(H_2_)(cod)]OTf (OTf=trifluoromethanesulfonate) is indeed a competent *trans*-selective hydrogenation catalyst.[8] Subsequent activation of the H–H bond in **A1** affords a short-lived dihydride species **A2**, which transfers one of the H atoms to the alkyne when passing over the low-lying transition state **TS_A2–A3_** (Δ*G*^≠^= +2.1 kcal mol^−1^).[33]

**Scheme 2:**
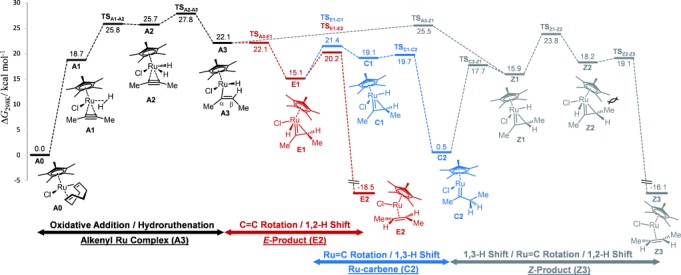
Free energy profile for the hydrogenation of 2-butyne with complex 2 (=A0) at 298 K; computed structures of pertinent intermediates (for the full set, see the Supporting Information).

At the stage of the resulting η^1^-vinyl complex **A3**, the reaction pathway bifurcates for a first time: An almost barrier-free rotation around the C_α_–C_β_ axis (**TS_A3–E1_**) leads to ruthenacyclopropene **E1**, which evolves, via the stereochemistry-determining **TS_E1–E2_**, into the final *E*-2-butene (**E2**); product formation is strongly exergonic (by 33.6 kcal mol^−1^) and therefore likely irreversible. Rotation around the C_α_–C_β_ axis in **A3** in the opposite direction opens the channel leading to the undesired *Z*-2-butene (**Z3**). This rotation, however, is not barrier-free (Δ*G*^≠^=+3.4 kcal mol^−1^). Therefore, we can safely deduce that very little of the *Z*-alkene will form, which is in accord with the generally excellent *E*/*Z* ratios observed in Ru-catalyzed *trans*-hydrogenations of unstrained alkyne substrates.[8, 9]

Of particular relevance is the finding that the ruthenacyclopropene **E1** cannot only evolve into the desired product **E2** by the pathway outlined above, but can also be converted into a true carbene **C2** upon rotation about the Ru–C_α_ bond. The decisive transition state **TS_E1–C1_** is only 1.2 kcal mol^−1^ higher in energy than **TS_E1–E2_**, which leads to **E2**. Carbene formation is exergonic by 14.6 kcal mol^−1^; considering that a flanking OR substituent may provide an estimated additional stabilization in the order of 7 kcal mol^−1^, it is very plausible that carbenes such as **9** gain lifetimes long enough for spectroscopic observation or even isolation.[32] Moreover, the computations nicely concur with the conclusions drawn from the PHIP NMR data that the geminal protons of the CH_2_ group flanking the carbene center in **C2** indeed derive from a single H_2_ molecule.

Most relevant for the understanding of the product spectrum is the finding that the 16-electron carbene species **C2** is capable of binding and activating a second molecule of H_2_ (Schemes 3 and 4).[34] The effective barriers associated with the evolution of the resulting hydrogen adducts are invariably lower (by ca. 3 kcal mol^−1^) than that of the unimolecular reverse reaction of **C2** to **E1** (Δ*G*^≠^= +20.9 kcal mol^−1^), thus implying that this reaction channel constitutes a kinetically favorable outlet for the carbene once formed.

**Scheme 3:**
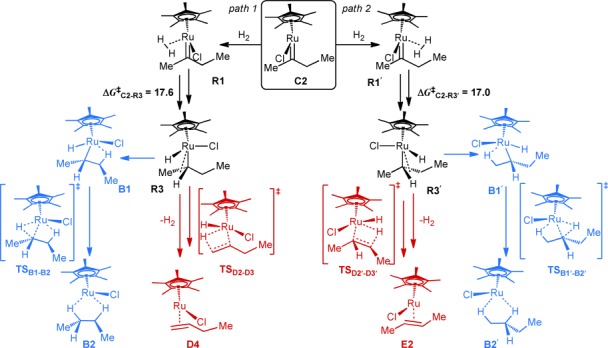
Computed fate of the carbene formed by *geminal* hydrogenation upon addition of a second H_2_ molecule.

**Scheme 4:**
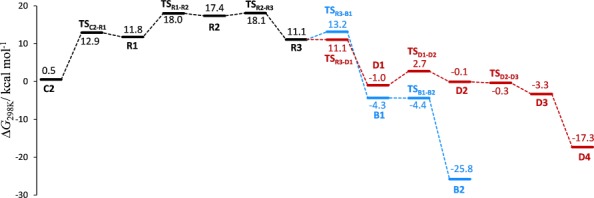
Free energy profile for path 1 from Scheme 3 at 298 K (for the corresponding profile for path 2, see the Supporting Information).

The product spectrum depends on the side from which H_2_ approaches the carbene center. If H_2_ approaches from the side of the methyl group (path 1), adduct **R1** is formed, which evolves into the ruthenium alkyl complex **R3** with an α-agostic interaction. A subsequent barrier-free rotation about the Ru–C_α_ bond swaps the agostic binding sites and brings the β-hydrogen atom of the methyl terminus into the Ru coordination sphere, which opens a gateway (red path in Schemes 3 and 4) to the isomerized side product 1-butene (**D4**). If, instead, the internal β-hydrogen atom engages with the Ru center through yet another low-energy rotatory motion (**R3**→**TS_R3–B1_**→**B1**, Δ*G*^≠^=+2.1 kcal mol^−1^), a subsequent reductive elimination affords the alkane side product **B2** (blue path). Analogously, in case H_2_ approaches from the side of the ethyl group (path 2), the resulting hydrogen adduct **R1′** is converted into the desired *E*-alkene **E2** and the alkane side product **B2′** by processes that are very similar to those described for path 1 (for details, see the Supporting Information).

We thus conclude that the carbene **C2** is linked to the desired product; at the same time, this very intermediate also constitutes the major gateway to the side products. This interpretation is in excellent accord with the ^1^H-OPSY-EXSY experiment shown in Figure 4. The somewhat higher barrier for the formation of **B1** compared with that of **D1** suggests that over-reduction is less of a problem than isomerization, which is again in qualitative agreement with the available preparative data.[8, 9]

In summary, we have shown that the *trans*-hydrogenation of internal alkynes competes with a formal *gem*-hydrogenation process; this latter reactivity mode has hardly any precedent in organic chemistry.[24, 25] The resulting ruthenium carbene intermediates seem to be the very origin of the side reactions that can plague catalytic *trans*-reductions at this stage of methodological development. Our ongoing attempts at optimizing this valuable transformation will be guided by this insight.

## Supporting Information

As a service to our authors and readers, this journal provides supporting information supplied by the authors. Such materials are peer reviewed and may be re-organized for online delivery, but are not copy-edited or typeset. Technical support issues arising from supporting information (other than missing files) should be addressed to the authors.

miscellaneous_information
