# Supplementary material for: Formation of Ruthenium Carbenes by gem-Hydrogen Transfer to Internal Alkynes: Implications for Alkyne trans-Hydrogenation
Source: Angew Chem Int Ed Engl. 2015 Aug 31;54(42):12431–6. doi: 10.1002/anie.201506075 (PMC4643192; doi:10.1002/anie.201506075)
Supplement: Supplementary file 1 — miscellaneous_information [file anie0054-12431-sd1.pdf]

Angewandte Chemie
Eine Zeitschrift der Gesellschaft Deutscher Chemiker

Supporting Information

Formation of Ruthenium Carbenes by *gem*-Hydrogen Transfer to Internal Alkynes: Implications for Alkyne *trans*-Hydrogenation

*Markus Leutzsch, Larry M. Wolf, Puneet Gupta, Michael Fuchs, Walter Thiel, Christophe Farès, and Alois Fürstner**

anie_201506075_sm_miscellaneous_information.pdf

Computational Part

Table of Contents

1. Computational Methods S2

2. Hydrogenation of 2-butyne using the neutral ruthenium catalyst: Description of additional pathways S2

3. Hydrogenation of the alkyne substrate (8b) using the neutral ruthenium catalyst S6

4. Hydrogenation of 2-butyne using the cationic ruthenium catalyst S8

5. Energy table for the reaction of the model substrate (2-butyne) with the neutral Ru(II) catalyst S10

6. Energy table for the reaction of the substrate (8b) with the neutral Ru(II) catalyst S11

7. Energy table for the reaction of the model substrate (2-butyne) with the cationic Ru(II) catalyst S12

8. Comparison of the computed gas-phase structure and the X-ray structure of 9b S14

9. Coordinates S15

10. References S139

1. Computational Methods

Density functional theory (DFT) was used to elucidate the mechanism of the Ru(II) catalyzed hydrogenation of alkynes. All geometry optimizations were performed using the M06[1] functional. The triple-ζ quality def2-TZVP[2] basis set was used for all atoms. The 28 inner-shell core electrons of the ruthenium atom were described by the corresponding def2 effective core potential[3] accounting for scalar relativistic effects (def2-ecp).

Stationary points were characterized by evaluating the harmonic vibrational frequencies at the optimized geometries. Zero-point vibrational energies (ZPVE) were computed from the corresponding harmonic vibrational frequencies without scaling. Relative free energies (ΔG) were determined at standard pressure (1 bar) and at room temperature (298 K). The thermal and entropic contributions were evaluated within the rigid-rotor harmonic-oscillator approximation. Solvation contributions were included for dichloromethane on the optimized gas-phase geometries employing the SMD solvation model[4] using the same functional and basis set. All calculations were performed using Gaussian09 with the ultrafine grid.[5]

2. Hydrogenation of 2-butyne using the neutral ruthenium catalyst: Description of additional pathways

Hydrogenation has experimentally been shown to lead to the formation of side products. The pathways involved are discussed here in some detail.

The association of H_2 to **C2** is uphill in Gibbs free energy by 11.3 kcal/mol (Scheme S1). From the association complex **R1**, oxidative addition of hydrogen affords the dihydride **R2** which readily delivers a hydride to the α-carbon forming **R3** which exhibits an α-agostic interaction.

Intermediate **R3** can undergo facile rotation about the Ru-C bond to enable agostic interactions with either hydrogen at the β position (Scheme S2). The barrier is negligible for formation of an agostic interaction with a primary hydrogen of the methyl group (**$TS_{R3\text{-}D1}$**) affording **D1** which can undergo β-hydride elimination with a low barrier (**$TS_{D1\text{-}D2}$**, 3.7 kcal/mol); from the resulting complex **D2**, molecular hydrogen H_2 can easily be extruded. An alternative low-energy rotation about the Ru-C bond transforms **R3** into **B1** showing an agostic interaction with a secondary hydrogen of the ethyl group (via **$TS_{R3\text{-}B1}$**, 2.1 kcal/mol).

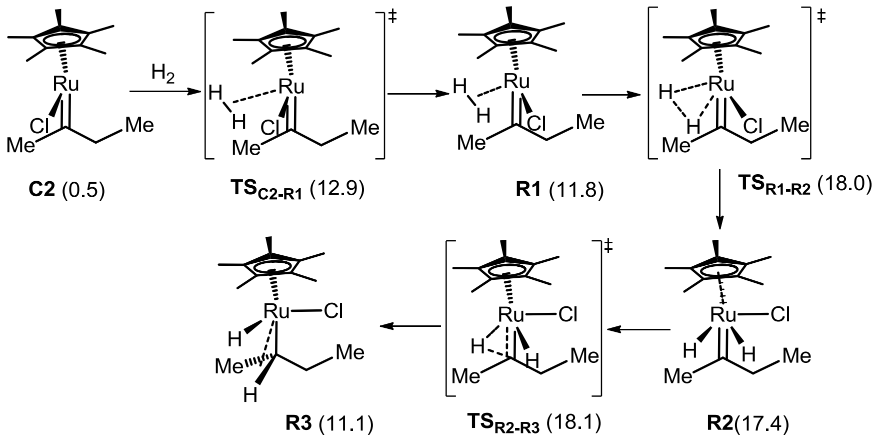

Scheme S1. Detailed mechanism for the addition of H_2 to **C2** from the side of the methyl group. Values in parenthesis are Gibbs free energies in units of kcal·mol^{-1}.

Intermediate **B1** exhibits a strong preference for Ru-H reductive elimination to form the saturated product **B2** rather than β-hydride elimination. By contrast, Ru-H reductive elimination from **D1** is hampered by a less favorable alignment as compared with that in **B1**.

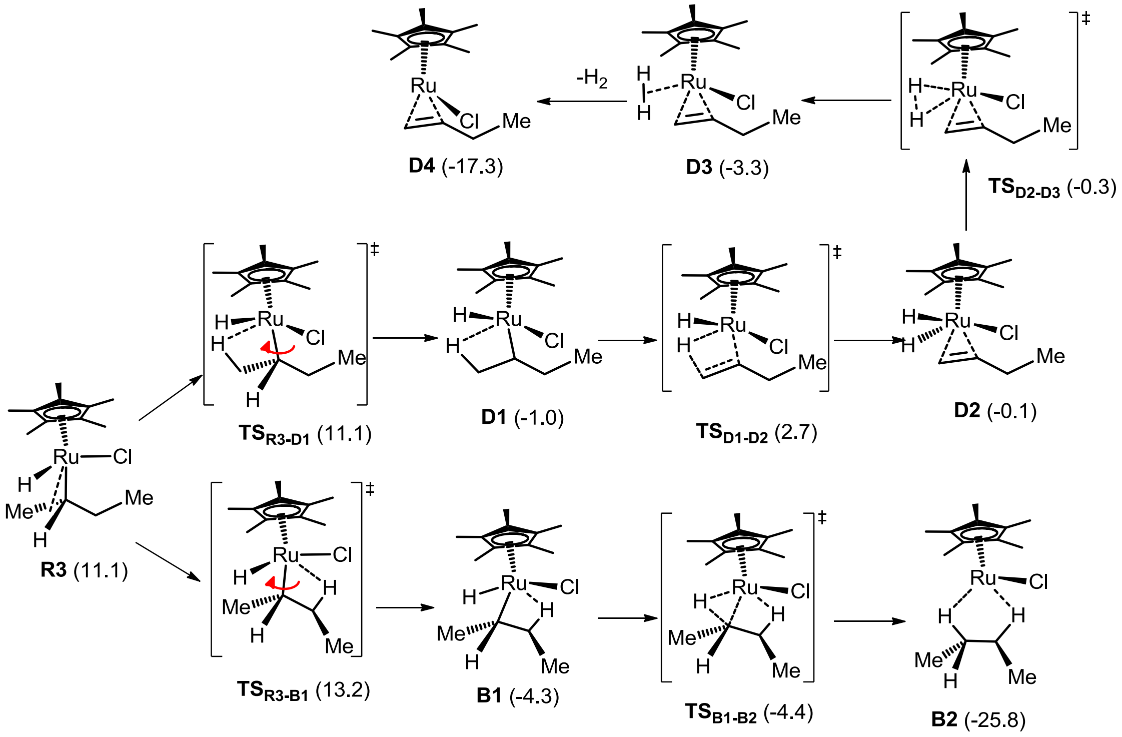

Scheme S2. Detailed mechanism for the conversion of **R3** to the saturated (**B2**) and isomerized (**D4**) products. Values in parenthesis are Gibbs free energies in units of kcal·mol^{-1}.

We also studied the approach of molecular hydrogen from the side of the ethyl group (Schemes S3 and S4). The computed Gibbs free energy profile is shown in Figure S1. The landscape is very similar to that obtained for the approach from the side of the methyl group (see Scheme 3 of the main paper). The approach from the ethyl side yields the saturated alkane **B2′** and the desired *E*-alkene (**E2**) while the approach from the methyl side leads to the isomerized product (**D4**) and the saturated alkane.

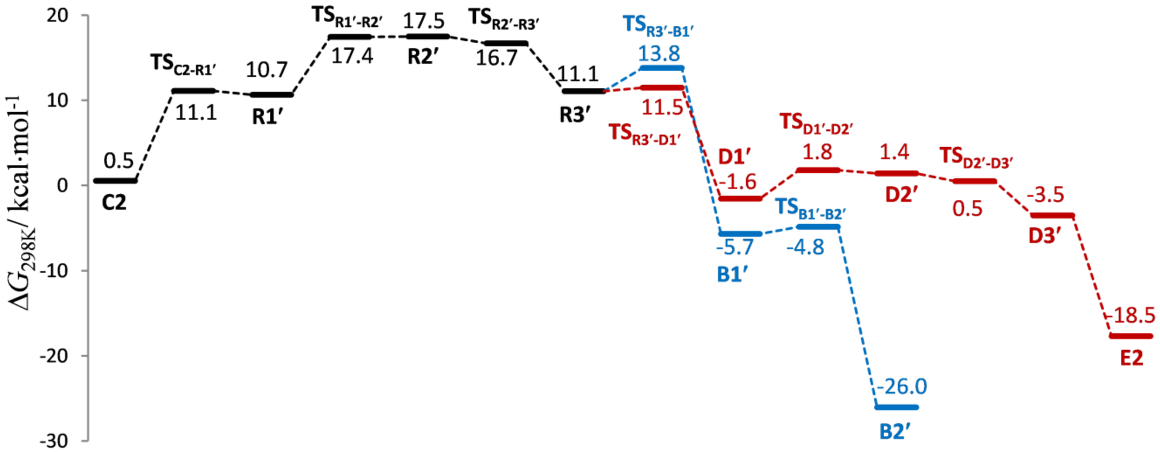

Figure S1. Gibbs free energy profile for addition of H_2 to **C2** from the side of the ethyl group.

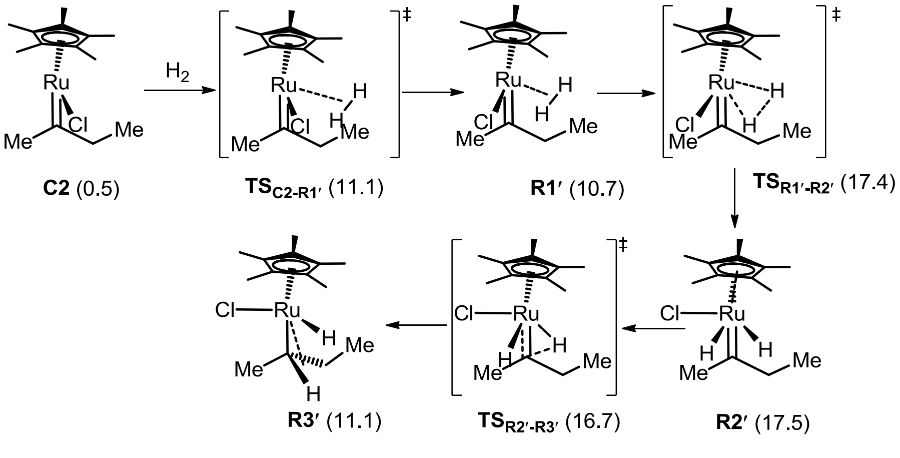

Scheme S3. Detailed mechanism for the addition of H_2 to **C2** from the side of the ethyl group. Values in parenthesis are Gibbs free energies in units of kcal·mol^{-1}.

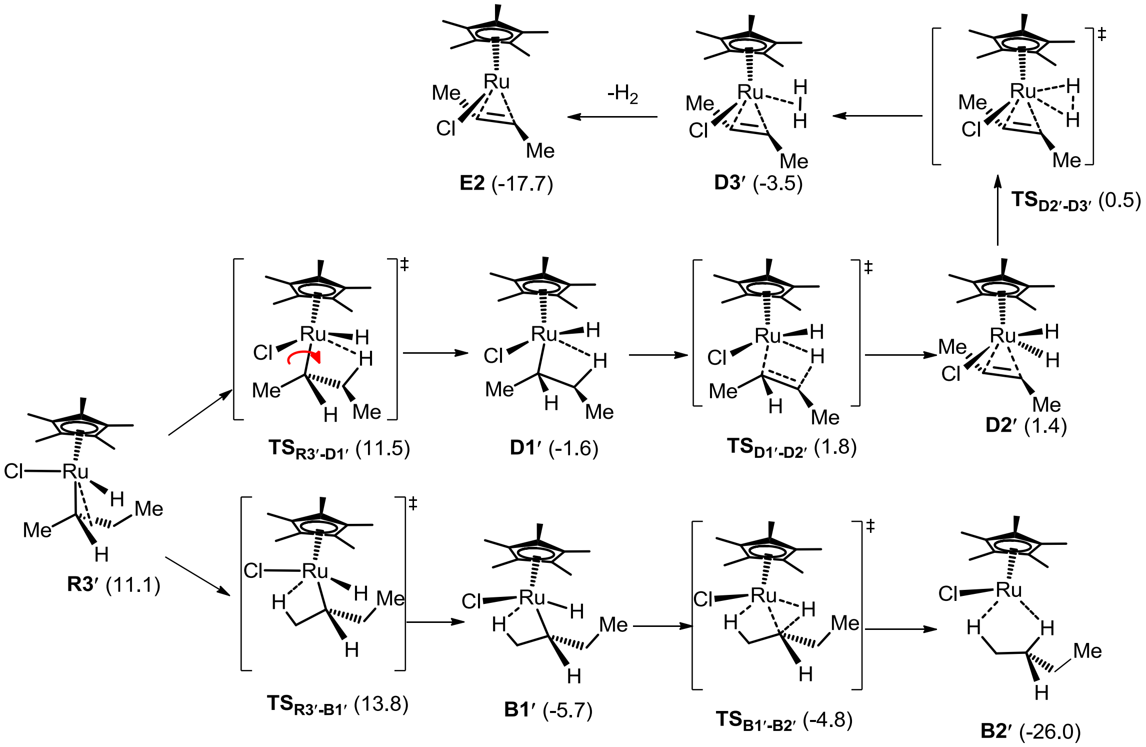

Scheme S4. Detailed mechanism for the conversion of **R3′** to the saturated alkane (**B2′**) and the *trans*-alkene (**E2)** products. Values in parenthesis are Gibbs free energies in units of kcal·mol^{-1}.

There are two further pathways from **C2** that have not yet been discussed in detail, namely direct formation of *trans*-alkene **E2** and isomerization in the absence of H_2. The corresponding Gibbs free energy profiles are displayed in Figure S2. The direct 1,2-hydrogen transfer exhibits the highest barrier of any process investigated presently and is thus unlikely to happen to any measurable extent. The isomerization from **C2** to **D4** involves an initial primary β-hydrogen abstraction by Ru from the methyl group. The computed overall barrier is similar to that for formation of the *cis*-alkene **Z3** (compare $\mathbf{TS_{I1\text{-}I2}}$ with $\mathbf{TS_{Z1\text{-}Z2}}$). Alternative pathways involving molecular hydrogen remain energetically most favorable and will thus be more likely.

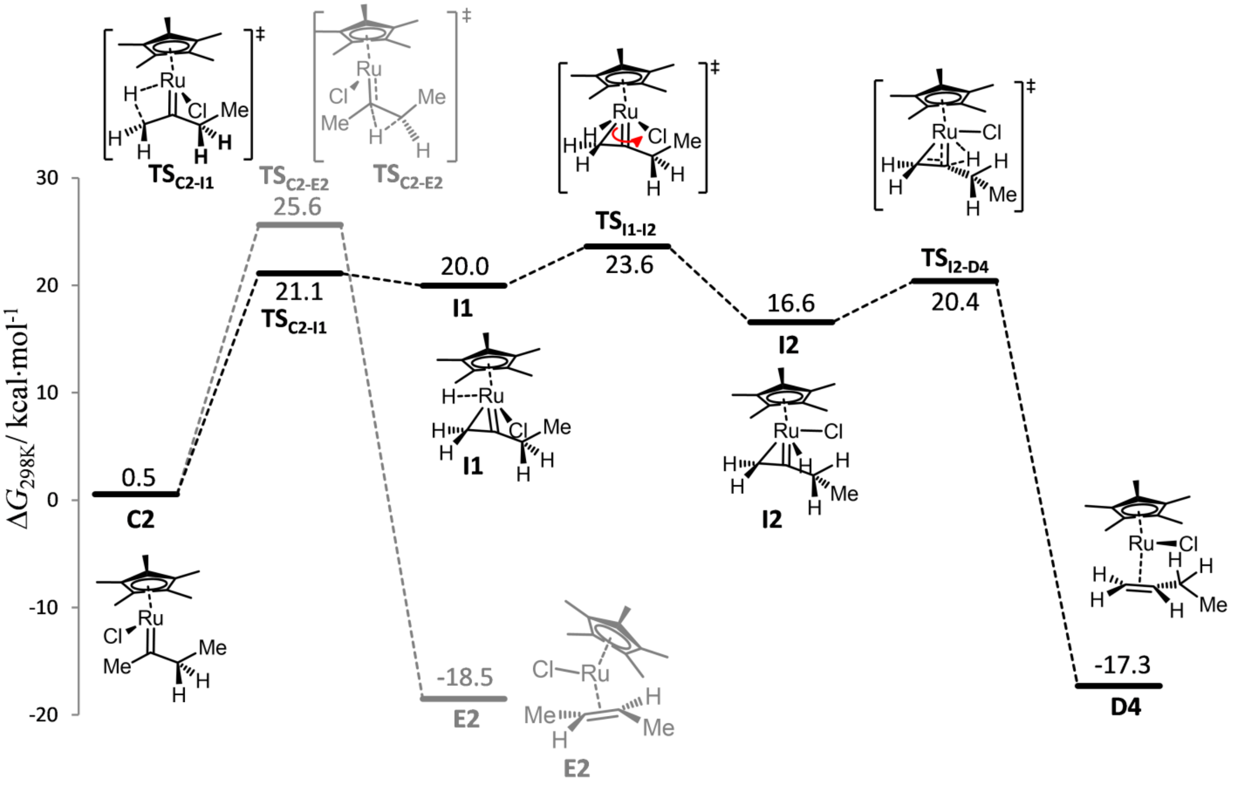

Figure S2. Gibbs free energy profile (in units of kcal·mol^{-1}) for direct formation of **E2** from **C2** and for isomerization in the absence of H_2.

3. Hydrogenation of the alkyne substrate (8b) using the neutral ruthenium catalyst

After the thorough study of all mechanistic pathways for the model substrate 2-butyne using the neutral ruthenium(II) catalyst, we computed selected pathways for the actual substrate **8b** using the same neutral ruthenium catalyst. These pathways are indicated in Schemes S5 and S6. The key results are: (1) The Gibbs free energy barrier for hydride transfer (via $\mathbf{TS_{A2\text{-}A3}}$) is lowered by 8 kcal mol^{-1} compared to the model substrate. This lowering most probably arises from the hydrogen bonding interaction between the hydroxyl group and the chloride ligand. (2) The formation of carbene **C2** from **E1** is preferred by 2.6 kcal mol^{-1} over the competing path that directly leads to *E*-alkene. Therefore, **C2** should mostly be formed from **E1**. (3) In the **E1**→**C2** conversion, an intermediate similar to **C1** (for the model substrate) does not exist. (4) **C2** may adopt a less stable conformation, in which the methoxy group is not coordinated to the Ru center; this destabilizes the carbene **C2'** by 6.6 kcal mol^{-1} relative to **C2**. (5) All the paths originating from **C2** have high energy barriers (in kcal mol^{-1}): $\mathbf{TS_{E1\text{-}E2}}$ (26.6), $\mathbf{TS_{C2\text{-}E2}}$ (31.5), and $\mathbf{TS_{R1'\text{-}R3'}}$ (38.6). Therefore, the highly stable carbene **C2** acts as a thermodynamic sink in this reaction.

These results are in full agreement with the experimental finding that only **C2** is formed when using the actual substrate **8b** with the neutral ruthenium catalyst.

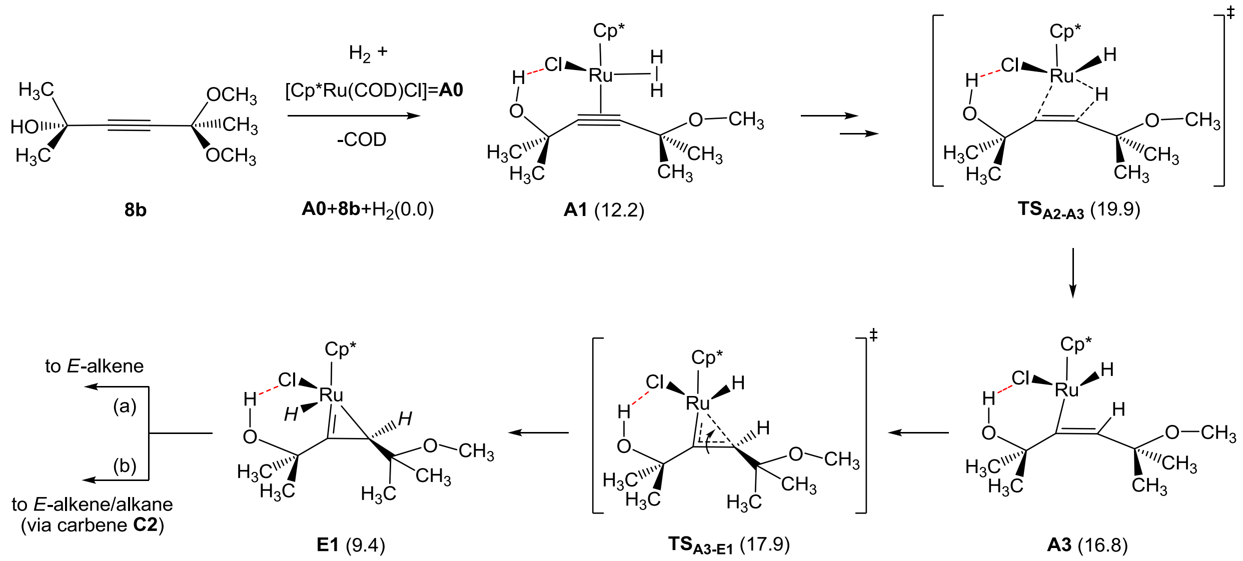

Scheme S5. Formation of the metallacyclic complex **E1**. Hydrogen bonding interactions are shown in red. Gibbs free energies in kcal mol^{-1} are relative to the initial adduct.

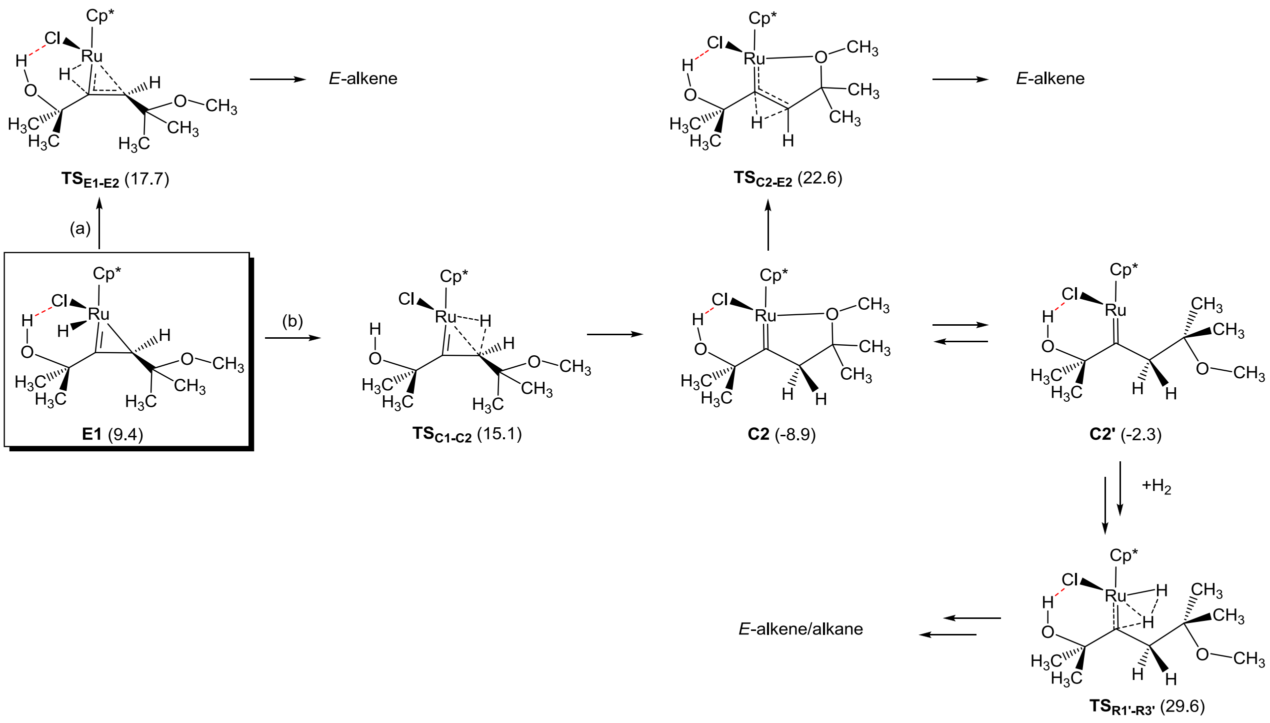

Scheme S6. Pathways from **E1** leading to either *E*-alkene or alkane. Hydrogen bonding interactions are shown in red. Gibbs free energies in kcal mol^{-1} are relative to the initial adduct.

4. Hydrogenation of 2-butyne using the cationic ruthenium catalyst

We also considered the cationic catalyst $[Cp^*Ru(CH_3CN)_3]^+$, in place of the neutral catalyst, to study the change in reactivity towards hydrogenation of 2-butyne (see Figures S3 – S5). We use the same labeling scheme as in the neutral case. Here, we only pinpoint key reactivity differences. In the cationic case, **A1** undergoes H_2 activation and C-H bond formation in a concerted manner via $\mathbf{TS_{A1\text{-}A3}}$ to yield **A3**, whereas the reaction is stepwise in the neutral case (Figure S3). Moreover, the formation of **A3** is more facile in the cationic case due to a reduced barrier of 22.9 kcal mol^{-1}, compared with 27.8 kcal mol^{-1} in the neutral case. Intermediate **C2** is less stable than in the neutral case. The reaction between **C2** and H_2 again involves a concerted H_2 activation and C-H bond formation (via $\mathbf{TS_{R1\text{-}R3}/TS_{R1'\text{-}R3'}}$), which is different from the stepwise process in the neutral case (Figure S4). **C2** is more easily hydrogenated than in the neutral case by 6.0 kcal mol^{-1}. Other than this, we find no major differences.

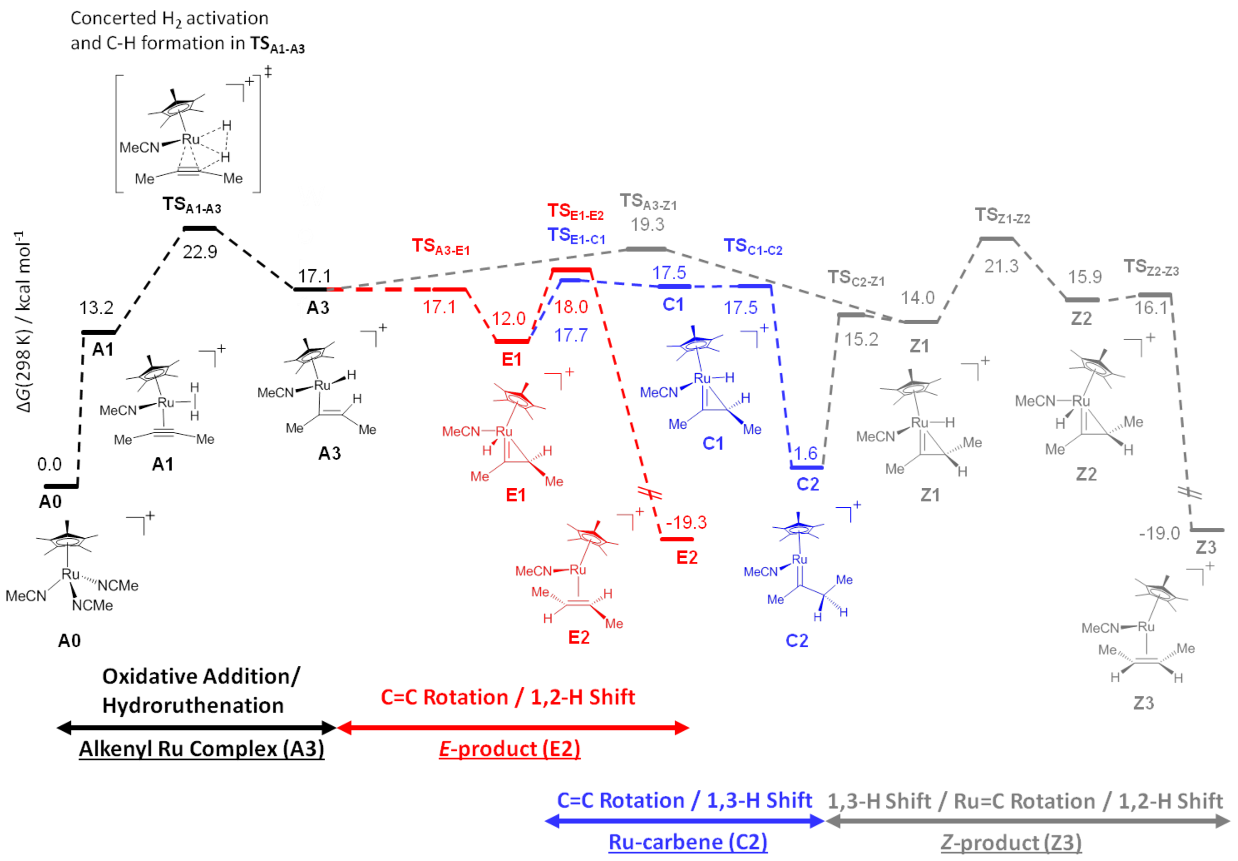

Figure S3. Gibbs free energy profile (in units of kcal·mol^{-1}) for the hydrogenation of 2-butyne with the cationic catalyst.

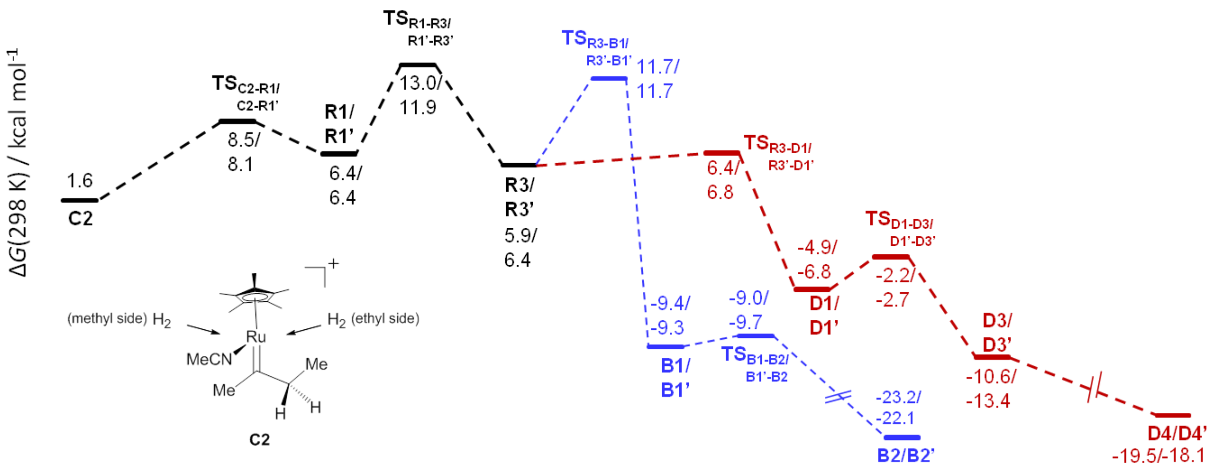

Figure S4. Gibbs free energy profile (in units of kcal·mol^{-1}) for the reaction of carbene **C2** with H_2 in the case of the cationic catalyst. H_2 may approach either from the ethyl or methyl side. Primed (unprimed) labels denote the H_2 addition from the ethyl (methyl) side.

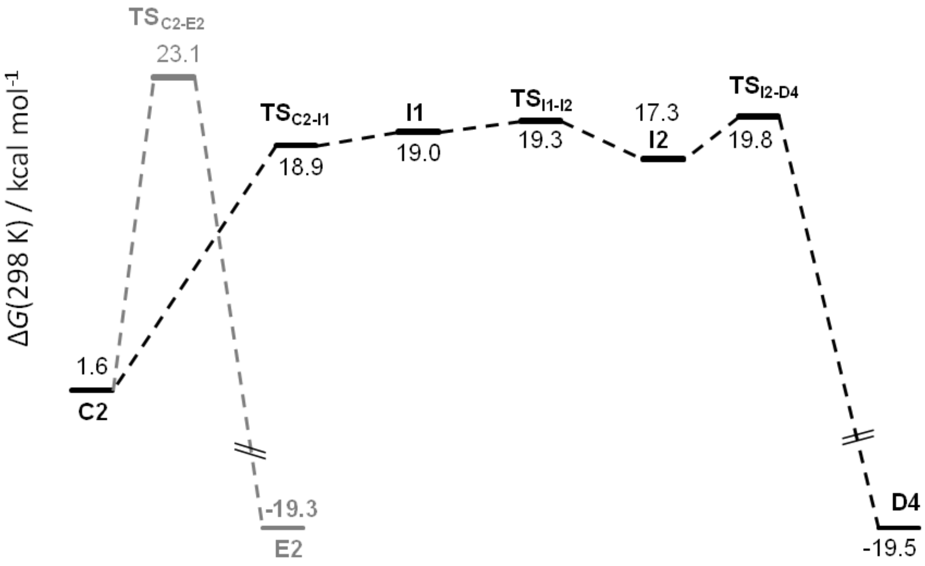

Figure S5. Gibbs free energy profile (in units of kcal·mol^{-1}) for direct formation of **E2** from **C2** and isomerization in the absence of H_2 (in the case of the cationic catalyst).

5. Energy table for the reaction of the model substrate (2-butyne) with the neutral Ru(II) catalyst

Table S1. Listed are the SCF energy, zero point vibrational energy (*ZPVE*), enthalpy correction (H_{corr}), and Gibbs free energy correction (G_{corr}) determined on the gas-phase geometries for all stationary points calculated using the neutral Ru catalyst with the 2-butyne substrate. The single imaginary frequency (υ_i cm^{-1}) is also listed for all transition states. Single point solvent (DCM) corrected SCF energies on the gas phase geometries are also documented. All energies are in atomic units.

	SCF_{gas}	SCF_{DCM}	*ZPVE*	H_{corr}	G_{corr}	υ_i (cm^{-1})
**H_2**	-1.170676	-1.170379	0.009802	0.013160	-0.001642	
**COD**[a]	-311.885137	-311.895992	0.179556	0.187981	0.148036	
**2-butyne**	-155.909153	-155.916818	0.084023	0.090553	0.054557	
**A0**	-1257.045711	-1257.069358	0.405931	0.429301	0.357267	
**A1**	-1102.221195	-1102.244527	0.325399	0.348672	0.275921	
**TS_{A1-A2}**	-1102.210663	-1102.233644	0.323785	0.346078	0.276381	*i*242
**A2**	-1102.212742	-1102.235656	0.325984	0.348381	0.278153	
**TS_{A2-A3}**	-1102.205382	-1102.229081	0.323508	0.345889	0.275004	*i*460
**A3**	-1102.215509	-1102.241864	0.327269	0.349804	0.278601	
**TS_{A3-E1}**	-1102.215400	-1102.242330	0.327108	0.348993	0.279117	*i*31
**E1**	-1102.231583	-1102.254222	0.328121	0.350319	0.279811	
**TS_{E1-E2}**	-1102.223233	-1102.245404	0.327278	0.348907	0.279170	*i*37
**E2**	-1102.287611	-1102.312147	0.332766	0.354917	0.284219	
**TS_{E1-C1}**	-1102.220078	-1102.243464	0.327047	0.348985	0.279224	*i*644
**C1**	-1102.223333	-1102.246574	0.327793	0.350302	0.278611	
**TS_{C1-C2}**	-1102.221485	-1102.244794	0.326783	0.348953	0.277775	*i*449
**C2**	-1102.252791	-1102.278138	0.331010	0.353699	0.280593	
**TS_{C2-Z1}**	-1102.225181	-1102.249585	0.327354	0.349208	0.279344	*i*557
**Z1**	-1102.228292	-1102.252327	0.328087	0.350438	0.279260	
**TS_{Z1-Z2}**	-1102.217448	-1102.240521	0.327720	0.349334	0.280024	*i*44
**Z2**	-1102.226201	-1102.248598	0.327958	0.350338	0.279233	
**TS_{Z2-Z3}**	-1102.224513	-1102.246874	0.326795	0.348798	0.278856	*i*481
**Z3**	-1102.284838	-1102.309165	0.333049	0.355035	0.285069	
**TS_{A3-Z1}**	-1102.210127	-1102.237070	0.327045	0.348885	0.279276	*i*179
**TS_{C2-R1}**	-1103.420214	-1103.444479	0.344606	0.368439	0.294651	*i*215
**R1**	-1103.428659	-1103.451036	0.348876	0.372025	0.299383	
**TS_{R1-R2}**	-1103.419613	-1103.441294	0.347725	0.369841	0.299586	*i*33
**R2**	-1103.419820	-1103.441285	0.348038	0.370895	0.298507	
**TS_{R2-R3}**	-1103.417505	-1103.439549	0.346722	0.369267	0.297909	*i*470
**R3**	-1103.430921	-1103.455571	0.351464	0.373784	0.302813	
**TS_{R3-D1}**	-1103.425875	-1103.455841	0.351174	0.373233	0.303033	*i*89
**D1**	-1103.451315	-1103.476033	0.352164	0.374262	0.304014	
**TS_{D1-D2}**	-1103.444955	-1103.467209	0.348903	0.370770	0.301143	*i*487

**D2**	-1103.452982	-1103.473980	0.350858	0.372795	0.303441	
**TS$_{D2-D3}$**	-1103.447956	-1103.469803	0.347620	0.369864	0.298833	*i*818
**D3**	-1103.454030	-1103.477232	0.350487	0.373075	0.301559	
**D4**	-1102.285234	-1102.310098	0.333176	0.355130	0.284134	
**TS$_{R3-B1}$**	-1103.424607	-1103.450819	0.350590	0.373113	0.301364	*i*154
**B1**	-1103.457568	-1103.481602	0.352055	0.374276	0.304278	
**TS$_{B1-B2}$**	-1103.456506	-1103.480897	0.351097	0.373033	0.303507	*i*457
**B2**	-1103.484055	-1103.512464	0.354820	0.378519	0.300968	
**TS$_{C2-R1'}$**	-1103.420451	-1103.445667	0.343979	0.368124	0.292892	*i*197
**R1′**	-1103.429110	-1103.451967	0.348494	0.371757	0.298501	
**TS$_{R1'-R2'}$**	-1103.418818	-1103.440292	0.346148	0.368765	0.297657	*i*144
**R2′**	-1103.419326	-1103.441121	0.347628	0.370541	0.298564	
**TS$_{R2'-R3'}$**	-1103.418023	-1103.440477	0.345923	0.368769	0.296647	*i*414
**R3′**	-1103.429657	-1103.455357	0.351081	0.373528	0.302535	
**TS$_{R3'-D1'}$**	-1103.425761	-1103.455413	0.351212	0.373277	0.303243	*i*80
**D1′**	-1103.452310	-1103.475961	0.351451	0.373752	0.303036	
**TS$_{D1'-D2'}$**	-1103.447590	-1103.469110	0.348598	0.370517	0.301516	*i*421
**D2′**	-1103.450916	-1103.471173	0.350335	0.372512	0.302996	
**TS$_{D2'-D3'}$**	-1103.448324	-1103.469608	0.347627	0.369821	0.299987	*i*378
**D3′**	-1103.462960	-1103.478114	0.350059	0.372783	0.302087	
**TS$_{R3'-B1'}$**	-1103.424333	-1103.450848	0.351150	0.373467	0.302406	*i*152
**B1′**	-1103.457099	-1103.482398	0.351734	0.374151	0.302908	
**TS$_{B1'-B2'}$**	-1103.456013	-1103.481651	0.351236	0.373098	0.303495	*i*465
**B2′**	-1103.483869	-1103.512446	0.354664	0.378474	0.300507	
**TS$_{C2-E2}$**	-1102.209797	-1102.235204	0.327338	0.349631	0.277639	*i*994
**TS$_{C2-I1}$**	-1102.220521	-1102.243633	0.327609	0.349424	0.278880	*i*534
**I1**	-1102.222828	-1102.246718	0.328729	0.350766	0.280106	
**TS$_{I1-I2}$**	-1102.217983	-1102.240553	0.328157	0.349565	0.279796	*i*70
**I2**	-1102.227584	-1102.250099	0.328132	0.350345	0.278086	
**TS$_{I2-D4}$**	-1102.223730	-1102.245335	0.327601	0.349264	0.279422	*i*559

[a] COD = 1,5-cyclooctadiene.

6. Energy table for the reaction of the substrate (8b) with the neutral Ru(II) catalyst

Table S2. Listed are the SCF energy, zero point vibrational energy (*ZPVE*), enthalpy correction (H_{corr}), and Gibbs free energy correction (G_{corr}) computed on the gas-phase geometries from Schemes S5 and S6. Single imaginary frequencies (υ_i cm^{-1}) are also listed for all transition states. Single point solvent (DCM) corrected SCF energies on the gas phase geometries are also documented. All energies are in atomic units.

	SCF_{gas}	SCF_{DCM}	*ZPVE*	H_{corr}	G_{corr}	υ_i (cm^{-1})
**H_2**	-1.170676	-1.170379	0.009802	0.013160	-0.001642	
**COD**	-311.885137	-311.895992	0.179556	0.187981	0.148036	

**Substrate**	-502.805165	-502.819957	0.233500	0.248606	0.191658	
**A0**	-1257.045711	-1257.069358	0.405931	0.429301	0.357267	
**A1**	-1449.139002	-1449.162408	0.475617	0.507069	0.417400	
**TS_{A2-A3}**	-1449.126607	-1449.150317	0.474051	0.504514	0.417562	*i*313
**A3**	-1449.132942	-1449.158146	0.477355	0.507926	0.420437	
**TS_{A3-E1}**	-1449.129482	-1449.155627	0.476606	0.506942	0.419750	*i*234
**E1**	-1449.146867	-1449.171280	0.478657	0.508992	0.421766	
**TS_{E1-E2}**	-1449.132799	-1449.158078	0.477911	0.507677	0.421819	*i*766
**TS_{C1-C2}**	-1449.134020	-1449.158766	0.476184	0.506955	0.418316	*i*492
**C2**	-1449.181467	-1449.204808	0.482170	0.512437	0.426129	
**C2'**	-1449.163498	-1449.189301	0.480743	0.511913	0.421203	
**TS_{C2-E2}**	-1449.120852	-1449.149210	0.477188	0.507521	0.420753	*i*1155
**$TS_{R1'-R3'}$**	-1450.304812	-1450.328645	0.496700	0.527670	0.439382	

7. Energy table for the reaction of the model substrate (2-butyne) with the cationic Ru(II) catalyst

Table S3. Listed are the SCF energy, zero point vibrational energy (*ZPVE*), enthalpy correction (H_{corr}), and Gibbs free energy correction (G_{corr}) computed on the gas-phase geometries from Figures S3, S4, and S5. Single imaginary frequencies (υ_i cm^{-1}) are also listed for all transition states. Single point solvent (DCM) corrected SCF energies on the gas phase geometries are also documented. All energies are in atomic units.

	SCF_{gas}	SCF_{DCM}	*ZPVE*	H_{corr}	G_{corr}	υ_i (cm^{-1})
**H_2**	-1.170676	-1.170379	0.009802	0.013160	-0.001642	
**CH_3CN**	-132.709408	-132.719151	0.045207	0.049731	0.021232	
**Substrate**	-155.909153	-155.916818	0.084023	0.090553	0.054557	
**A0**	-882.893965	-882.966911	0.363915	0.392357	0.303341	
**A1**	-774.526044	-774.599262	0.372269	0.398281	0.318301	
**TS_{A1-A3}**	-774.509312	-774.581394	0.369200	0.394765	0.315863	*i*648
**A3**	-774.520908	-774.595117	0.373835	0.399315	0.320423	
**TS_{A3-E1}**	-774.520894	-774.595966	0.373519	0.398288	0.321125	*i*51
**E1**	-774.530419	-774.604269	0.374734	0.399891	0.321392	
**TS_{E1-E2}**	-774.520281	-774.594635	0.373982	0.398637	0.321233	*i*643
**E2**	-774.582284	-774.659706	0.379505	0.404424	0.326956	
**TS_{E1-C1}**	-774.519668	-774.593589	0.373391	0.398156	0.319854	*i*69
**C1**	-774.523392	-774.597224	0.375048	0.399966	0.323069	
**TS_{C1-C2}**	-774.521506	-774.594664	0.373443	0.398393	0.320599	*i*425
**C2**	-774.546072	-774.621932	0.377198	0.402851	0.322441	
**TS_{C2-Z1}**	-774.525462	-774.598846	0.373699	0.398463	0.321099	*i*607
**Z1**	-774.529181	-774.602270	0.374855	0.39986	0.322603	
**TS_{Z1-Z2}**	-774.515719	-774.589987	0.373936	0.398451	0.321969	*i*59
**Z2**	-774.523753	-774.597899	0.374443	0.399758	0.321193	

**TS$_{Z2-Z3}$**	-774.522535	-774.596607	0.373253	0.398274	0.320235	*i*393
**Z3**	-774.579904	-774.657669	0.379353	0.404399	0.325300	
**TS$_{A3-Z1}$**	-774.516821	-774.591048	0.373486	0.398338	0.319774	*i*157
**TS$_{C2-R1}$**	-775.717822	-775.795183	0.390198	0.41745	0.334643	*i*113
**R1**	-775.729650	-775.803949	0.394955	0.421201	0.340151	
**TS$_{R1-R3}$**	-775.718458	-775.791396	0.392281	0.418098	0.338030	*i*347
**R3**	-775.734265	-775.810462	0.398205	0.423281	0.345756	
**TS$_{R3-D1}$**	-775.728209	-775.809622	0.397955	0.422697	0.345857	*i*93
**D1**	-775.754758	-775.829239	0.399246	0.423958	0.347453	
**TS$_{D1-D3}$**	-775.746630	-775.820523	0.394944	0.419689	0.342982	*i*845
**D3**	-775.759615	-775.834197	0.396709	0.422502	0.343284	
**D4**	-774.579119	-774.657169	0.379276	0.404380	0.324132	
**TS$_{R3-B1}$**	-775.719999	-775.799535	0.397327	0.422607	0.344145	*i*182
**B1**	-775.759802	-775.833804	0.397811	0.423289	0.344855	
**TS$_{B1-B2}$**	-775.759409	-775.833575	0.397595	0.422486	0.345126	*i*387
**B2**	-775.782106	-775.858228	0.401953	0.427617	0.347301	
**TS$_{C2-R1'}$**	-775.718051	-775.794339	0.389638	0.417200	0.333202	*i*117
**R1'**	-775.730544	-775.804339	0.394914	0.421093	0.340559	
**TS$_{R1'-R3'}$**	-775.719350	-775.792602	0.391613	0.417605	0.337488	*i*597
**R3'**	-775.734070	-775.809799	0.398158	0.423248	0.345948	
**TS$_{R3'-D1'}$**	-775.728434	-775.809782	0.398178	0.422840	0.346540	*i*93
**D1'**	-775.755857	-775.829754	0.398882	0.423720	0.344870	
**TS$_{D1'-D3'}$**	-775.748831	-775.822172	0.394269	0.418374	0.343835	*i*628
**D3'**	-775.764354	-775.838849	0.396605	0.422376	0.343432	
**D4'**	-774.582272	-774.659709	0.379429	0.403471	0.328855	
**TS$_{R3'-B1'}$**	-775.719771	-775.799347	0.396796	0.421549	0.344014	*i*187
**B1'**	-775.759460	-775.834324	0.398499	0.423636	0.345491	
**TS$_{B1'-B2'}$**	-775.759054	-775.833791	0.397325	0.422305	0.344221	*i*371
**B2'**	-775.781313	-775.858117	0.401843	0.426607	0.348884	
**TS$_{C2-E2}$**	-774.506161	-774.583579	0.373600	0.399089	0.318348	*i*976
**TS$_{C2-I1}$**	-774.518858	-774.592291	0.373595	0.398336	0.320337	*i*545
**I1**	-774.520358	-774.593636	0.374874	0.399720	0.321949	
**TS$_{I1-I2}$**	-774.518760	-774.592790	0.374091	0.398268	0.321586	*i*50
**I2**	-774.523130	-774.597439	0.375224	0.399935	0.323057	
**TS$_{I2-D4}$**	-774.517773	-774.592210	0.373833	0.398355	0.321707	*i*568

8. Comparison of the computed gas-phase structure and the X-ray structure of 9b

Table S4. Selected bond distances (Å) and angles (°) of structure **9b** (X-ray) **/ C2** (DFT). M06/def2-TZVP (ultrafine grid) was used for geometry optimization. The DFT computed geometry is in close agreement with the X-ray derived structure. Hydrogen atoms are removed for clarity.

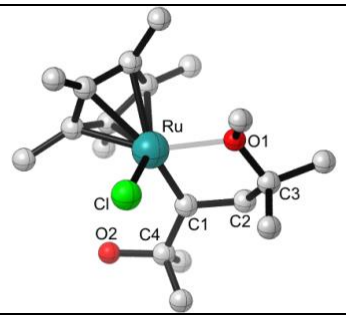

Geometry structure		X-ray	DFT
Bond	Ru-C1	1.88	1.89
	Ru-Cl	2.41	2.42
	Ru-O1	2.23	2.31
	C1-C2	1.52	1.51
	C2-C3	1.52	1.53
	C3-O1	1.46	1.44
	C1-C4	1.52	1.51
	C4-O2	1.43	1.41
Angle	Ru-C1-C2	115.6	115.8
	C1-C2-C3	112.3	112.8
	C2-C3-O1	102.9	103.6
	Ru-C1-C4	128.6	128.2
	C1-C4-O2	110.9	111.6

9. Coordinates

All graphics included in this section were generated using the CYLview program.[6]

XYZ coordinates (Å) for the molecules present in the hydrogenation of 2-butyne using the neutral catalyst

A0

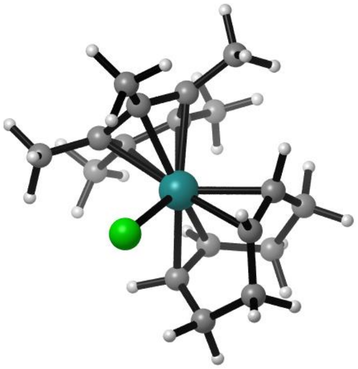

Ru	0.777064	1.278906	1.443995
Cl	-0.101411	1.196073	-0.865948
C	0.115427	3.449149	1.463179
C	1.410460	3.331773	0.827023
C	2.330327	2.843646	1.773277
C	1.614112	2.613590	3.000993
C	0.262396	3.050870	2.805823
C	-1.100957	4.002884	0.818649
H	-1.985267	3.861381	1.441064
H	-1.283123	3.514697	-0.141720
H	-0.989063	5.076549	0.637005
C	1.762533	3.782686	-0.541526
H	0.889505	3.832377	-1.189202
H	2.474399	3.101756	-1.012137
H	2.223394	4.775831	-0.497542
C	3.796543	2.723912	1.555866
H	4.298468	2.240048	2.394163
H	4.245709	3.714908	1.437237
H	4.028652	2.156858	0.651136
C	2.241044	2.291830	4.310985
H	1.504073	1.959869	5.042880
H	2.735671	3.175907	4.726656
H	2.996958	1.508592	4.228055
C	-0.777305	3.142053	3.861300
H	-1.780066	2.994767	3.456801
H	-0.755032	4.132021	4.327303
H	-0.624408	2.408118	4.654282
H	-0.175543	-2.442896	1.704246
C	-0.231566	-1.670469	0.930064
C	1.112123	-1.539123	0.202746
H	-0.984693	-2.014155	0.218565
C	-0.718548	-0.384041	1.550267
C	1.959354	-0.418830	0.728752
H	1.666388	-2.485873	0.259763
H	0.926662	-1.347680	-0.854283
C	2.224946	-0.225392	2.090015
H	2.668552	-0.007606	0.012909
C	1.753057	-1.166131	3.180204

H	3.138428	0.311321	2.326455
C	0.445434	-0.704815	3.811017
H	1.646727	-2.174343	2.769738
H	2.526363	-1.242173	3.949419
H	0.661947	-0.043825	4.655990
C	-0.426009	0.040440	2.839015
H	-0.108402	-1.554324	4.234275
H	-1.150476	0.706167	3.299187
H	-1.598860	0.047844	1.088297

A1

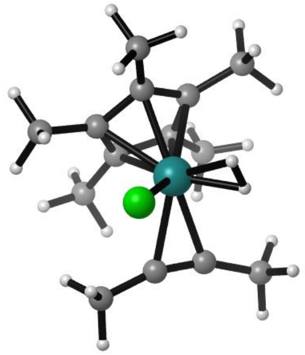

Ru	0.494118	1.639484	1.367353
Cl	-1.754135	1.266784	0.494887
C	1.064388	0.625319	-0.609384
C	1.656229	0.016799	0.283809
H	-0.298007	0.609256	2.557482
H	0.318852	0.110212	2.263078
C	2.486557	-0.991405	0.938778
C	0.527529	1.048533	-1.894662
C	0.417360	3.813813	1.062701
C	1.741726	3.373919	0.796008
C	2.272463	2.768550	1.983353
C	1.266064	2.851710	2.989025
C	0.105837	3.482995	2.427391
C	-0.503026	4.512896	0.132520
H	-1.491144	4.047762	0.147460
H	-0.140807	4.477859	-0.895766
H	-0.614041	5.564099	0.416170
C	2.522908	3.571877	-0.451454
H	1.902631	3.940839	-1.268155
H	3.000170	2.645262	-0.779707
H	3.318253	4.304606	-0.283445
C	3.670247	2.293297	2.148382
H	3.761553	1.590941	2.978995
H	4.351218	3.127725	2.348530
H	4.029901	1.792029	1.246969
C	1.411650	2.391595	4.395203
H	0.448443	2.117643	4.828008
H	1.842606	3.180014	5.019699
H	2.065261	1.520029	4.463301
C	-1.156131	3.846214	3.122561
H	-2.020917	3.625612	2.493919
H	-1.180147	4.912492	3.370889
H	-1.276385	3.286452	4.051224
H	-0.563409	1.057187	-1.851111
H	0.850139	0.370063	-2.688683
H	0.858044	2.058120	-2.152414
H	3.184971	-0.539980	1.647458
H	3.063577	-1.559354	0.204181
H	1.867518	-1.695875	1.500799

TS$_{A1\text{-}A2}$

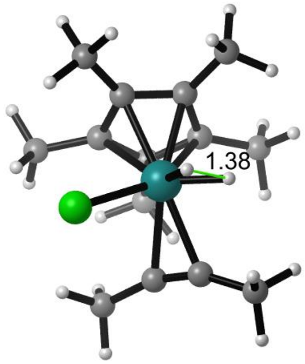

Ru	-0.218908	0.164546	-0.484334
Cl	-0.890211	-2.073672	-1.152513
C	-2.239939	0.024219	0.526188
C	-2.374164	0.847416	-0.380502
H	-0.381352	0.308265	-2.073003
H	-0.447370	1.473428	-1.343999
C	-3.023368	1.793243	-1.282807
C	-2.531285	-0.951332	1.568103
C	1.242468	-0.735314	1.047389
C	0.790025	0.477511	1.603432
C	1.058589	1.525732	0.672595
C	1.789154	0.955132	-0.432336
C	1.870303	-0.448623	-0.224645
C	1.157496	-2.091901	1.642399
H	0.615614	-2.767868	0.973060
H	0.643877	-2.083402	2.604373
H	2.157242	-2.507580	1.797930
C	0.171331	0.702930	2.933810
H	-0.033586	-0.230327	3.457757
H	-0.763737	1.263810	2.854476
H	0.844178	1.293648	3.563361
C	0.803091	2.967950	0.931512
H	0.876771	3.557393	0.016916
H	1.520811	3.377089	1.650735
H	-0.197794	3.121862	1.341924
C	2.422924	1.712041	-1.544714
H	2.445329	1.123541	-2.462413
H	3.453500	1.978458	-1.291480
H	1.884554	2.635773	-1.760652
C	2.581384	-1.449454	-1.058221
H	1.973209	-2.347132	-1.186322
H	3.529444	-1.741426	-0.594264
H	2.800696	-1.055860	-2.051080
H	-1.975847	-0.756240	2.487261
H	-2.265184	-1.949009	1.210116
H	-3.597547	-0.938681	1.807950
H	-2.565870	2.783496	-1.215059
H	-4.085464	1.887223	-1.044835
H	-2.928434	1.460308	-2.318371

A2

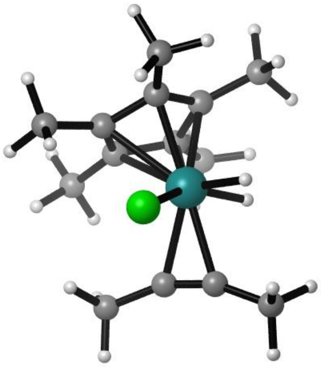

Ru	1.013048	1.455197	1.328169
Cl	-1.200607	0.742767	0.608675
C	1.628861	0.525199	-0.600949
C	1.626701	-0.385740	0.232024
H	0.691540	0.358857	2.429488
H	2.304739	0.809614	1.968793
C	1.650946	-1.719856	0.814385
C	1.591213	1.168855	-1.907556
C	0.220027	3.641527	1.024359
C	1.561765	3.663872	0.620551
C	2.357496	3.148999	1.692475
C	1.485632	2.913879	2.821777
C	0.152930	3.159735	2.388524
C	-0.957464	4.072435	0.236202
H	-1.665782	3.244657	0.128644
H	-0.676893	4.415963	-0.759777
H	-1.475530	4.891181	0.744019
C	2.105034	4.205275	-0.649850
H	1.398148	4.117346	-1.475362
H	3.028585	3.701738	-0.940185
H	2.344195	5.267453	-0.533653
C	3.844582	3.124294	1.697917
H	4.231596	2.544359	2.535777
H	4.252689	4.137670	1.773147
H	4.236130	2.676830	0.781825
C	1.915927	2.587709	4.207179
H	1.153400	2.013284	4.733616
H	2.102336	3.502510	4.778146
H	2.832153	1.996376	4.215042
C	-1.088082	3.112464	3.201786
H	-1.894776	2.631350	2.645112
H	-1.414489	4.121118	3.475950
H	-0.938231	2.546064	4.121276
H	1.707764	0.424043	-2.698745
H	2.378614	1.914426	-2.031063
H	0.626708	1.667330	-2.047132
H	2.358017	-1.782157	1.644167
H	1.923747	-2.465249	0.063528
H	0.659804	-1.962946	1.205467

$TS_{A2\text{-}A3}$

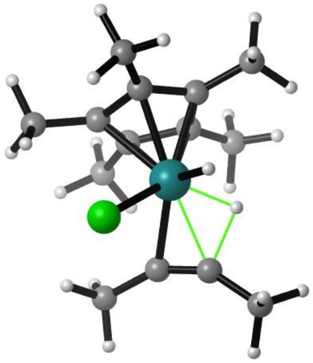

Ru	-0.227446	0.416746	0.084648
Cl	-1.284378	0.835405	-2.061778
C	-2.036053	-0.712172	0.365763
C	-2.230171	0.318295	1.059339
H	-0.319826	2.008039	0.024221
H	-0.907287	1.027833	1.424623
C	-3.156748	1.254846	1.712698
C	-2.498059	-1.913999	-0.324392
C	1.383036	-0.868488	-0.890442
C	1.076858	-1.499364	0.334814
C	1.269641	-0.553866	1.387057
C	1.787456	0.655309	0.807597
C	1.825087	0.475925	-0.612421
C	1.306834	-1.457700	-2.249892
H	0.682252	-0.838231	-2.899761
H	0.879539	-2.460903	-2.233540
H	2.302576	-1.523108	-2.698480
C	0.673247	-2.912785	0.540823
H	0.257298	-3.357459	-0.363631
H	-0.067009	-3.008191	1.338057
H	1.540073	-3.513216	0.835340
C	1.105147	-0.849639	2.834926
H	1.096866	0.064670	3.429260
H	1.918206	-1.481303	3.207186
H	0.166869	-1.375687	3.027268
C	2.292299	1.834937	1.560116
H	2.224975	2.744196	0.962535
H	3.340145	1.695749	1.843773
H	1.719567	2.002795	2.473253
C	2.348491	1.417049	-1.635817
H	1.690570	1.444349	-2.506546
H	3.346653	1.118472	-1.972506
H	2.415292	2.432423	-1.244153
H	-1.995893	-2.813073	0.039774
H	-2.284736	-1.814641	-1.392411
H	-3.574169	-2.056029	-0.194601
H	-2.843431	1.509399	2.727857
H	-4.159529	0.826759	1.755283
H	-3.199750	2.184018	1.138115

A3

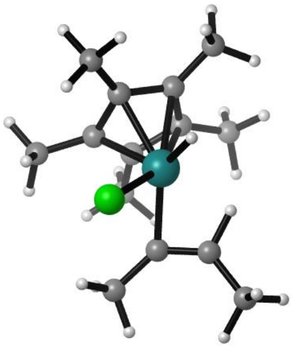

Ru	1.191934	0.451749	-1.871737
Cl	3.573876	0.294798	-2.267313
C	1.857564	1.072537	-0.027706
C	1.433541	-0.074613	0.436587
H	1.364432	-0.989395	-2.572978
H	0.729637	-0.652394	-0.260186
C	1.850754	-0.886973	1.615546
C	2.742868	2.131055	0.468822
C	0.849589	2.318569	-2.892401
C	-0.142700	2.277115	-1.863602
C	-0.951968	1.128294	-2.065437
C	-0.509785	0.471699	-3.255472
C	0.611370	1.198428	-3.774986
C	1.879183	3.364582	-3.116026
H	2.845446	2.909095	-3.341731
H	2.010632	3.995432	-2.235919
H	1.602645	4.011060	-3.954868
C	-0.360356	3.274825	-0.784857
H	0.485263	3.954417	-0.681892
H	-0.526157	2.795454	0.182308
H	-1.245431	3.879487	-1.005483
C	-2.091483	0.727945	-1.201697
H	-2.363824	-0.316658	-1.356333
H	-2.977908	1.336140	-1.409543
H	-1.849953	0.858141	-0.144089
C	-1.173337	-0.699836	-3.886818
H	-0.492392	-1.231523	-4.550814
H	-2.039347	-0.381408	-4.475502
H	-1.523392	-1.413228	-3.139597
C	1.349479	0.946320	-5.038142
H	2.423049	1.071071	-4.887639
H	1.028500	1.637952	-5.823665
H	1.188732	-0.071230	-5.395329
H	2.226998	3.094891	0.510463
H	3.594641	2.236988	-0.208071
H	3.115892	1.912107	1.474947
H	0.994849	-1.250748	2.188622
H	2.481513	-0.292247	2.278235
H	2.426796	-1.759385	1.296024

$TS_{A3\text{-}E1}$

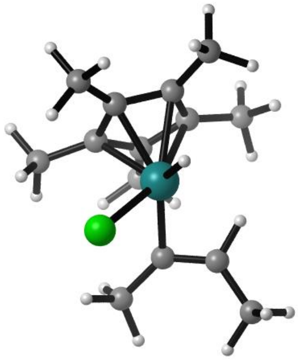

Ru	-0.097135	-0.130895	-0.467618
Cl	-0.923584	-2.377924	-0.786020
C	-1.952942	0.156911	0.310164
C	-2.256253	1.009526	-0.656671
H	0.319012	-0.483146	-1.987120
H	-1.400027	1.501699	-1.177679
C	-3.587652	1.273236	-1.282277
C	-2.778536	-0.564421	1.287855
C	1.085908	-0.473302	1.284207
C	0.688511	0.900018	1.373233
C	1.194956	1.587986	0.240010
C	1.956245	0.664620	-0.541881
C	1.897378	-0.616032	0.095072
C	0.832542	-1.550899	2.273121
H	0.585132	-2.485797	1.767589
H	-0.003182	-1.304103	2.929452
H	1.713990	-1.723272	2.899033
C	-0.070152	1.541201	2.478173
H	-0.543665	0.803472	3.125800
H	-0.849708	2.206417	2.101371
H	0.602373	2.141787	3.098362
C	1.006303	3.034324	-0.036527
H	1.149829	3.262568	-1.093509
H	1.719186	3.640358	0.532166
H	0.004317	3.365352	0.245776
C	2.749859	1.017838	-1.748563
H	2.952230	0.140551	-2.362186
H	3.709689	1.459316	-1.462507
H	2.225321	1.740550	-2.374968
C	2.601508	-1.859878	-0.305619
H	1.942811	-2.724313	-0.208587
H	3.484983	-2.027267	0.318731
H	2.924539	-1.815075	-1.346028
H	-2.526110	-0.241708	2.303662
H	-2.587153	-1.637447	1.220835
H	-3.848215	-0.379337	1.145078
H	-3.801070	2.343339	-1.340718
H	-4.387600	0.793164	-0.717084
H	-3.617861	0.880185	-2.301706

E1

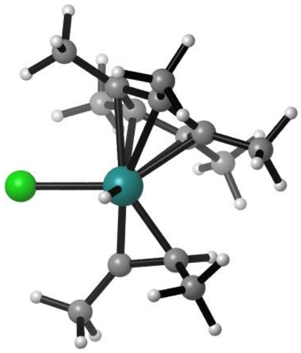

Ru	-0.271812	0.041215	-0.248875
Cl	-0.196080	0.493840	-2.609711
C	1.518443	0.564830	-0.214298
C	1.402681	-0.032717	1.049625
H	0.175560	-1.421938	-0.708542
H	1.228775	0.627304	1.903554
C	2.134389	-1.300354	1.397637
C	2.633147	0.955815	-1.075204
C	-2.201017	1.197932	-0.033399
C	-1.614207	1.046632	1.245546
C	-1.563952	-0.361952	1.537444
C	-2.201146	-1.061578	0.468885
C	-2.570013	-0.118561	-0.506477
C	-2.495604	2.454840	-0.765695
H	-2.135242	2.390905	-1.795574
H	-2.017971	3.316034	-0.297771
H	-3.573299	2.643987	-0.797932
C	-1.178571	2.124853	2.168402
H	-1.115720	3.085837	1.657866
H	-0.199026	1.919610	2.608861
H	-1.881650	2.232102	3.000294
C	-1.164351	-0.968400	2.832389
H	-0.729673	-1.960298	2.696030
H	-2.037558	-1.082459	3.483324
H	-0.436952	-0.355837	3.366087
C	-2.428602	-2.529615	0.428058
H	-2.519055	-2.893041	-0.595466
H	-3.346538	-2.795686	0.961622
H	-1.605707	-3.071552	0.896855
C	-3.294787	-0.378025	-1.773162
H	-2.788321	0.097699	-2.616110
H	-4.312050	0.021598	-1.715441
H	-3.364357	-1.444729	-1.986361
H	2.325326	1.136086	-2.103286
H	3.405746	0.177481	-1.048442
H	3.097359	1.861138	-0.668715
H	2.408559	-1.851566	0.496055
H	1.522552	-1.963791	2.015279
H	3.048515	-1.084920	1.961449

TS_{E1-E2}

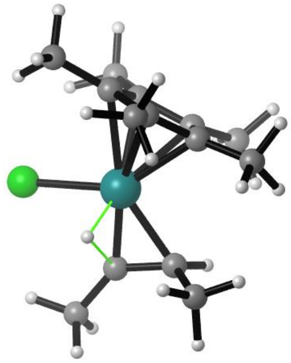

Ru	-0.368492	-0.179788	0.236650
Cl	-0.218294	-2.316653	1.337415
C	-2.224419	-0.461999	0.287287
C	-2.182324	0.837737	-0.249789
H	-1.142669	-1.101071	-0.901549
H	-2.436220	1.639294	0.450533
C	-2.601548	1.118515	-1.669623
C	-3.264113	-1.447422	0.584243
C	1.936998	-0.212591	0.207085
C	1.439640	0.494335	1.368808
C	0.730456	1.629889	0.914995
C	0.743053	1.601228	-0.523338
C	1.513099	0.475706	-0.942413
C	2.803511	-1.413793	0.263934
H	2.992356	-1.821718	-0.729158
H	2.343734	-2.199151	0.868706
H	3.769286	-1.162073	0.712990
C	1.738416	0.112352	2.770604
H	1.539455	-0.949251	2.932431
H	1.131770	0.676592	3.479350
H	2.791365	0.299879	3.004310
C	0.117153	2.696408	1.744240
H	-0.858186	2.999141	1.354672
H	0.747769	3.591121	1.762436
H	-0.025948	2.367802	2.773828
C	0.291898	2.700223	-1.412603
H	-0.044740	2.330174	-2.382042
H	1.121840	3.390361	-1.598406
H	-0.520301	3.277959	-0.969997
C	1.793696	0.119383	-2.357060
H	2.014519	-0.942368	-2.466259
H	2.650005	0.682369	-2.741705
H	0.938104	0.344574	-2.996714
H	-2.848702	-2.413483	0.865019
H	-3.945668	-1.557640	-0.267365
H	-3.863309	-1.066940	1.418237
H	-2.194822	0.378222	-2.361663
H	-2.286891	2.105968	-2.010198
H	-3.694987	1.088488	-1.753862

E2

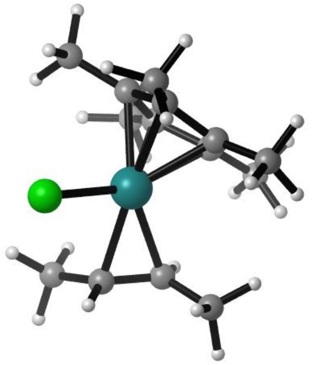

Ru	-0.255113	-0.187359	0.260486
Cl	-0.419223	-2.404209	1.063219
C	-2.463385	-0.420553	0.105735
C	-2.088460	0.565411	-0.787561
H	-2.636222	-1.422261	-0.282253
H	-2.234948	1.602806	-0.488507
C	-2.032202	0.304730	-2.261505
C	-3.059281	-0.132862	1.448894
C	1.898016	-0.187418	0.155213
C	1.476766	0.366598	1.388859
C	0.668213	1.532378	1.104745
C	0.658697	1.731482	-0.306701
C	1.351932	0.633766	-0.899717
C	2.741544	-1.387577	-0.062950
H	2.361942	-1.984300	-0.894758
H	2.753608	-2.034232	0.813506
H	3.772731	-1.102535	-0.295240
C	1.761221	-0.149257	2.750688
H	2.087682	-1.187985	2.724994
H	0.868356	-0.109574	3.378008
H	2.540505	0.446935	3.235921
C	0.058558	2.420454	2.124546
H	-0.824839	2.929294	1.734263
H	0.766203	3.188386	2.455460
H	-0.248052	1.853732	3.005462
C	0.105082	2.913055	-1.014621
H	-0.266766	2.665708	-2.009856
H	0.881064	3.674782	-1.138166
H	-0.713097	3.374374	-0.459695
C	1.611927	0.415938	-2.344643
H	1.423525	-0.622354	-2.626281
H	2.657661	0.635164	-2.584411
H	0.987330	1.051366	-2.972563
H	-2.735367	-0.861550	2.194041
H	-4.153276	-0.179293	1.398439
H	-2.785749	0.866141	1.799617
H	-1.763494	-0.735227	-2.463819
H	-1.313630	0.942743	-2.779208
H	-3.010538	0.488746	-2.719662

TS$_{E1\text{-}C1}$

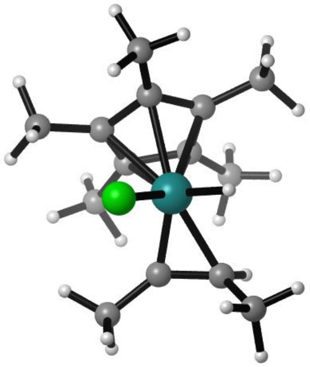

Ru	-0.355473	-0.434415	-0.204525
Cl	0.483829	-2.375233	-1.344897
C	1.292720	0.402862	-0.321471
C	1.211260	0.320847	1.070044
H	-0.244611	-1.427773	1.038652
H	0.878007	1.200341	1.627032
C	2.160299	-0.575491	1.823289
C	2.250077	0.727857	-1.369830
C	-1.837865	0.043012	-1.903427
C	-1.501394	1.257712	-1.283796
C	-1.877131	1.168258	0.104027
C	-2.549515	-0.077521	0.304246
C	-2.477371	-0.797755	-0.911302
C	-1.636072	-0.346729	-3.321049
H	-1.095294	-1.295185	-3.387030
H	-1.064310	0.403733	-3.868632
H	-2.595649	-0.472338	-3.832135
C	-0.883087	2.451199	-1.918981
H	-0.351149	2.193167	-2.835744
H	-0.172076	2.942709	-1.250533
H	-1.645078	3.192580	-2.178850
C	-1.767037	2.268777	1.096326
H	-1.735932	1.884472	2.117259
H	-2.619819	2.951864	1.025983
H	-0.864417	2.864454	0.940514
C	-3.213792	-0.506835	1.562889
H	-3.268692	-1.593128	1.635432
H	-4.234742	-0.115941	1.617363
H	-2.674789	-0.147211	2.440514
C	-3.034866	-2.145062	-1.197916
H	-2.302512	-2.767266	-1.717365
H	-3.925456	-2.071561	-1.830244
H	-3.317272	-2.665145	-0.282092
H	1.808522	0.681182	-2.366902
H	3.014634	-0.061844	-1.323158
H	2.759119	1.682714	-1.210308
H	2.434791	-1.439832	1.214543
H	1.703196	-0.950435	2.740738
H	3.074994	-0.039790	2.099997

C1

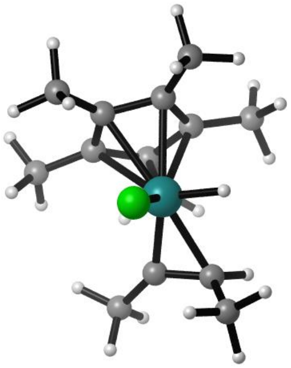

Ru	-0.382869	-0.329275	0.084149
Cl	-0.196930	-2.640475	0.656716
C	1.301582	0.144078	-0.568348
C	1.528292	0.330149	0.798890
H	-0.343111	-0.257806	1.660914
H	1.536105	1.364730	1.162362
C	2.377915	-0.602255	1.619264
C	1.989332	0.500498	-1.810037
C	-1.671560	0.074874	-1.841165
C	-1.396282	1.269229	-1.102933
C	-2.000403	1.152903	0.193415
C	-2.628421	-0.129334	0.254810
C	-2.412408	-0.782801	-0.996116
C	-1.315041	-0.205234	-3.258605
H	-0.869097	-1.195542	-3.373267
H	-0.604572	0.524767	-3.648545
H	-2.199064	-0.167903	-3.902743
C	-0.758782	2.511435	-1.616530
H	-0.133751	2.323181	-2.490086
H	-0.133230	2.983821	-0.855396
H	-1.519941	3.241252	-1.909767
C	-2.072628	2.238331	1.206712
H	-2.277861	1.840032	2.200777
H	-2.864300	2.954000	0.961779
H	-1.133999	2.793086	1.264544
C	-3.415596	-0.702709	1.378644
H	-3.121620	-1.736785	1.571991
H	-4.487086	-0.697166	1.153466
H	-3.265240	-0.141028	2.301213
C	-2.926173	-2.130041	-1.341400
H	-2.605834	-2.440901	-2.335815
H	-4.019998	-2.137701	-1.318491
H	-2.568019	-2.876156	-0.627096
H	1.428104	0.251994	-2.711799
H	2.941442	-0.041324	-1.842807
H	2.248166	1.566750	-1.821460
H	2.337189	-1.618658	1.228303
H	2.042007	-0.637238	2.657214
H	3.419724	-0.263537	1.615888

TS_{C1-C2}

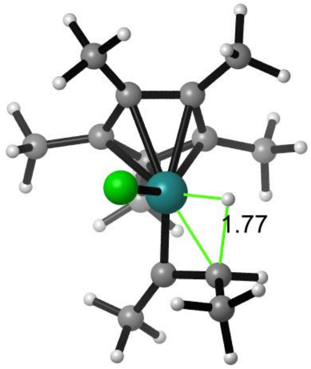

C	-1.666469	0.508786	-0.392428
C	-1.272165	0.802026	0.936024
C	-1.100563	-0.442252	1.643097
C	-1.495034	-1.501462	0.756357
C	-1.804462	-0.917322	-0.487909
Ru	0.398810	-0.400435	0.055485
C	2.387028	-0.189478	0.866459
C	3.597746	-0.716113	0.139432
C	-1.173339	2.150866	1.551564
C	-0.775026	-0.586135	3.086757
C	-1.552351	-2.951391	1.085199
C	-2.255557	-1.627515	-1.709058
C	-1.984552	1.465781	-1.483816
Cl	1.037297	-1.387875	-2.025576
C	1.668815	0.914216	0.359671
C	1.896665	2.358419	0.293145
H	1.320763	-1.578364	0.626345
H	2.454810	-0.288088	1.955027
H	-0.950101	2.923230	0.814331
H	-0.402391	2.192310	2.322983
H	-2.122478	2.419430	2.025362
H	-0.139341	0.230661	3.434829
H	-0.241508	-1.518709	3.280009
H	-1.678307	-0.586982	3.705583
H	-1.215142	-3.561363	0.245202
H	-2.570878	-3.264026	1.336480
H	-0.914686	-3.191577	1.937066
H	-1.720356	-1.275176	-2.592026
H	-3.325477	-1.456373	-1.866202
H	-2.089408	-2.702005	-1.636780
H	-3.065548	1.577064	-1.621898
H	-1.565611	1.125621	-2.433323
H	-1.574930	2.456919	-1.281115
H	1.076409	2.900535	-0.180376
H	2.803119	2.531674	-0.299540
H	2.091943	2.786548	1.283731
H	3.469801	-0.637262	-0.940070
H	3.786863	-1.764916	0.374600
H	4.480505	-0.139589	0.435023

C2

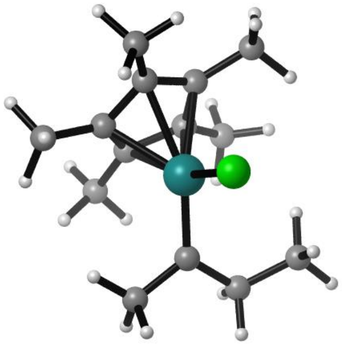

Ru	3.514607	1.297004	-0.237802
Cl	4.398949	2.217893	1.716821
C	1.668810	1.204899	0.171458
C	1.008237	0.061643	0.872470
H	0.494500	-0.526272	0.092975
H	0.187829	0.466511	1.484022
C	1.892241	-0.831243	1.717762
C	0.686349	2.221371	-0.297456
C	4.111976	1.952207	-2.197732
C	3.316459	0.772244	-2.335702
C	3.978198	-0.267097	-1.619574
C	5.258869	0.254773	-1.157535
C	5.350337	1.594646	-1.543591
C	3.822525	3.289186	-2.769941
H	4.156518	4.082503	-2.099028
H	2.756445	3.429777	-2.952239
H	4.343606	3.422832	-3.724414
C	2.046963	0.647287	-3.097212
H	1.488581	1.584790	-3.112220
H	1.395410	-0.116211	-2.666858
H	2.241382	0.366017	-4.136924
C	3.570047	-1.692534	-1.552282
H	3.948652	-2.174670	-0.649590
H	3.963150	-2.250792	-2.409153
H	2.484037	-1.802510	-1.558169
C	6.230402	-0.532616	-0.359495
H	6.989942	0.105614	0.090994
H	6.734177	-1.284140	-0.975544
H	5.723657	-1.055945	0.455703
C	6.427038	2.566129	-1.225928
H	6.023492	3.448683	-0.723556
H	6.934589	2.899561	-2.136269
H	7.173942	2.135060	-0.560106
H	1.109865	2.952932	-0.985967
H	0.323647	2.771600	0.579847
H	-0.198427	1.755944	-0.754988
H	2.665842	-1.311221	1.114437
H	1.308358	-1.614790	2.205199
H	2.400475	-0.249872	2.489173

TS_{C2-Z1}

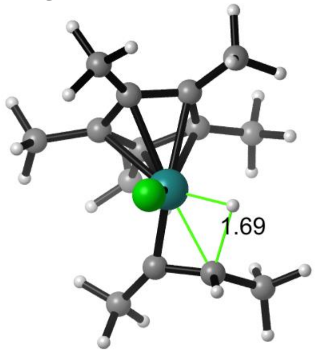

Ru	0.184402	-0.322969	-0.265127
Cl	-0.534929	-1.125770	-2.392682
C	1.811164	0.460568	-0.711105
C	2.226563	-0.887999	-0.667456
H	0.851931	-1.785056	-0.283220
C	3.193131	-1.357599	0.387545
H	2.304096	-1.406605	-1.627166
C	2.534970	1.732858	-0.741186
C	-0.274274	1.012769	1.456309
C	-0.396715	-0.366939	1.847124
C	-1.513250	-0.923627	1.141504
C	-2.018039	0.086302	0.285808
C	-1.268844	1.286957	0.477582
C	0.648316	1.999665	2.077143
H	0.856066	2.838363	1.411063
H	1.604541	1.547309	2.348361
H	0.210962	2.412397	2.991456
C	0.372367	-1.040821	2.926837
H	1.351180	-0.580209	3.069897
H	0.537868	-2.095276	2.697641
H	-0.158221	-0.988566	3.883013
C	-2.047234	-2.306340	1.271451
H	-2.287054	-2.731148	0.294519
H	-2.958091	-2.330800	1.878263
H	-1.319707	-2.968599	1.742793
C	-3.170398	-0.040957	-0.639818
H	-2.937350	0.372509	-1.622371
H	-4.034552	0.498469	-0.238837
H	-3.455747	-1.081900	-0.787392
C	-1.552447	2.585469	-0.187106
H	-2.396580	3.101753	0.282774
H	-1.802436	2.439312	-1.239758
H	-0.693090	3.256955	-0.143823
H	1.890305	2.597848	-0.574509
H	3.006475	1.848018	-1.723581
H	3.349305	1.742447	-0.004565
H	3.051684	-2.413282	0.630483
H	3.069531	-0.783283	1.310157
H	4.225508	-1.230891	0.046954

Z1

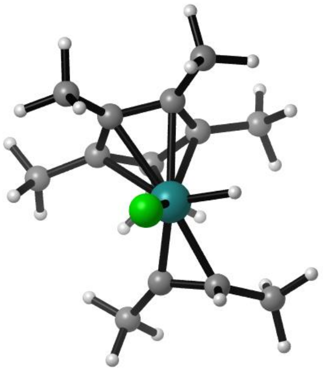

Ru	0.768616	0.926364	1.310070
Cl	-0.653050	0.791738	-0.603920
C	2.363840	0.253210	0.578447
C	1.675622	-0.941373	0.775576
H	-0.191862	-0.264118	1.708974
H	1.079639	-1.334963	-0.049582
C	2.133953	-1.952603	1.788991
C	3.769356	0.600934	0.361792
C	-0.003356	3.065678	1.783427
C	1.409377	3.086308	1.899158
C	1.776086	2.133076	2.898921
C	0.577984	1.530503	3.407893
C	-0.527197	2.121634	2.712488
C	-0.825846	3.897576	0.871001
H	-1.398870	3.267748	0.184281
H	-0.209441	4.567762	0.271903
H	-1.532569	4.506126	1.442377
C	2.322278	3.989291	1.147705
H	2.071356	4.017476	0.085638
H	3.363425	3.675418	1.233403
H	2.263510	5.014242	1.527269
C	3.135020	1.911630	3.461214
H	3.283432	0.869666	3.751839
H	3.284093	2.523442	4.356548
H	3.922584	2.174288	2.754051
C	0.504054	0.594060	4.560417
H	-0.418404	0.012714	4.539002
H	0.536500	1.135764	5.511443
H	1.334972	-0.113474	4.554888
C	-1.976411	1.866476	2.927176
H	-2.499597	1.763144	1.974196
H	-2.444357	2.690159	3.476364
H	-2.143392	0.950019	3.494049
H	4.416349	0.027940	1.039029
H	3.986589	1.664365	0.470695
H	4.049670	0.300158	-0.653580
H	1.283136	-2.439493	2.272041
H	2.733616	-1.480152	2.573201
H	2.743544	-2.736227	1.326406

$TS_{Z1\text{-}Z2}$

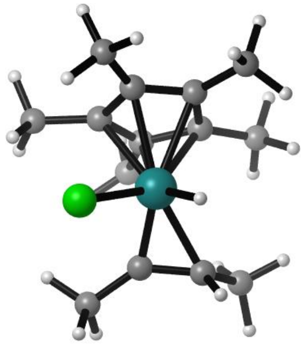

Ru	-0.380541	-0.063368	-0.558516
Cl	-0.607627	-2.408914	-1.043212
C	-2.058434	-0.176382	0.210226
C	-2.310184	0.926787	-0.613346
H	-0.783030	0.034517	-2.101354
C	-2.550946	2.301400	-0.050021
H	-2.879017	0.729688	-1.521306
C	-2.746053	-1.022849	1.176384
C	1.892388	-0.391322	-0.505413
C	1.371451	-0.452466	0.846339
C	0.841881	0.816578	1.155115
C	0.978368	1.649828	-0.012361
C	1.683508	0.907212	-1.007478
C	2.620192	-1.501138	-1.168470
H	2.765223	-1.309769	-2.231923
H	2.073102	-2.440956	-1.073642
H	3.606288	-1.632973	-0.711627
C	1.473409	-1.639093	1.732695
H	1.075182	-2.528187	1.235352
H	0.917026	-1.495062	2.660262
H	2.514952	-1.845439	1.999515
C	0.289652	1.240099	2.470636
H	-0.416070	2.065727	2.368696
H	1.088526	1.578443	3.137811
H	-0.230048	0.421391	2.971952
C	0.703213	3.108015	-0.098481
H	0.330100	3.387281	-1.085846
H	1.620428	3.679701	0.077511
H	-0.031094	3.435036	0.637293
C	2.124317	1.446969	-2.320336
H	2.269299	0.652624	-3.052572
H	3.071551	1.987199	-2.221375
H	1.390613	2.142069	-2.731041
H	-2.055457	-1.653299	1.738879
H	-3.412684	-1.688029	0.612528
H	-3.375560	-0.432645	1.850710
H	-2.142176	3.084382	-0.692576
H	-2.107544	2.415718	0.941965
H	-3.625584	2.491104	0.044998

Z2

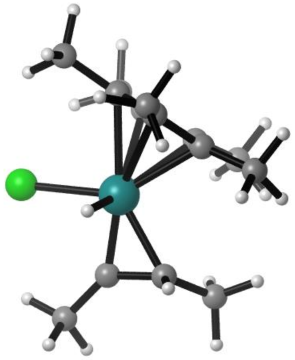

Ru	-0.335768	-0.264955	-0.276593
Cl	-0.184754	-2.655262	-0.017896
C	-2.173235	-0.555370	-0.353907
C	-2.138566	0.832765	-0.556107
H	-0.488821	-0.569774	-1.831087
C	-2.745085	1.735232	0.485076
H	-2.243172	1.204599	-1.578327
C	-3.201486	-1.592808	-0.352816
C	1.970986	-0.284898	-0.181973
C	1.443355	-0.052847	1.146180
C	0.746652	1.170645	1.129293
C	0.792258	1.680336	-0.219295
C	1.598605	0.799448	-0.997620
C	2.849736	-1.420294	-0.549893
H	2.994879	-1.483620	-1.628631
H	2.422116	-2.367724	-0.215615
H	3.833382	-1.305449	-0.083482
C	1.700562	-0.932181	2.312604
H	1.411114	-1.962845	2.090302
H	1.144661	-0.605998	3.192017
H	2.764778	-0.930758	2.568648
C	0.162872	1.858043	2.311504
H	-0.597921	2.584625	2.029147
H	0.941009	2.396515	2.861971
H	-0.297000	1.150085	3.003036
C	0.330270	3.013073	-0.685875
H	-0.132805	2.954578	-1.672852
H	1.177168	3.702757	-0.762402
H	-0.393403	3.459359	-0.004850
C	1.971352	1.022951	-2.418353
H	2.206891	0.086732	-2.924451
H	2.849231	1.673072	-2.489290
H	1.162010	1.503053	-2.970483
H	-2.821308	-2.562938	-0.039310
H	-3.636228	-1.682972	-1.354938
H	-4.017179	-1.276260	0.308133
H	-2.361804	2.756721	0.434043
H	-2.573643	1.352969	1.493798
H	-3.829822	1.798674	0.337397

TS$_{Z2-Z3}$

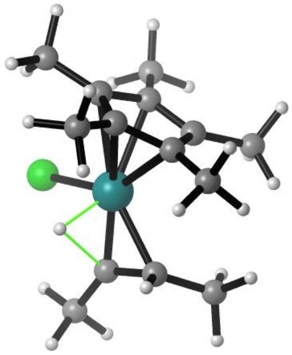

Ru	-0.354161	-0.097101	-0.187275
Cl	-1.090814	-2.366754	0.179861
C	-2.165968	0.273203	-0.496857
C	-1.654196	1.578658	-0.479427
H	-0.906028	-0.321277	-1.700941
C	-2.089321	2.528500	0.602259
H	-1.422926	2.059572	-1.432329
C	-3.482832	-0.346264	-0.629957
C	1.784365	-0.921156	-0.091516
C	1.370386	-0.485868	1.224507
C	1.134682	0.902440	1.170002
C	1.357857	1.332231	-0.188928
C	1.799269	0.203285	-0.939941
C	2.204894	-2.302854	-0.425269
H	2.344508	-2.433611	-1.498600
H	1.457139	-3.027056	-0.094576
H	3.152166	-2.541941	0.068358
C	1.310380	-1.363110	2.418703
H	0.706616	-2.251278	2.216727
H	0.875066	-0.846499	3.274529
H	2.314517	-1.694676	2.701649
C	0.800755	1.773845	2.326688
H	0.346406	2.712926	2.012844
H	1.703633	2.019994	2.894629
H	0.105715	1.283569	3.010441
C	1.363833	2.731739	-0.684389
H	1.021349	2.791540	-1.719261
H	2.376594	3.146360	-0.651605
H	0.724414	3.381650	-0.086476
C	2.192070	0.231068	-2.372401
H	2.051337	-0.741365	-2.844515
H	3.245445	0.507548	-2.480999
H	1.601774	0.958058	-2.932232
H	-3.459290	-1.418861	-0.447372
H	-3.903259	-0.146428	-1.621651
H	-4.154098	0.132876	0.092686
H	-1.378069	3.346180	0.743500
H	-2.209573	2.015514	1.559102
H	-3.050596	2.988801	0.345231

Z3

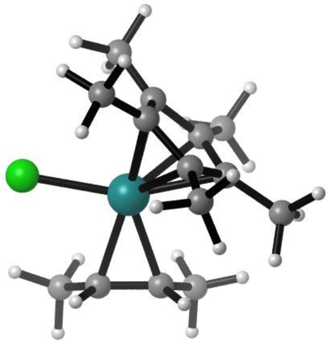

Ru	-0.141536	-0.297954	-0.165840
Cl	-0.227137	-2.656859	0.017377
C	-2.190946	-0.604504	-0.969887
C	-1.969523	0.762511	-0.899376
H	-1.942086	-1.103235	-1.905864
C	-2.706773	1.638975	0.068837
H	-1.622016	1.256599	-1.805390
C	-3.165784	-1.337411	-0.100631
C	2.011036	-0.271678	-0.124171
C	1.509902	-0.077542	1.186731
C	0.712350	1.129116	1.182269
C	0.784929	1.706300	-0.119378
C	1.512446	0.803355	-0.947638
C	2.876336	-1.375295	-0.606926
H	2.565858	-1.711338	-1.598021
H	2.826373	-2.241141	0.051666
H	3.918660	-1.047621	-0.671944
C	1.736706	-0.932725	2.377066
H	2.060736	-1.933445	2.094588
H	0.818777	-1.044815	2.957604
H	2.496991	-0.494106	3.031340
C	0.063235	1.708911	2.383719
H	-0.686908	2.455282	2.121278
H	0.803763	2.189522	3.031829
H	-0.431751	0.933963	2.972694
C	0.313585	3.050221	-0.541123
H	-0.264901	3.021856	-1.467037
H	1.174088	3.701865	-0.721317
H	-0.298975	3.527179	0.223478
C	1.820765	1.006588	-2.383684
H	1.922213	0.054203	-2.905673
H	2.762004	1.554018	-2.506382
H	1.037521	1.580710	-2.881499
H	-2.940614	-2.402363	-0.077294
H	-4.183108	-1.208441	-0.488452
H	-3.161405	-0.978115	0.930103
H	-2.271009	2.635995	0.138541
H	-2.735031	1.213964	1.073721
H	-3.746871	1.764756	-0.254412

TS$_{A3\text{-}Z1}$

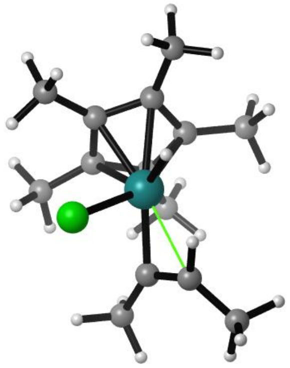

Ru	1.136561	0.413449	-1.828358
Cl	3.358924	-0.240329	-2.553361
C	2.162369	0.962564	-0.166814
C	1.929376	-0.226005	0.353130
H	0.879059	-1.086299	-2.337665
H	1.702569	-1.045474	-0.380363
C	1.837040	-0.647250	1.783327
C	2.767560	2.187134	0.367004
C	0.854646	2.297732	-2.832018
C	-0.134008	2.208962	-1.801129
C	-0.970439	1.080960	-2.089024
C	-0.547797	0.516108	-3.324106
C	0.606541	1.224802	-3.766501
C	1.918450	3.322742	-2.995214
H	2.883875	2.848048	-3.185873
H	2.024767	3.937115	-2.100214
H	1.699789	3.988789	-3.835576
C	-0.388487	3.163811	-0.689285
H	0.374453	3.940565	-0.640006
H	-0.417541	2.660618	0.280440
H	-1.352794	3.662061	-0.827605
C	-2.147155	0.662569	-1.285604
H	-2.357669	-0.400512	-1.409709
H	-3.044829	1.215644	-1.582481
H	-1.984795	0.847325	-0.222331
C	-1.221415	-0.590668	-4.051922
H	-0.518545	-1.149604	-4.669402
H	-1.998681	-0.189674	-4.710039
H	-1.696731	-1.294189	-3.367668
C	1.346759	0.994944	-5.031202
H	2.388851	1.297796	-4.935939
H	0.893293	1.562007	-5.850908
H	1.347203	-0.060983	-5.304155
H	2.106132	3.053565	0.300893
H	3.653177	2.416510	-0.236004
H	3.092025	2.072428	1.406303
H	0.817391	-0.942722	2.045086
H	2.128105	0.169839	2.446091
H	2.483090	-1.503898	1.989992

TS$_{C2\text{-}R1}$

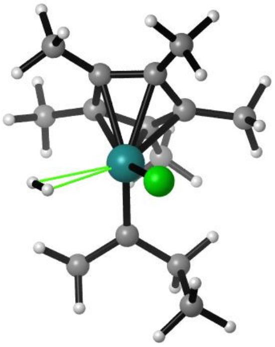

Ru	0.365520	-0.136435	0.098238
Cl	0.565414	-2.456074	0.675733
C	0.407704	0.627597	1.826232
C	-0.704696	0.486139	2.814897
H	-1.635929	0.215233	2.310070
H	-0.875517	1.425019	3.363961
C	-0.352901	-0.629158	3.799443
C	1.494341	1.525463	2.311637
C	0.233863	1.445394	-1.364022
C	-0.999824	1.347371	-0.660512
C	-1.521919	0.026366	-0.881695
C	-0.687959	-0.614798	-1.879709
C	0.392896	0.224690	-2.142636
C	1.110745	2.640875	-1.465305
H	2.161667	2.356581	-1.552715
H	1.016925	3.279634	-0.585337
H	0.863754	3.247583	-2.343639
C	-1.653292	2.427105	0.123221
H	-0.919803	3.085982	0.592505
H	-2.291708	2.027563	0.912310
H	-2.283222	3.043410	-0.525482
C	-2.804540	-0.531141	-0.387207
H	-2.687035	-1.582332	-0.117169
H	-3.581078	-0.468007	-1.157491
H	-3.167474	0.002250	0.492306
C	-0.934908	-1.979809	-2.406058
H	-0.155830	-2.289674	-3.102390
H	-1.892631	-2.021803	-2.933330
H	-0.960017	-2.712349	-1.594526
C	1.546152	-0.041615	-3.039722
H	2.485962	0.279355	-2.585893
H	1.441428	0.502718	-3.983872
H	1.639581	-1.102687	-3.270944
H	2.384114	1.540749	1.682631
H	1.790582	1.259761	3.334170
H	1.101202	2.550127	2.373697
H	-0.178267	-1.563731	3.262109
H	-1.166753	-0.779216	4.510221
H	0.547998	-0.391312	4.368857
H	2.771508	0.026069	0.086412
H	2.712023	-0.688998	0.330864

R1

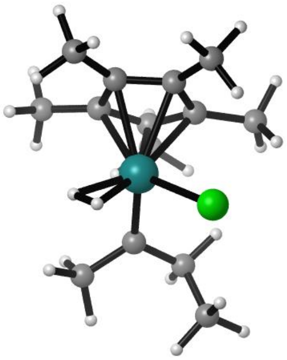

Ru	0.500184	-0.155630	0.135993
Cl	-0.060447	-2.469850	0.622205
C	0.250892	0.484177	1.897724
C	-1.072578	0.378033	2.584966
H	-1.862623	0.134855	1.870802
H	-1.332614	1.327095	3.078396
C	-1.013466	-0.737485	3.629981
C	1.250038	1.238654	2.708372
C	0.527571	1.547260	-1.235329
C	-0.807825	1.433344	-0.738676
C	-1.309154	0.167825	-1.130673
C	-0.329619	-0.442175	-2.017300
C	0.782347	0.394811	-2.081351
C	1.392634	2.754469	-1.148085
H	2.449801	2.492787	-1.218661
H	1.249118	3.283702	-0.204145
H	1.174961	3.460323	-1.957458
C	-1.531726	2.488076	0.016345
H	-0.865472	3.033898	0.687905
H	-2.346085	2.079706	0.615219
H	-1.965873	3.216722	-0.675077
C	-2.655535	-0.402364	-0.871902
H	-2.575559	-1.428907	-0.504191
H	-3.252920	-0.422352	-1.789085
H	-3.208427	0.178279	-0.132253
C	-0.533464	-1.745493	-2.695037
H	0.336528	-2.034512	-3.284734
H	-1.394282	-1.695319	-3.368823
H	-0.723210	-2.537349	-1.964876
C	2.029209	0.194286	-2.865224
H	2.915290	0.449925	-2.280210
H	2.036904	0.829932	-3.756491
H	2.138191	-0.839865	-3.191585
H	2.286448	1.043023	2.435194
H	1.122647	1.078327	3.784810
H	1.065906	2.309308	2.540378
H	-0.754539	-1.683927	3.151950
H	-1.984116	-0.848136	4.115183
H	-0.274981	-0.526584	4.405812
H	2.205744	0.022055	0.480366
H	2.076681	-0.810282	0.612036

$TS_{R1\text{-}R2}$

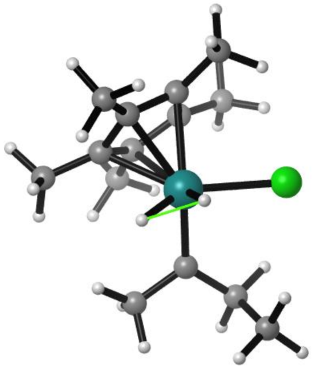

Ru	0.343643	0.062379	-0.206990
Cl	-0.395845	2.108155	-1.304511
C	0.166483	-0.910687	-1.857398
C	-0.440156	-0.412244	-3.127673
H	-1.194427	0.349245	-2.932196
H	-0.904547	-1.246893	-3.670537
C	0.656552	0.200728	-4.000697
C	0.553161	-2.343594	-1.993482
C	0.070934	-1.318490	1.488534
C	-1.261069	-1.035644	1.058893
C	-1.474027	0.348515	1.211971
C	-0.301685	0.937518	1.822370
C	0.639182	-0.088543	2.009308
C	0.649652	-2.676549	1.668668
H	1.739931	-2.656569	1.641361
H	0.312086	-3.367450	0.894332
H	0.350360	-3.098246	2.633826
C	-2.236655	-2.030164	0.540343
H	-1.733568	-2.883477	0.081826
H	-2.896324	-1.591846	-0.210425
H	-2.865789	-2.420424	1.346438
C	-2.715388	1.096311	0.903223
H	-2.496494	1.973161	0.288990
H	-3.186754	1.440874	1.828957
H	-3.437853	0.480170	0.367468
C	-0.189083	2.367720	2.202431
H	0.818308	2.613425	2.539128
H	-0.882081	2.614307	3.013042
H	-0.423717	3.011947	1.350909
C	1.960206	0.020306	2.682823
H	2.691542	-0.659087	2.243285
H	1.874968	-0.227714	3.745480
H	2.364705	1.029799	2.606364
H	1.184079	-2.723209	-1.192186
H	1.038513	-2.530786	-2.959382
H	-0.373859	-2.934295	-2.017891
H	1.080153	1.075694	-3.507209
H	0.240194	0.511364	-4.959851
H	1.460643	-0.512732	-4.198001
H	1.680923	-0.773907	-0.228289
H	1.730492	0.796349	-0.521874

R2

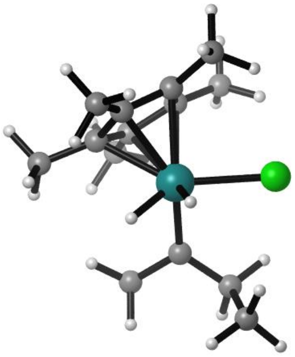

Ru	0.246622	0.007445	-0.462823
C	1.922009	0.664501	0.227671
C	3.145489	-0.124064	0.560587
H	2.880069	-1.082013	1.007395
H	3.773434	0.436880	1.265350
C	3.942030	-0.389609	-0.716975
C	2.149768	2.113487	0.484909
H	2.275472	2.256541	1.566720
H	1.356652	2.769227	0.129848
H	3.101651	2.430517	0.038956
H	4.163693	0.537833	-1.252102
H	3.376620	-1.044232	-1.380062
H	4.889532	-0.872838	-0.474378
C	-1.862953	0.453377	-1.122764
C	-1.406181	1.466741	-0.193590
C	-1.256865	0.855335	1.084795
C	-1.515312	-0.524125	0.929948
C	-1.921779	-0.770163	-0.437759
C	-2.258507	0.713917	-2.532128
H	-2.187562	-0.188249	-3.139923
H	-1.621171	1.469701	-2.993192
H	-3.290519	1.075055	-2.585457
C	-1.408712	2.929750	-0.460958
H	-0.737180	3.468471	0.209261
H	-2.411997	3.342482	-0.312099
H	-1.109098	3.155184	-1.485162
C	-0.903924	1.557015	2.346397
H	-0.362989	0.903297	3.031971
H	-1.802977	1.907818	2.862800
H	-0.277718	2.431117	2.159061
C	-1.495886	-1.560822	1.989901
H	-0.878693	-2.411161	1.687667
H	-2.509309	-1.928943	2.177379
H	-1.100933	-1.172750	2.929012
C	-2.359803	-2.089926	-0.954462
H	-3.305640	-2.394498	-0.495405
H	-1.613998	-2.857407	-0.732305
H	-2.505195	-2.068210	-2.034705
Cl	1.020342	-2.274908	-0.142242
H	0.759789	-0.384951	-1.924648
H	0.618132	1.166781	-1.472991

$TS_{R2\text{-}R3}$

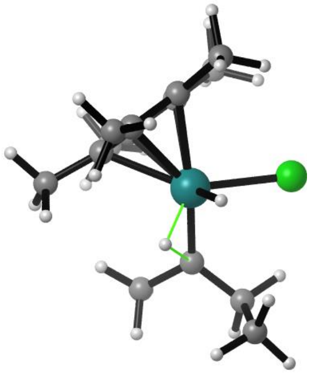

Ru	0.090255	0.195514	-0.262610
C	1.072225	1.814438	0.189512
C	2.557211	1.972549	0.152032
H	3.030514	1.106116	0.618053
H	2.848209	2.874659	0.705029
C	3.056032	2.085232	-1.285948
C	0.402775	3.099481	0.529324
H	0.623565	3.318478	1.581822
H	-0.676413	3.097231	0.391234
H	0.836932	3.929734	-0.043587
H	2.538246	2.881923	-1.828492
H	2.890400	1.147165	-1.816067
H	4.123534	2.308523	-1.298924
C	-1.851842	-0.503015	-1.047091
C	-2.107400	0.594578	-0.148228
C	-1.751355	0.177268	1.164814
C	-1.233101	-1.140270	1.073386
C	-1.313494	-1.576399	-0.291069
C	-2.223491	-0.523752	-2.487059
H	-1.625170	-1.245595	-3.043552
H	-2.074817	0.452098	-2.951610
H	-3.277332	-0.792995	-2.612276
C	-2.852115	1.828568	-0.519386
H	-2.780776	2.598566	0.249952
H	-3.915880	1.606640	-0.653540
H	-2.488905	2.256441	-1.455587
C	-1.914944	0.965342	2.415076
H	-1.109560	0.762898	3.122756
H	-2.859262	0.721947	2.912343
H	-1.919725	2.038999	2.220217
C	-0.753885	-1.985876	2.194175
H	0.257055	-2.353107	1.997683
H	-1.409600	-2.852630	2.321328
H	-0.735242	-1.437747	3.136259
C	-0.934953	-2.931566	-0.766653
H	-1.649678	-3.686147	-0.422820
H	0.053806	-3.209018	-0.393501
H	-0.900230	-2.976164	-1.855738
Cl	2.000399	-1.139696	0.394596
H	0.754893	-0.248405	-1.633378
H	0.217939	1.399705	-1.325465

R3

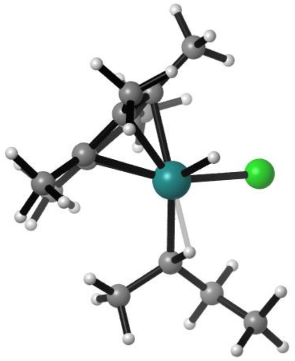

Ru	0.133337	-0.108771	-0.504750
C	1.907593	0.643042	0.161411
C	3.150043	-0.125757	0.544532
H	2.887305	-1.131152	0.878494
H	3.627460	0.392102	1.384884
C	4.134386	-0.237663	-0.609051
C	1.999743	2.075406	0.608778
H	1.950794	2.128243	1.700979
H	1.220872	2.713160	0.190256
H	2.970916	2.505232	0.327728
H	4.397533	0.746828	-1.008898
H	3.696463	-0.829883	-1.415850
H	5.057789	-0.727997	-0.296035
C	-1.904725	0.475785	-1.139646
C	-1.462675	1.495172	-0.236048
C	-1.210841	0.885752	1.018444
C	-1.459033	-0.519999	0.890886
C	-1.933744	-0.770345	-0.442887
C	-2.374714	0.738216	-2.525300
H	-2.391663	-0.172518	-3.123488
H	-1.729224	1.455388	-3.034738
H	-3.387292	1.154631	-2.514803
C	-1.411456	2.943004	-0.566390
H	-0.918827	3.524360	0.213302
H	-2.421870	3.346745	-0.687915
H	-0.878728	3.120279	-1.503385
C	-0.838138	1.561473	2.287362
H	-0.120407	0.970804	2.859279
H	-1.724501	1.694522	2.915574
H	-0.403527	2.546873	2.120860
C	-1.378990	-1.526594	1.978198
H	-1.136888	-2.512018	1.581005
H	-2.334455	-1.595635	2.509113
H	-0.607155	-1.269560	2.705155
C	-2.401454	-2.085713	-0.950081
H	-3.354224	-2.368161	-0.491910
H	-1.670364	-2.867220	-0.732460
H	-2.540799	-2.065146	-2.031510
Cl	1.125461	-2.302657	-0.451899
H	0.004057	-0.657638	-1.990937
H	1.771545	0.627509	-1.017262

$TS_{R3\text{-}D1}$

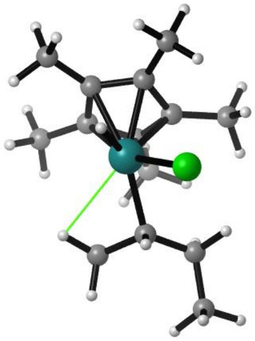

Ru	0.001457	0.000140	-1.029809
C	1.980102	0.389243	-0.354869
C	2.581372	-0.195908	0.893664
H	2.327705	-1.260812	0.929591
H	2.142114	0.273746	1.782069
C	4.093380	-0.038456	0.952878
C	2.093641	1.887039	-0.509332
H	1.804000	2.424924	0.398161
H	1.478370	2.265337	-1.339139
H	3.121327	2.185671	-0.755315
H	4.387680	1.014097	0.977901
H	4.560909	-0.493755	0.075965
H	4.510904	-0.517406	1.840987
C	-2.123283	0.593342	-1.023201
C	-1.470537	1.524164	-0.158023
C	-0.882829	0.810282	0.913273
C	-1.105608	-0.586846	0.689628
C	-1.921173	-0.722841	-0.500729
C	-2.969114	0.977523	-2.184385
H	-3.141815	0.133136	-2.851189
H	-2.504214	1.770171	-2.772634
H	-3.942962	1.345934	-1.845811
C	-1.496000	2.998642	-0.324841
H	-0.725262	3.488352	0.270674
H	-2.464639	3.405080	-0.015032
H	-1.347153	3.283943	-1.368364
C	-0.289808	1.419837	2.130373
H	0.287724	0.698517	2.706708
H	-1.088104	1.792862	2.779970
H	0.360355	2.265817	1.902996
C	-0.732778	-1.703465	1.592713
H	-0.518158	-2.607957	1.023179
H	-1.549467	-1.924267	2.288220
H	0.155361	-1.467104	2.180252
C	-2.504343	-1.995287	-0.993201
H	-3.325652	-2.322780	-0.347768
H	-1.750331	-2.783697	-1.017207
H	-2.890979	-1.887446	-2.007065
Cl	0.999846	-2.105599	-1.628919
H	-0.547403	-0.358487	-2.481722
H	2.444963	-0.105598	-1.219679

D1

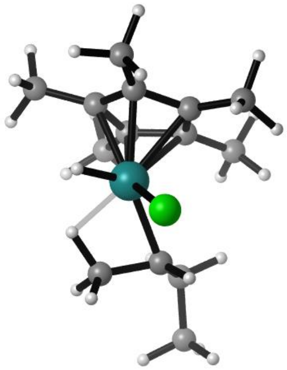

Ru	0.031068	-0.181934	0.018920
C	2.088958	0.376623	0.085002
C	2.791887	0.842942	1.329377
H	2.990145	-0.002698	1.992050
H	2.154581	1.541353	1.886926
C	4.115645	1.517475	0.998720
C	1.488820	1.429034	-0.750302
H	1.604433	2.437330	-0.344445
H	0.300316	1.390696	-0.825989
H	1.747554	1.376522	-1.807002
H	3.966417	2.401960	0.374082
H	4.768685	0.835919	0.448259
H	4.643489	1.832092	1.901577
C	-1.809676	0.054805	1.131222
C	-0.743340	0.271025	2.064260
C	-0.015637	-0.950080	2.189682
C	-0.571148	-1.894109	1.289311
C	-1.704549	-1.292616	0.645920
C	-2.899444	1.025528	0.847132
H	-3.388847	0.811650	-0.103250
H	-2.519015	2.047242	0.798500
H	-3.661812	0.995175	1.632539
C	-0.565032	1.509378	2.868899
H	0.395782	1.525033	3.383936
H	-1.348369	1.599248	3.628602
H	-0.617846	2.402694	2.242062
C	1.059433	-1.255378	3.166800
H	1.863475	-1.840376	2.716901
H	0.648090	-1.849152	3.989208
H	1.497293	-0.356725	3.599210
C	-0.128934	-3.296814	1.096035
H	-0.172389	-3.575980	0.042414
H	-0.768256	-3.983228	1.660739
H	0.899512	-3.441776	1.428158
C	-2.630069	-2.008424	-0.270658
H	-3.246244	-2.726902	0.279109
H	-2.074009	-2.554197	-1.034967
H	-3.297272	-1.315302	-0.784039
Cl	0.804944	-1.563317	-1.819878
H	-0.966940	0.126903	-1.185879
H	2.665696	-0.356300	-0.476400

TS$_{D1-D2}$

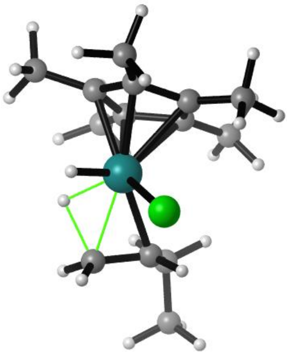

Ru	0.036612	-0.120164	-0.002737
C	2.168800	0.423151	0.050896
C	2.816757	0.930962	1.304722
H	2.951705	0.123465	2.027971
H	2.170596	1.679901	1.779254
C	4.172490	1.549956	0.997435
C	1.514200	1.345496	-0.796685
H	1.535553	2.396094	-0.504104
H	-0.008018	1.450145	-0.358748
H	1.517872	1.187382	-1.869414
H	4.069620	2.397693	0.315384
H	4.831077	0.822525	0.516871
H	4.668013	1.903966	1.903826
C	-1.792088	0.054066	1.116130
C	-0.714622	0.265245	2.045262
C	-0.010468	-0.973522	2.187098
C	-0.585307	-1.908772	1.303256
C	-1.690151	-1.287283	0.627453
C	-2.887801	1.018711	0.831724
H	-3.326078	0.846876	-0.151693
H	-2.530340	2.049094	0.856099
H	-3.685730	0.927149	1.575491
C	-0.538142	1.482044	2.883034
H	0.479504	1.568141	3.265752
H	-1.212709	1.465553	3.745425
H	-0.754135	2.390060	2.317158
C	1.068489	-1.274809	3.163084
H	1.887114	-1.834706	2.706601
H	0.673524	-1.885599	3.980850
H	1.484903	-0.370485	3.605528
C	-0.173066	-3.320350	1.111617
H	0.062484	-3.513597	0.061473
H	-0.981934	-3.995898	1.405757
H	0.707096	-3.568669	1.704715
C	-2.612530	-1.993475	-0.299129
H	-3.266965	-2.680661	0.246829
H	-2.051519	-2.573258	-1.034618
H	-3.243063	-1.291542	-0.845607
Cl	0.801280	-1.636661	-1.733539
H	-0.828009	0.266802	-1.274988
H	2.672361	-0.409734	-0.432695

D2

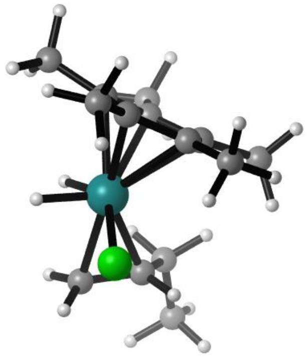

Ru	0.006558	0.561177	-0.272290
C	2.173059	0.071699	-0.477929
C	2.999011	-0.053067	0.767487
H	2.735316	-0.952796	1.330176
H	2.798980	0.803075	1.421917
C	4.479601	-0.099357	0.420776
C	1.952148	1.319466	-1.046724
H	2.308249	2.202250	-0.525473
H	0.481582	1.791399	0.591568
H	1.795268	1.433540	-2.110442
H	4.784312	0.815863	-0.092250
H	4.698805	-0.936784	-0.246275
H	5.098247	-0.211185	1.313470
C	-1.563121	0.689299	1.189549
C	-0.454206	-0.016622	1.798613
C	-0.352386	-1.300569	1.172249
C	-1.302217	-1.354483	0.143632
C	-2.048315	-0.117263	0.126399
C	-2.163532	1.958282	1.678714
H	-2.631561	2.514118	0.866062
H	-1.415885	2.609912	2.131634
H	-2.930462	1.754009	2.432277
C	0.239891	0.387353	3.050196
H	1.279655	0.058586	3.063695
H	-0.256225	-0.051368	3.922079
H	0.236332	1.470216	3.176910
C	0.544081	-2.417662	1.572094
H	1.137344	-2.788275	0.732640
H	-0.043055	-3.260007	1.949541
H	1.230388	-2.121838	2.365020
C	-1.566312	-2.505373	-0.748187
H	-1.407418	-2.224775	-1.793819
H	-2.606197	-2.828761	-0.644602
H	-0.919850	-3.352254	-0.518028
C	-3.208542	0.149212	-0.760557
H	-4.079206	-0.438720	-0.452087
H	-2.970905	-0.112226	-1.793668
H	-3.490136	1.202247	-0.741737
Cl	-0.197112	-0.131879	-2.590132
H	-0.408171	1.950381	-0.914358
H	2.173166	-0.793499	-1.140004

$TS_{D2\text{-}D3}$

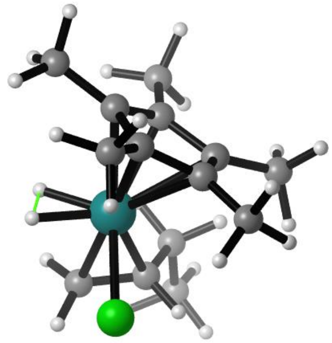

Ru	-0.031052	0.523758	-0.299690
C	2.184929	0.028976	-0.447081
C	2.990829	-0.131581	0.807214
H	2.740613	-1.055056	1.332114
H	2.766386	0.696731	1.488735
C	4.476775	-0.138213	0.478891
C	1.983594	1.292669	-0.965149
H	2.321144	2.147091	-0.385329
H	0.094099	2.115712	0.002480
H	1.842522	1.455451	-2.025550
H	4.773872	0.802983	0.010551
H	4.718769	-0.942219	-0.220600
H	5.084217	-0.279714	1.375073
C	-1.549315	0.681059	1.207685
C	-0.427888	0.001693	1.800754
C	-0.312426	-1.271208	1.143218
C	-1.294430	-1.340970	0.135267
C	-2.064919	-0.131377	0.151687
C	-2.139931	1.957337	1.691810
H	-2.611398	2.514621	0.881414
H	-1.385972	2.602625	2.144557
H	-2.905200	1.758849	2.448164
C	0.273535	0.419285	3.043729
H	1.271732	-0.014238	3.110968
H	-0.282155	0.106394	3.934394
H	0.386507	1.503647	3.090578
C	0.571023	-2.403786	1.529711
H	1.174142	-2.761449	0.692228
H	-0.035571	-3.246812	1.873260
H	1.243998	-2.136899	2.343493
C	-1.543390	-2.487786	-0.768841
H	-1.614155	-2.155366	-1.806662
H	-2.485188	-2.977402	-0.501061
H	-0.746073	-3.229059	-0.711003
C	-3.249095	0.129090	-0.706310
H	-4.106006	-0.473548	-0.387913
H	-3.031371	-0.116060	-1.747718
H	-3.546233	1.177746	-0.669431
Cl	-0.132536	-0.114166	-2.654502
H	-0.450191	1.977736	-0.935629
H	2.182969	-0.813235	-1.137356

D3

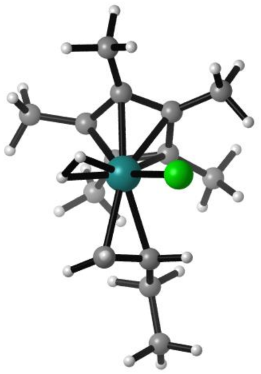

Ru	-0.046076	0.340188	-0.347821
C	2.215280	-0.002422	-0.490820
C	3.054562	-0.253947	0.724033
H	2.841535	-1.228740	1.167116
H	2.842180	0.503657	1.484835
C	4.528498	-0.194419	0.348258
C	1.933594	1.293008	-0.877228
H	2.207072	2.107991	-0.211126
H	-0.239373	2.062715	-0.731760
H	1.809262	1.543757	-1.923972
H	4.788163	0.792512	-0.041703
H	4.764024	-0.925746	-0.428691
H	5.168613	-0.400845	1.208366
C	-1.361708	0.669899	1.330333
C	-0.254486	-0.093402	1.793978
C	-0.259697	-1.342057	1.067673
C	-1.388506	-1.342300	0.199087
C	-2.064845	-0.102279	0.334327
C	-1.784955	1.986508	1.876096
H	-2.329638	2.575936	1.136957
H	-0.925826	2.574574	2.202437
H	-2.444028	1.853362	2.740333
C	0.583219	0.263763	2.969676
H	1.465271	-0.369906	3.052498
H	0.009424	0.151338	3.895803
H	0.925354	1.299982	2.921908
C	0.586173	-2.544327	1.300834
H	1.088699	-2.868868	0.386187
H	-0.032673	-3.378895	1.644490
H	1.347124	-2.370585	2.060605
C	-1.776633	-2.468728	-0.681884
H	-2.416354	-2.140466	-1.499981
H	-2.317116	-3.225350	-0.102899
H	-0.900724	-2.942284	-1.126748
C	-3.310269	0.286891	-0.380632
H	-4.191939	-0.161811	0.087250
H	-3.281229	-0.037220	-1.422439
H	-3.451493	1.368776	-0.377748
Cl	0.013760	-0.436217	-2.667964
H	-0.720796	1.669159	-1.310493
H	2.258656	-0.766145	-1.264695

D4

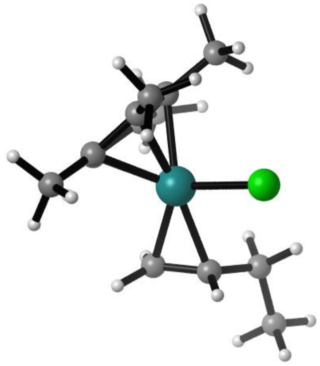

Ru	0.210102	-0.065746	-0.003414
C	2.291380	0.581235	-0.365448
C	3.205985	-0.566444	-0.685259
H	2.929667	-0.997707	-1.650496
H	3.073191	-1.357660	0.063432
C	4.657363	-0.113795	-0.705600
C	1.944191	0.901548	0.935749
H	2.322366	0.294071	1.755413
H	1.650885	1.912433	1.204250
H	4.955717	0.286479	0.267042
H	4.806952	0.675905	-1.446245
H	5.330223	-0.936826	-0.954875
C	-1.433377	0.723454	1.114103
C	-0.617656	-0.004979	2.030103
C	-0.510317	-1.340079	1.540967
C	-1.368016	-1.466531	0.382774
C	-1.938356	-0.196087	0.120935
C	-1.803104	2.155691	1.216648
H	-1.928264	2.600527	0.228412
H	-1.042442	2.729476	1.747890
H	-2.748492	2.277406	1.756347
C	-0.050047	0.517397	3.298332
H	0.837786	-0.034598	3.608518
H	-0.786777	0.426800	4.102483
H	0.222083	1.571130	3.224606
C	0.259651	-2.448423	2.156377
H	0.635068	-3.133389	1.394037
H	-0.364225	-3.029191	2.844337
H	1.117586	-2.076688	2.718903
C	-1.569440	-2.720732	-0.383418
H	-1.968973	-2.523041	-1.377037
H	-2.260684	-3.388148	0.140832
H	-0.625485	-3.252519	-0.517856
C	-2.873363	0.166742	-0.971611
H	-3.904042	0.204154	-0.605285
H	-2.824916	-0.547324	-1.792582
H	-2.627311	1.145749	-1.387072
Cl	0.265515	-0.413490	-2.343528
H	2.223675	1.350396	-1.133035

TS$_{R3\text{-}B1}$

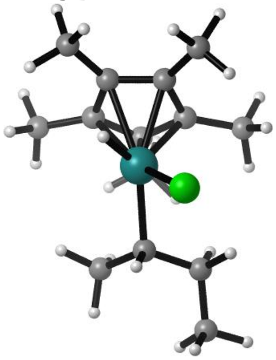

Ru	0.008070	-0.042518	-0.573449
C	1.974854	-0.756053	-0.205803
C	2.057337	-2.253072	0.046234
H	1.565055	-2.495971	0.999903
C	3.479928	-2.789585	0.086991
H	1.497478	-2.775860	-0.735564
C	2.690935	0.069671	0.845264
H	2.394834	-0.212163	1.860211
H	2.504220	1.141061	0.726750
H	3.779115	-0.063725	0.788481
C	-1.851552	1.209672	-0.057524
C	-0.907557	1.209663	1.036184
C	-0.750528	-0.124577	1.486798
C	-1.487884	-0.963657	0.576013
C	-2.208513	-0.117517	-0.350013
C	-2.378666	2.442031	-0.699598
H	-2.793316	2.241063	-1.687281
H	-1.602601	3.200892	-0.809749
H	-3.172218	2.874780	-0.082780
C	-0.326472	2.430282	1.647432
H	0.545360	2.193862	2.258018
H	-1.055966	2.941731	2.284488
H	-0.008359	3.139961	0.880769
C	-0.092151	-0.577315	2.738518
H	0.453373	-1.512902	2.603774
H	-0.845553	-0.749383	3.513866
H	0.609482	0.163792	3.120949
C	-1.623843	-2.439284	0.653627
H	-1.737928	-2.874902	-0.340077
H	-2.504219	-2.712762	1.245365
H	-0.752147	-2.899974	1.120784
C	-3.124123	-0.601110	-1.411808
H	-4.078179	-0.934630	-0.991745
H	-2.681143	-1.438398	-1.954341
H	-3.331490	0.179204	-2.144632
Cl	0.311457	-1.024015	-2.692176
H	-0.405452	1.089213	-1.575150
H	2.424113	-0.562548	-1.190340
H	4.011258	-2.534752	-0.834087
H	3.491932	-3.877037	0.189641
H	4.048421	-2.375298	0.923005

B1

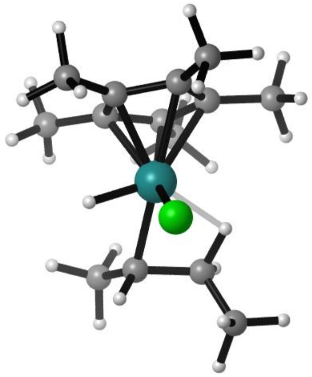

Ru	0.230361	-0.182283	-0.182731
Cl	0.865162	-2.455335	-0.653060
C	2.150668	0.522072	-0.666634
C	2.393575	0.141126	0.749486
H	1.478584	-0.419554	1.213946
H	2.462226	1.027931	1.388010
C	3.525159	-0.842261	0.984674
C	2.334415	1.962233	-1.042962
C	-1.275842	1.281517	-0.642808
C	-0.950744	1.421360	0.733347
C	-1.347733	0.200868	1.404034
C	-1.900001	-0.681504	0.462389
C	-1.803285	-0.055847	-0.827595
C	-1.202263	2.339099	-1.683578
H	-1.012112	1.913236	-2.669958
H	-0.403889	3.053025	-1.476787
H	-2.142014	2.897507	-1.737646
C	-0.474636	2.649255	1.423684
H	-0.080833	3.379835	0.716406
H	0.315378	2.430728	2.146602
H	-1.289710	3.131170	1.973846
C	-1.192702	-0.035485	2.864126
H	-1.166874	-1.098935	3.102369
H	-2.023855	0.413568	3.417344
H	-0.274617	0.414761	3.247204
C	-2.425029	-2.049662	0.692474
H	-1.856175	-2.781823	0.112255
H	-3.473540	-2.116819	0.388118
H	-2.361699	-2.336197	1.742111
C	-2.348898	-0.640435	-2.080064
H	-1.977364	-0.115808	-2.960762
H	-3.442529	-0.588144	-2.092494
H	-2.060134	-1.688677	-2.174611
H	1.924908	2.167942	-2.034744
H	3.396633	2.236158	-1.067920
H	1.842739	2.630440	-0.330129
H	3.354690	-1.749373	0.402805
H	3.612796	-1.119705	2.036530
H	4.470322	-0.396801	0.667092
H	2.658232	-0.156026	-1.354129
H	0.455332	-0.098159	-1.747234

TS_{B1-B2}

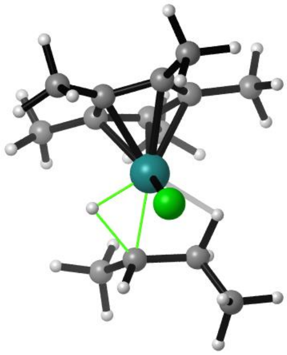

Ru	1.130760	1.746127	-1.030188
Cl	2.634538	0.265123	-2.198323
C	2.285284	1.488362	0.745797
C	1.366566	0.316271	0.865251
H	0.713722	0.155358	-0.084824
H	0.611664	0.497865	1.636737
C	2.056581	-1.025274	1.028975
C	2.208686	2.516322	1.838571
C	0.348402	3.741500	-1.164438
C	-0.691930	2.907421	-0.670197
C	-1.006187	1.947072	-1.703672
C	-0.169246	2.171578	-2.811149
C	0.722398	3.251556	-2.472572
C	0.896729	4.954653	-0.504521
H	1.942977	5.113523	-0.770943
H	0.841367	4.883760	0.582601
H	0.338760	5.846038	-0.807470
C	-1.452053	3.076401	0.596090
H	-0.897948	3.675220	1.320271
H	-1.675278	2.115402	1.065769
H	-2.409157	3.578938	0.420012
C	-2.051872	0.897630	-1.576294
H	-1.875246	0.065645	-2.258128
H	-3.041746	1.309788	-1.796760
H	-2.091590	0.494491	-0.562118
C	-0.135782	1.428237	-4.094638
H	0.861959	1.020911	-4.277763
H	-0.392165	2.088850	-4.928258
H	-0.839078	0.595490	-4.098407
C	1.715613	3.846219	-3.403465
H	2.450915	4.448862	-2.868914
H	1.227913	4.489130	-4.143895
H	2.259007	3.066187	-3.939207
H	2.784332	3.411115	1.591047
H	2.610316	2.118396	2.778017
H	1.175446	2.821728	2.026525
H	2.718687	-1.207352	0.180957
H	1.339199	-1.845499	1.088388
H	2.653516	-1.025132	1.943933
H	3.309053	1.156580	0.563462
H	2.501287	2.486448	-0.688931

B2

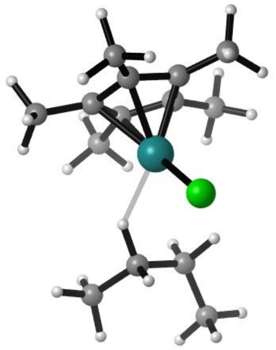

Ru	-0.354283	-0.362657	-0.443574
Cl	0.526354	-2.280595	-1.385143
C	2.500110	0.538307	-0.485870
C	2.711367	0.076218	0.946247
H	2.101421	-0.817025	1.127167
H	2.333344	0.847810	1.630669
C	4.160685	-0.231665	1.265690
C	3.214211	1.830599	-0.826707
C	-1.729284	1.222042	-0.747244
C	-1.196763	1.341639	0.571603
C	-1.479925	0.127817	1.271204
C	-2.227101	-0.741107	0.393881
C	-2.377528	-0.061304	-0.860282
C	-1.643262	2.244955	-1.819733
H	-1.628454	1.782296	-2.807700
H	-0.736394	2.845447	-1.724854
H	-2.498161	2.928464	-1.786114
C	-0.441101	2.504361	1.105226
H	0.104838	3.021680	0.313254
H	0.289298	2.194626	1.855680
H	-1.107233	3.234305	1.576150
C	-1.090764	-0.192088	2.667312
H	-0.860954	-1.253375	2.778511
H	-1.898150	0.047593	3.367252
H	-0.207911	0.370204	2.975468
C	-2.750718	-2.086619	0.734571
H	-2.800471	-2.724126	-0.148884
H	-3.754980	-2.023544	1.166310
H	-2.105740	-2.587760	1.458080
C	-3.079503	-0.583380	-2.058219
H	-2.636878	-0.193875	-2.976188
H	-4.137484	-0.301235	-2.049896
H	-3.019646	-1.671367	-2.104925
H	2.945579	2.185885	-1.823056
H	4.298668	1.703513	-0.807895
H	2.961638	2.617103	-0.108290
H	4.567361	-0.955765	0.554305
H	4.266629	-0.654821	2.265818
H	4.786103	0.662825	1.222231
H	2.782302	-0.259327	-1.179776
H	1.415176	0.734148	-0.659278

TS$_{C2\text{-}R1'}$

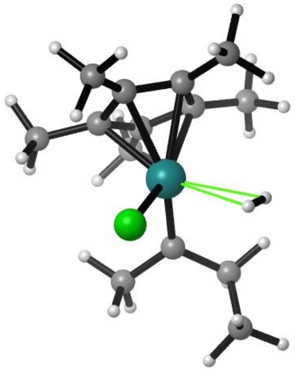

Ru	0.283626	-0.024692	0.091728
Cl	2.468377	-0.875283	0.602322
C	-0.101892	0.506105	1.861944
C	-1.116500	-0.175239	2.725991
H	-1.748260	-0.849126	2.141153
H	-1.780412	0.582993	3.167732
C	-0.426446	-0.956051	3.842741
C	0.629766	1.602217	2.543768
C	0.617260	1.558508	-1.289846
C	-0.751927	1.606023	-0.851254
C	-1.369444	0.383142	-1.237252
C	-0.411355	-0.355782	-2.048284
C	0.776150	0.371105	-2.108550
C	1.643705	2.621183	-1.155178
H	2.622186	2.186880	-0.941379
H	1.403304	3.317730	-0.350812
H	1.729600	3.201397	-2.080588
C	-1.405847	2.739540	-0.147375
H	-0.696447	3.303663	0.460059
H	-2.204294	2.394463	0.513077
H	-1.846929	3.437447	-0.865690
C	-2.789237	-0.004338	-1.031891
H	-2.889153	-1.081572	-0.880856
H	-3.409505	0.262152	-1.894999
H	-3.213090	0.489360	-0.155280
C	-0.698477	-1.674192	-2.668668
H	0.214424	-2.178325	-2.985476
H	-1.344061	-1.560926	-3.545672
H	-1.215741	-2.337566	-1.972356
C	2.049409	-0.007664	-2.770557
H	2.854847	-0.108110	-2.037208
H	2.346998	0.751368	-3.500081
H	1.962864	-0.959160	-3.295113
H	1.517307	1.139465	2.999603
H	0.060470	2.095403	3.342250
H	1.019689	2.345673	1.847215
H	0.309610	-1.647819	3.425460
H	-1.152680	-1.531392	4.418260
H	0.096322	-0.290649	4.531592
H	-0.662743	-2.192209	0.733357
H	0.080791	-2.340972	0.742056

R1′

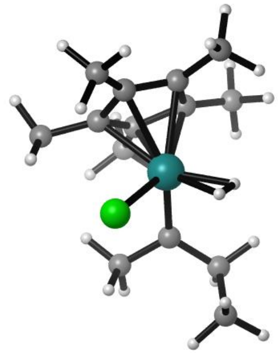

Ru	0.217452	-0.177513	0.134744
Cl	2.556525	-0.500615	0.704041
C	-0.187346	0.406085	1.887692
C	-1.111327	-0.263637	2.855356
H	-1.790418	-0.950314	2.345089
H	-1.731158	0.489154	3.362407
C	-0.295290	-1.025655	3.899763
C	0.517292	1.578189	2.462797
C	0.616295	1.510606	-1.262608
C	-0.739690	1.581274	-0.856136
C	-1.380055	0.368232	-1.255963
C	-0.433466	-0.385220	-2.059466
C	0.778625	0.304164	-2.059534
C	1.676850	2.536446	-1.093805
H	2.586436	2.088464	-0.684497
H	1.365230	3.338830	-0.423656
H	1.933227	2.992408	-2.055452
C	-1.410631	2.712913	-0.165927
H	-0.702588	3.349240	0.365516
H	-2.150698	2.362340	0.557122
H	-1.933954	3.342723	-0.891829
C	-2.828073	0.058603	-1.111864
H	-3.007346	-1.018001	-1.119149
H	-3.415115	0.494438	-1.928019
H	-3.227246	0.449651	-0.173535
C	-0.753330	-1.663939	-2.746493
H	0.148873	-2.198924	-3.042875
H	-1.348233	-1.485005	-3.647848
H	-1.334474	-2.329177	-2.104253
C	2.052271	-0.081991	-2.715611
H	2.861110	-0.152466	-1.982751
H	2.340634	0.662590	-3.463949
H	1.971249	-1.046116	-3.217819
H	1.450515	1.182908	2.891711
H	-0.035890	2.088917	3.260258
H	0.841361	2.294297	1.707867
H	0.398236	-1.717109	3.414787
H	-0.949553	-1.597687	4.558772
H	0.294270	-0.346260	4.517353
H	-0.604978	-1.649897	0.611400
H	0.216680	-1.858182	0.702287

$TS_{R1'-R2'}$

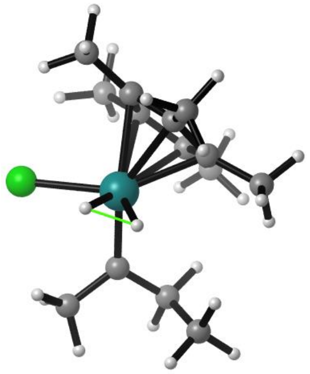

Ru	0.137242	-0.320924	-0.433970
C	1.941514	-0.779976	0.038044
C	2.614364	-2.044299	-0.348148
H	2.434688	-2.748967	0.475431
H	3.700284	-1.935386	-0.448343
H	2.188049	-2.511545	-1.233096
C	2.860958	0.138950	0.775761
H	3.529281	-0.448881	1.419805
H	2.313821	0.833584	1.414249
C	3.709124	0.935431	-0.214499
C	-1.314450	1.352729	-0.897045
C	-0.332144	1.809998	0.064915
C	-0.556585	1.103481	1.279218
C	-1.573053	0.150455	1.037146
C	-2.074382	0.332631	-0.302710
C	-1.507379	1.924551	-2.256900
H	-1.940257	1.193203	-2.940230
H	-0.564093	2.260225	-2.690220
H	-2.178369	2.788522	-2.223053
C	0.549336	2.996389	-0.110180
H	1.460423	2.919279	0.486455
H	0.036574	3.915505	0.192297
H	0.851782	3.118913	-1.151468
C	0.086714	1.358539	2.595356
H	0.433121	0.436731	3.067735
H	-0.624045	1.827878	3.282492
H	0.939341	2.033070	2.509290
C	-2.148680	-0.791626	2.027943
H	-2.230347	-1.796750	1.608451
H	-3.150630	-0.465079	2.324680
H	-1.534755	-0.854648	2.927073
C	-3.206167	-0.431993	-0.884465
H	-4.145568	-0.190984	-0.376643
H	-3.038426	-1.507723	-0.783831
H	-3.336182	-0.211917	-1.944488
Cl	-0.457166	-2.655449	-0.236827
H	0.334997	-0.865482	-1.932830
H	0.936800	0.224327	-1.709796
H	3.074839	1.557451	-0.849686
H	4.289668	0.278466	-0.865107
H	4.407733	1.585229	0.314905

R2′

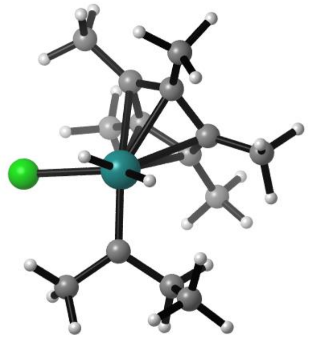

Ru	0.154743	-0.261941	-0.449386
C	1.947480	-0.756687	0.052446
C	2.566674	-2.095268	-0.108022
H	2.313591	-2.674230	0.789732
H	3.659912	-2.045226	-0.165461
H	2.156625	-2.659843	-0.942382
C	2.912869	0.234632	0.621457
H	3.545516	-0.267151	1.366496
H	2.394491	1.052586	1.125437
C	3.807572	0.815350	-0.470193
C	-1.334647	1.374930	-0.876145
C	-0.375591	1.826178	0.113369
C	-0.596649	1.081964	1.306863
C	-1.588742	0.117328	1.028760
C	-2.072190	0.318614	-0.318195
C	-1.528402	1.986129	-2.218105
H	-1.964741	1.274908	-2.919798
H	-0.584399	2.327233	-2.644995
H	-2.195841	2.851632	-2.158495
C	0.474496	3.041059	-0.013698
H	1.403447	2.953595	0.552846
H	-0.053501	3.924696	0.359228
H	0.743506	3.232594	-1.053203
C	0.065315	1.298661	2.620321
H	0.346971	0.356167	3.093793
H	-0.604374	1.823699	3.308530
H	0.966143	1.906261	2.526155
C	-2.154524	-0.867525	1.982029
H	-2.154727	-1.870891	1.549288
H	-3.188344	-0.605492	2.228115
H	-1.586097	-0.901503	2.911924
C	-3.179734	-0.454260	-0.932655
H	-4.130583	-0.240814	-0.434118
H	-2.990953	-1.527977	-0.851169
H	-3.296804	-0.214785	-1.989816
Cl	-0.445143	-2.604633	-0.348013
H	0.304211	-0.720103	-1.968414
H	1.078203	0.584961	-1.421051
H	3.214214	1.385630	-1.188194
H	4.332734	0.031559	-1.019573
H	4.555276	1.481859	-0.038007

TS$_{R2'-R3'}$

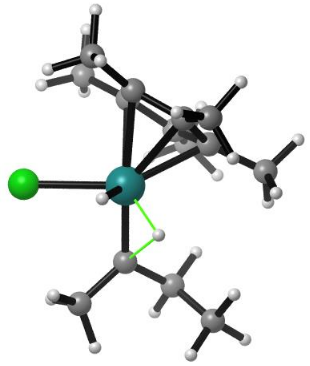

Ru	0.310343	0.162666	-0.294440
C	0.452496	2.062903	0.054738
C	1.648245	2.882803	-0.254772
H	2.245387	2.908928	0.666393
H	1.398237	3.919851	-0.506869
H	2.288378	2.440312	-1.013967
C	-0.670499	2.886618	0.603699
H	-0.243914	3.643692	1.274921
H	-1.357213	2.276733	1.194390
C	-1.451313	3.592181	-0.500670
C	-1.144368	-1.396611	-0.879302
C	-1.777283	-0.536563	0.093760
C	-1.117402	-0.726921	1.339459
C	-0.050893	-1.634468	1.126572
C	-0.077777	-2.074435	-0.238066
C	-1.619187	-1.610879	-2.273006
H	-0.808019	-1.941452	-2.922366
H	-2.027329	-0.695196	-2.703146
H	-2.405793	-2.371467	-2.304917
C	-3.042213	0.214817	-0.129502
H	-3.114872	1.099864	0.505424
H	-3.911641	-0.413742	0.088742
H	-3.131730	0.545562	-1.165495
C	-1.493173	-0.122595	2.644984
H	-0.617727	0.230209	3.193851
H	-2.004661	-0.852674	3.280123
H	-2.171813	0.722575	2.520950
C	0.894348	-2.138218	2.152356
H	1.925303	-2.069244	1.798372
H	0.682409	-3.187915	2.379049
H	0.823041	-1.571391	3.081047
C	0.844073	-3.087417	-0.813109
H	0.638390	-4.084798	-0.411322
H	1.882746	-2.839980	-0.579920
H	0.752902	-3.139848	-1.898516
Cl	2.668994	-0.040529	0.174587
H	0.990177	0.120420	-1.727301
H	-0.274092	1.200079	-1.376517
H	-1.942668	2.864096	-1.149884
H	-0.800399	4.209096	-1.123253
H	-2.219722	4.239195	-0.075143

R3′

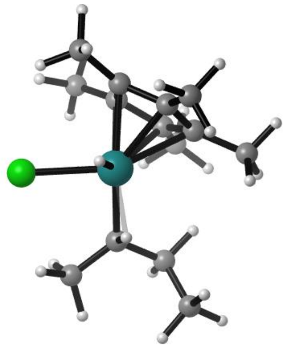

Ru	0.008383	-0.274274	-0.509997
C	1.942028	-0.687527	-0.055275
C	2.574657	-2.046286	-0.013792
H	2.354036	-2.500698	0.957924
H	3.666066	-1.992286	-0.103666
H	2.178870	-2.720709	-0.767933
C	2.827129	0.356229	0.587473
H	3.217437	-0.060865	1.524025
H	2.258619	1.249240	0.850574
C	3.983688	0.781729	-0.307464
C	-1.365132	1.410321	-0.881275
C	-0.421783	1.882974	0.092204
C	-0.545537	1.073378	1.246411
C	-1.533361	0.071215	0.983266
C	-2.084931	0.311968	-0.320082
C	-1.621566	2.077632	-2.184869
H	-2.142615	1.417369	-2.877942
H	-0.690616	2.387588	-2.662452
H	-2.235487	2.973684	-2.046240
C	0.429922	3.088191	-0.090474
H	1.142346	3.216990	0.724499
H	-0.185736	3.992328	-0.134774
H	0.996824	3.038553	-1.023217
C	0.151635	1.231030	2.549476
H	0.539633	0.278449	2.915531
H	-0.546033	1.608292	3.303741
H	0.984218	1.931992	2.495193
C	-2.008915	-0.945223	1.953947
H	-2.446601	-1.802376	1.444121
H	-2.765353	-0.513646	2.618216
H	-1.190746	-1.318853	2.571822
C	-3.217958	-0.436316	-0.922140
H	-4.160771	-0.193795	-0.422538
H	-3.055803	-1.513478	-0.843828
H	-3.328534	-0.200692	-1.981262
Cl	-0.413322	-2.642285	-0.624148
H	-0.467083	-0.525755	-2.001485
H	1.730957	-0.357504	-1.183463
H	3.604642	1.264574	-1.212908
H	4.595422	-0.067835	-0.617145
H	4.636773	1.493481	0.200945

$TS_{R3'-D1'}$

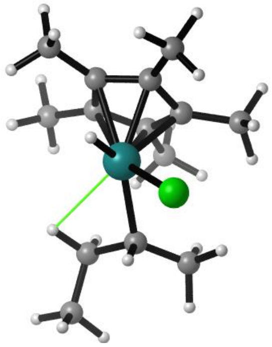

Ru	-0.153367	0.019276	0.015803
C	1.915857	-0.479894	0.055700
C	2.453519	-1.802106	0.515302
H	2.431496	-1.897877	1.605092
H	3.497953	-1.942307	0.210253
H	1.881427	-2.621876	0.074444
C	2.556061	0.747681	0.666807
H	2.565963	0.672196	1.760295
H	1.955482	1.641883	0.425615
C	3.973045	0.985460	0.160280
C	-1.859636	0.921041	1.076943
C	-0.773332	0.974885	2.004653
C	-0.336575	-0.345354	2.264810
C	-1.104272	-1.235776	1.446656
C	-2.092250	-0.449113	0.736692
C	-2.670940	2.097852	0.666749
H	-3.255610	1.888847	-0.228760
H	-2.040470	2.962235	0.452474
H	-3.364130	2.384985	1.464145
C	-0.267294	2.214882	2.644284
H	0.708755	2.065300	3.106273
H	-0.956402	2.555121	3.424504
H	-0.175719	3.027285	1.920285
C	0.632323	-0.745765	3.315902
H	1.013107	-1.753557	3.155725
H	0.138377	-0.736091	4.292800
H	1.486312	-0.070066	3.380073
C	-1.035380	-2.717817	1.441422
H	-1.288760	-3.114336	0.458143
H	-1.735798	-3.138279	2.170856
H	-0.035113	-3.074659	1.690774
C	-3.185253	-1.006899	-0.097725
H	-3.949670	-1.479898	0.527012
H	-2.799123	-1.753866	-0.793320
H	-3.665681	-0.229403	-0.692443
Cl	-0.116912	-1.472832	-1.871093
H	-1.032732	0.712592	-1.117650
H	2.010204	-0.424184	-1.039136
H	3.985237	1.070696	-0.928935
H	4.629543	0.156117	0.432757
H	4.400490	1.900429	0.575712

D1′

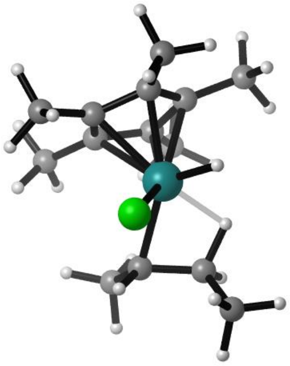

Ru	0.175435	-0.079105	0.289368
C	2.250961	-0.506138	0.573314
C	2.893785	-0.588411	1.924515
H	2.578849	0.227558	2.582170
H	3.984154	-0.515484	1.826780
H	2.688709	-1.534727	2.426093
C	2.290636	0.802572	-0.096886
H	2.689123	1.578297	0.567528
H	1.194990	1.279098	-0.250353
C	2.886735	0.857704	-1.487423
C	-1.476257	0.927894	1.250072
C	-0.552040	0.577369	2.290994
C	-0.491602	-0.846099	2.364864
C	-1.304943	-1.377773	1.333369
C	-1.944812	-0.289031	0.651148
C	-1.955888	2.306802	0.967162
H	-2.359784	2.390911	-0.041980
H	-1.148732	3.035595	1.059454
H	-2.743550	2.597454	1.670108
C	0.074656	1.551812	3.224244
H	0.867815	1.095989	3.817497
H	-0.665708	1.957152	3.921649
H	0.508462	2.398231	2.686521
C	0.199807	-1.661735	3.394277
H	0.667279	-2.547808	2.961239
H	-0.525392	-2.009101	4.136969
H	0.968432	-1.100999	3.924444
C	-1.540406	-2.815515	1.056256
H	-1.670789	-2.991465	-0.011600
H	-2.439009	-3.163355	1.576412
H	-0.700245	-3.428777	1.384114
C	-2.975031	-0.445570	-0.408838
H	-3.914107	-0.820538	0.010156
H	-2.640942	-1.148569	-1.174053
H	-3.182304	0.503298	-0.904650
Cl	0.404584	-1.655426	-1.549983
H	-0.394350	0.688363	-0.986659
H	2.514710	-1.337317	-0.078154
H	2.426956	0.097489	-2.120575
H	3.959985	0.662051	-1.436061
H	2.740564	1.834112	-1.953330

$TS_{D1'-D2'}$

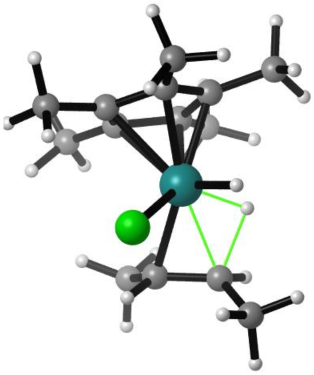

Ru	0.251709	-0.030062	0.281361
C	2.392192	-0.538182	0.542021
C	2.950621	-0.699215	1.919170
H	2.705259	0.152745	2.559828
H	4.043912	-0.760213	1.870572
H	2.607137	-1.610998	2.408013
C	2.367232	0.731278	-0.070329
H	2.688827	1.550442	0.579317
H	0.903782	1.423129	0.122848
C	2.697664	0.936932	-1.517914
C	-1.431322	0.924960	1.214690
C	-0.508437	0.580647	2.264897
C	-0.486346	-0.846646	2.378566
C	-1.298954	-1.378531	1.360103
C	-1.887289	-0.293124	0.622174
C	-1.919232	2.295039	0.902059
H	-2.229731	2.379770	-0.139660
H	-1.147345	3.045959	1.076138
H	-2.778433	2.551199	1.529942
C	0.106684	1.546119	3.215260
H	1.033769	1.161393	3.643038
H	-0.569830	1.766010	4.047719
H	0.339660	2.492844	2.724829
C	0.179141	-1.633262	3.448045
H	0.615503	-2.557235	3.064591
H	-0.549880	-1.912695	4.215329
H	0.969200	-1.069279	3.942899
C	-1.571409	-2.811567	1.094538
H	-1.280157	-3.074597	0.073495
H	-2.639612	-3.021090	1.203671
H	-1.027709	-3.461540	1.779997
C	-2.895235	-0.460791	-0.456506
H	-3.857978	-0.785436	-0.048479
H	-2.563778	-1.208975	-1.179393
H	-3.057086	0.471407	-0.998544
Cl	0.388812	-1.702132	-1.478631
H	-0.117283	0.768352	-1.038887
H	2.538865	-1.385830	-0.122009
H	2.342676	0.100446	-2.120072
H	3.782943	1.016144	-1.635309
H	2.252159	1.853386	-1.909199

D2′

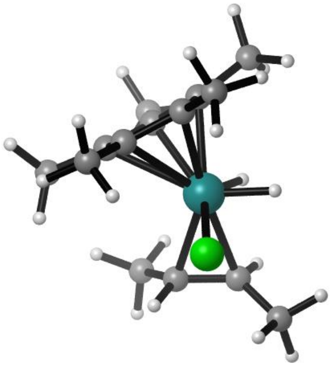

Ru	0.334720	0.418020	0.003280
C	2.034753	-0.636777	1.035034
C	1.804627	-1.003452	2.467285
H	1.473784	-0.137475	3.046633
H	2.744480	-1.350321	2.911346
H	1.081365	-1.807733	2.599370
C	2.509892	0.626780	0.706207
H	2.513357	1.341885	1.526425
H	0.534992	1.590190	1.034549
C	3.520094	0.942990	-0.346186
C	-1.662694	1.131523	0.310608
C	-1.367265	0.202718	1.382803
C	-1.318569	-1.113183	0.820693
C	-1.481898	-0.997151	-0.565536
C	-1.670063	0.393376	-0.903081
C	-2.036829	2.561465	0.471790
H	-1.748793	3.147706	-0.401096
H	-1.554137	3.009850	1.340561
H	-3.118995	2.661280	0.601616
C	-1.434057	0.516990	2.834816
H	-0.771630	-0.121391	3.420427
H	-2.451060	0.369432	3.212951
H	-1.154177	1.551907	3.033611
C	-1.204628	-2.397489	1.561884
H	-0.381626	-3.013233	1.190748
H	-2.121779	-2.983053	1.449185
H	-1.053035	-2.238671	2.629138
C	-1.531641	-2.110040	-1.538934
H	-0.726193	-2.011913	-2.273051
H	-2.480899	-2.090440	-2.082225
H	-1.435645	-3.079949	-1.050973
C	-1.979963	0.891749	-2.266789
H	-2.998237	0.617881	-2.561947
H	-1.288505	0.466734	-2.997212
H	-1.894594	1.977282	-2.322036
Cl	1.264849	-0.466766	-2.059375
H	0.774382	1.783733	-0.673203
H	2.285773	-1.454185	0.360803
H	3.575159	0.173808	-1.112072
H	4.499955	1.030671	0.136553
H	3.311200	1.894911	-0.837630

TS$_{D2'-D3'}$

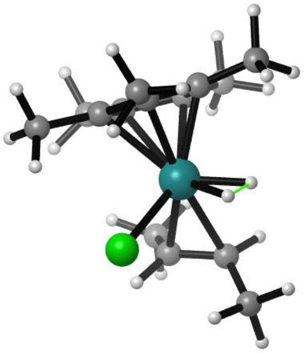

Ru	0.322161	0.344686	-0.076061
C	2.093048	-0.726960	0.917557
C	1.843415	-1.478435	2.182643
H	1.318218	-0.868227	2.922378
H	2.805642	-1.761048	2.624222
H	1.288145	-2.402292	2.028309
C	2.371133	0.628351	0.931130
H	2.203457	1.134019	1.882992
H	0.727699	1.847926	0.321576
C	3.370870	1.283266	0.031791
C	-1.639586	1.146962	0.267886
C	-1.330843	0.291973	1.381970
C	-1.300691	-1.054433	0.886653
C	-1.492644	-1.019085	-0.508171
C	-1.694005	0.343825	-0.912407
C	-1.960216	2.597636	0.347911
H	-1.679404	3.121891	-0.566348
H	-1.443523	3.079802	1.178797
H	-3.034623	2.741393	0.496613
C	-1.314841	0.700238	2.812076
H	-0.662309	0.059290	3.407296
H	-2.315250	0.648191	3.255177
H	-0.956529	1.724492	2.927051
C	-1.261261	-2.287034	1.713599
H	-0.681412	-3.082416	1.242981
H	-2.279103	-2.666402	1.850453
H	-0.847978	-2.107370	2.705155
C	-1.540573	-2.178816	-1.430459
H	-0.792777	-2.071256	-2.221128
H	-2.524611	-2.244690	-1.904232
H	-1.351595	-3.118673	-0.911991
C	-2.035610	0.774017	-2.292620
H	-3.064356	0.499138	-2.547899
H	-1.368923	0.303332	-3.017929
H	-1.939465	1.854075	-2.409473
Cl	1.369758	-0.520541	-2.103138
H	0.767764	1.707939	-0.833830
H	2.503693	-1.307884	0.095546
H	3.398041	0.800591	-0.945437
H	4.366698	1.208228	0.481840
H	3.158836	2.343963	-0.115537

D3′

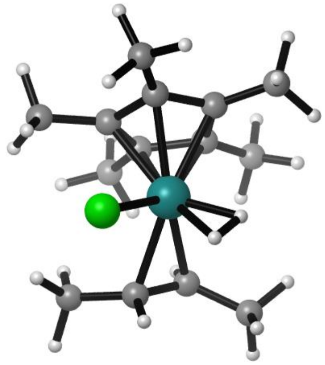

Ru	0.362891	-0.333911	-0.127498
C	2.202197	-0.325100	1.130711
C	2.057991	-1.141338	2.372884
H	1.249559	-0.772678	3.007653
H	2.980132	-1.105562	2.964336
H	1.864598	-2.188906	2.131161
C	1.754815	0.986824	0.989438
H	1.192111	1.402729	1.823330
H	1.058132	0.435391	-1.556077
C	2.474454	2.016630	0.172545
C	-1.293138	1.050898	0.260398
C	-1.240534	0.080500	1.322380
C	-1.442100	-1.202293	0.750267
C	-1.683446	-1.028078	-0.665268
C	-1.608216	0.353453	-0.949373
C	-1.265574	2.526766	0.432405
H	-0.939072	3.031809	-0.478579
H	-0.591761	2.830999	1.234912
H	-2.260505	2.911688	0.680995
C	-1.187272	0.402103	2.771118
H	-0.916953	-0.466135	3.371965
H	-2.171960	0.741420	3.108654
H	-0.480808	1.201364	3.001567
C	-1.506832	-2.504860	1.457354
H	-0.911329	-3.256278	0.933624
H	-2.539818	-2.863740	1.507402
H	-1.126437	-2.431383	2.476726
C	-2.026919	-2.124636	-1.603735
H	-2.013767	-1.787350	-2.640567
H	-3.028687	-2.510844	-1.389691
H	-1.317014	-2.949520	-1.513117
C	-1.823456	0.987695	-2.275893
H	-2.875738	1.257777	-2.406889
H	-1.550122	0.316014	-3.090250
H	-1.235256	1.900460	-2.383756
Cl	1.219668	-2.570348	-0.665087
H	1.602147	-0.182880	-1.409371
H	3.018901	-0.639115	0.482293
H	3.095352	1.553973	-0.597338
H	3.136561	2.609529	0.813039
H	1.790360	2.715086	-0.316980

D4′

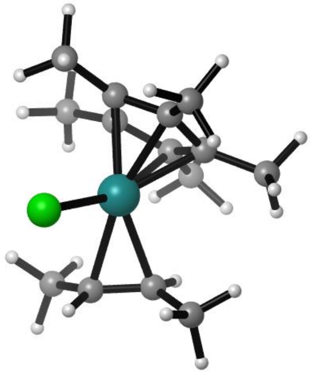

Ru	0.281824	-0.462667	-0.050415
C	2.274015	-0.525684	1.017383
C	2.175545	-1.511696	2.135455
H	1.411497	-1.221045	2.860784
H	3.130718	-1.586230	2.668394
H	1.937069	-2.512571	1.766573
C	1.796624	0.766214	1.077102
H	1.333159	1.106842	2.001398
C	2.319536	1.830787	0.162409
C	-1.271390	1.007905	0.109884
C	-1.298050	0.193441	1.281515
C	-1.499452	-1.157328	0.853484
C	-1.711297	-1.157628	-0.573603
C	-1.546932	0.166451	-1.035066
C	-1.157221	2.486987	0.064704
H	-0.656612	2.821894	-0.845876
H	-0.596317	2.877128	0.915505
H	-2.148122	2.952694	0.082249
C	-1.222664	0.651847	2.692266
H	-0.650515	-0.039751	3.314724
H	-2.223580	0.728967	3.128630
H	-0.756663	1.634576	2.777499
C	-1.594636	-2.348599	1.732619
H	-1.169813	-3.230222	1.249162
H	-2.639255	-2.573536	1.973930
H	-1.062077	-2.195733	2.672305
C	-1.985841	-2.364850	-1.391858
H	-1.696442	-2.219557	-2.432248
H	-3.050388	-2.617532	-1.363065
H	-1.425485	-3.225825	-1.023769
C	-1.617832	0.636993	-2.439424
H	-2.603740	1.055790	-2.666039
H	-1.423446	-0.174824	-3.140168
H	-0.875107	1.414859	-2.627595
Cl	1.421264	-1.720849	-1.677394
H	3.001289	-0.761334	0.244104
H	2.651648	1.399840	-0.785337
H	3.181413	2.332581	0.617422
H	1.577367	2.600313	-0.055292

TS$_{R3'-B1'}$

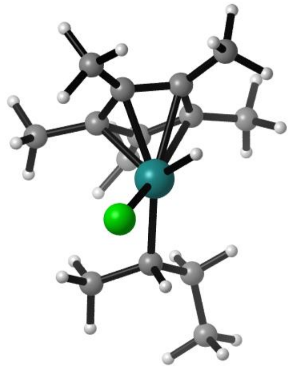

Ru	0.014614	-0.041514	-0.564151
C	1.987395	-0.737679	-0.187491
C	2.073730	-2.230106	0.056058
H	1.606773	-2.509874	1.008766
H	3.112281	-2.579437	0.107732
H	1.582172	-2.790873	-0.741403
C	2.697236	0.087730	0.875022
H	2.354132	-0.207917	1.873178
H	2.434172	1.147228	0.763063
C	4.213218	-0.040489	0.815670
C	-1.859189	1.185745	-0.043933
C	-0.926947	1.178999	1.059259
C	-0.760176	-0.160006	1.490422
C	-1.480381	-0.993164	0.560574
C	-2.201429	-0.140661	-0.359141
C	-2.391335	2.422805	-0.672680
H	-2.798422	2.232282	-1.665556
H	-1.620069	3.188563	-0.767976
H	-3.191679	2.841531	-0.054941
C	-0.365199	2.396196	1.694997
H	0.498773	2.159142	2.316415
H	-1.109019	2.893509	2.326669
H	-0.040789	3.118373	0.942761
C	-0.111801	-0.627708	2.741854
H	0.445771	-1.554564	2.597169
H	-0.873322	-0.823236	3.503723
H	0.576330	0.113717	3.147272
C	-1.602107	-2.470889	0.618429
H	-1.722273	-2.893824	-0.379935
H	-2.473502	-2.760805	1.215670
H	-0.721411	-2.929823	1.070430
C	-3.104812	-0.617480	-1.434483
H	-4.052789	-0.979740	-1.024606
H	-2.644248	-1.432916	-1.995451
H	-3.326593	0.175663	-2.149055
Cl	0.344671	-1.005677	-2.686825
H	-0.409862	1.088793	-1.562726
H	2.443531	-0.524773	-1.165206
H	4.587093	0.248654	-0.170079
H	4.537628	-1.066777	1.000939
H	4.700006	0.596869	1.557586

B1′

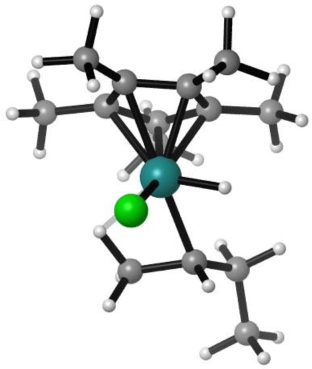

Ru	0.819117	-0.811332	-1.634936
C	2.157111	-0.332183	-0.093370
C	2.203519	-1.815265	-0.026732
H	1.752730	-2.226476	0.879091
H	3.193382	-2.233389	-0.219979
H	1.630324	-2.350042	-0.884415
C	1.669162	0.402594	1.124395
H	0.745470	-0.062628	1.490426
H	1.406511	1.428614	0.843998
C	2.706259	0.427619	2.235537
C	-0.750034	0.037873	-2.811774
C	-1.063333	0.204281	-1.406265
C	-1.208432	-1.094335	-0.848970
C	-1.058237	-2.052806	-1.924053
C	-0.816282	-1.363155	-3.122917
C	-0.580885	1.109301	-3.827425
H	0.228206	0.862614	-4.517186
H	-0.337551	2.065398	-3.363122
H	-1.496542	1.241778	-4.412994
C	-1.296350	1.497292	-0.712955
H	-1.056101	1.432685	0.349238
H	-2.343784	1.802691	-0.799608
H	-0.682463	2.292371	-1.138853
C	-1.629660	-1.434717	0.535721
H	-1.064088	-2.279057	0.937310
H	-2.688529	-1.712704	0.566637
H	-1.491995	-0.593261	1.215558
C	-1.168420	-3.525624	-1.748995
H	-0.663008	-4.068154	-2.548039
H	-2.218552	-3.834960	-1.743785
H	-0.736188	-3.850299	-0.800196
C	-0.582230	-1.933017	-4.472479
H	-0.638798	-3.021376	-4.467679
H	0.408874	-1.653118	-4.840822
H	-1.325626	-1.560562	-5.183178
Cl	2.657748	-1.107665	-3.155824
H	1.339520	0.679303	-1.739920
H	3.084460	0.088300	-0.483508
H	3.630520	0.899178	1.892451
H	2.958316	-0.585353	2.561483
H	2.353109	0.979736	3.108883

$TS_{B1'-B2'}$

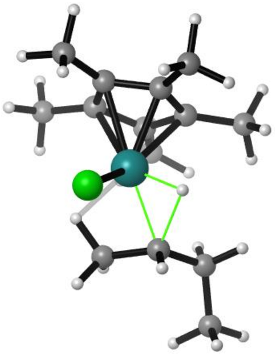

Ru	0.772848	-0.853743	-1.633334
C	2.167700	-0.298138	-0.122072
C	2.215288	-1.786060	-0.025077
H	1.780111	-2.169128	0.900233
H	3.210812	-2.194973	-0.207355
H	1.639979	-2.351452	-0.857088
C	1.697195	0.442023	1.102945
H	0.771373	-0.016571	1.471688
H	1.442385	1.472300	0.831633
C	2.745137	0.449468	2.202763
C	-0.757272	0.031730	-2.812992
C	-1.094784	0.186257	-1.415186
C	-1.261660	-1.113381	-0.863330
C	-1.094547	-2.066150	-1.937182
C	-0.816116	-1.372474	-3.128866
C	-0.556024	1.110852	-3.813867
H	0.244660	0.849278	-4.507739
H	-0.279997	2.051390	-3.335551
H	-1.466631	1.284568	-4.396938
C	-1.309854	1.477700	-0.712484
H	-1.078832	1.401876	0.351099
H	-2.351338	1.800886	-0.804319
H	-0.681228	2.266094	-1.129738
C	-1.688757	-1.454325	0.519281
H	-1.136844	-2.311605	0.912988
H	-2.752779	-1.711518	0.554141
H	-1.531351	-0.620051	1.204213
C	-1.204259	-3.539572	-1.768973
H	-0.683619	-4.077590	-2.561295
H	-2.253183	-3.852465	-1.782500
H	-0.785569	-3.865335	-0.814334
C	-0.567496	-1.939185	-4.477339
H	-0.583334	-3.028999	-4.467308
H	0.410174	-1.625310	-4.852450
H	-1.329337	-1.598062	-5.184571
Cl	2.627985	-1.072118	-3.157103
H	1.413041	0.592531	-1.424588
H	3.107610	0.102947	-0.504380
H	3.671060	0.913178	1.853442
H	2.989245	-0.566802	2.523513
H	2.405479	1.002069	3.080780

B2′

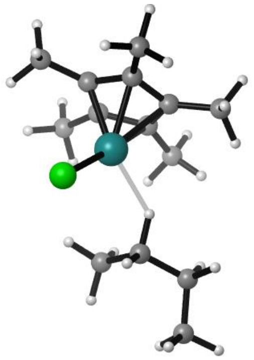

Ru	0.514925	-0.618331	-2.098944
C	2.240136	-0.389967	0.339737
C	2.295687	-1.898124	0.466485
H	1.411768	-2.278614	0.989744
H	3.175750	-2.215654	1.030348
H	2.346063	-2.383215	-0.511517
C	2.084961	0.334588	1.666933
H	1.163839	-0.009186	2.155738
H	1.947318	1.403403	1.474103
C	3.263918	0.137060	2.599292
C	-1.136575	0.059946	-3.181826
C	-1.375960	0.291812	-1.779201
C	-1.366572	-0.967331	-1.108717
C	-1.091815	-1.980477	-2.079813
C	-0.965117	-1.352003	-3.372800
C	-1.081898	1.097011	-4.240953
H	-0.412469	0.798586	-5.048588
H	-0.713615	2.045097	-3.846281
H	-2.073526	1.274985	-4.670008
C	-1.600678	1.615801	-1.145877
H	-1.243482	1.628791	-0.114054
H	-2.664750	1.873976	-1.130996
H	-1.076316	2.406730	-1.684260
C	-1.569388	-1.182434	0.347281
H	-1.025190	-2.060536	0.700422
H	-2.627004	-1.333576	0.586224
H	-1.221014	-0.326542	0.929600
C	-0.967806	-3.435172	-1.813261
H	-0.270800	-3.905064	-2.508981
H	-1.933721	-3.940458	-1.918152
H	-0.602372	-3.625316	-0.802609
C	-0.714395	-2.042383	-4.661725
H	-0.102722	-2.934329	-4.517345
H	-0.178439	-1.394753	-5.356486
H	-1.651888	-2.349579	-5.136650
Cl	2.537913	-0.493897	-3.208266
H	1.355707	-0.084602	-0.267771
H	3.117929	-0.020341	-0.199138
H	4.199188	0.418903	2.107598
H	3.360196	-0.902934	2.917839
H	3.165620	0.745317	3.499995

TS$_{I2\text{-}D4}$

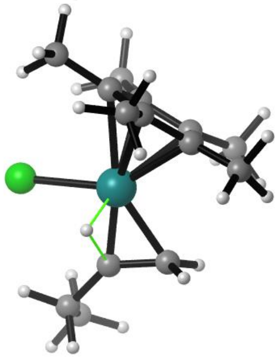

C	-1.203446	0.327775	1.411589
C	-1.102122	-1.068680	1.220224
C	-1.550281	-1.361053	-0.122287
C	-1.998628	-0.139721	-0.710060
C	-1.774277	0.896416	0.210787
Ru	0.274150	-0.115723	-0.220501
C	1.393892	-1.893160	-0.456515
H	1.115776	-2.487283	-1.326918
C	-0.660433	-2.060037	2.232981
C	-1.734845	-2.705210	-0.725397
C	-2.582505	-0.014195	-2.070244
C	-2.115100	2.328799	0.046294
C	-0.889087	1.101712	2.638156
Cl	1.199354	2.105084	-0.053077
C	2.024931	-0.648692	-0.608896
C	3.373384	-0.123047	-0.851059
H	0.774315	-0.047964	-1.780613
H	1.618691	-2.485910	0.431871
H	0.145891	-1.662543	2.852346
H	-0.299310	-2.978965	1.769787
H	-1.486248	-2.332171	2.897357
H	-1.132607	-3.462768	-0.223615
H	-1.455805	-2.709071	-1.781406
H	-2.782409	-3.017082	-0.662042
H	-2.428084	0.982797	-2.482892
H	-3.659867	-0.206360	-2.050767
H	-2.133963	-0.729764	-2.760824
H	-1.241352	2.960083	0.225899
H	-2.892543	2.614420	0.761484
H	-2.485065	2.544143	-0.956016
H	-1.806098	1.443876	3.128909
H	-0.289082	1.982283	2.396897
H	-0.330426	0.502093	3.357528
H	3.317281	0.925559	-1.145049
H	3.851210	-0.692613	-1.656616
C	4.188805	-0.255060	0.432380
H	4.212727	-1.289253	0.783974
H	3.749178	0.365484	1.214812
H	5.216418	0.070241	0.266348

I2

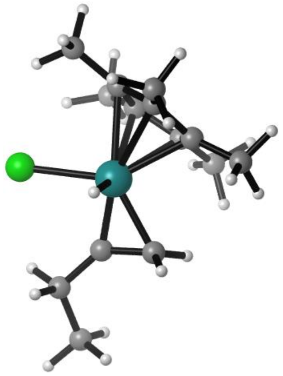

C	-1.479248	-1.514872	0.953550
C	-0.773376	-2.568405	0.329679
C	-0.776081	-2.313676	-1.091188
C	-1.569290	-1.152918	-1.332487
C	-1.973540	-0.640634	-0.088310
Ru	0.355028	-0.675944	-0.086302
C	2.132166	-1.673590	-0.599391
H	2.560498	-1.531401	-1.592290
C	-0.181593	-3.763363	0.981242
C	-0.262473	-3.225720	-2.145440
C	-1.894789	-0.604418	-2.674494
C	-2.839628	0.537013	0.152794
C	-1.767613	-1.327205	2.397549
Cl	0.214327	1.383022	1.146093
C	2.162883	-0.634473	0.343777
C	3.180624	0.184510	1.013673
H	0.716083	0.274079	-1.317124
H	2.179294	-2.709028	-0.255792
H	-0.128100	-3.641993	2.063175
H	0.829539	-3.970997	0.620821
H	-0.780099	-4.655571	0.772921
H	0.492440	-3.909684	-1.757534
H	0.186387	-2.669673	-2.970630
H	-1.076299	-3.830445	-2.559082
H	-2.074972	0.469986	-2.636438
H	-2.792587	-1.080648	-3.080990
H	-1.082102	-0.777413	-3.381219
H	-2.403600	1.192996	0.909141
H	-3.823974	0.214593	0.506535
H	-2.983367	1.124756	-0.753823
H	-2.830762	-1.481928	2.607408
H	-1.509333	-0.312505	2.711449
H	-1.199912	-2.022772	3.016065
H	2.760944	0.619201	1.924069
H	3.361213	1.048014	0.358247
C	4.472980	-0.568443	1.274686
H	4.909116	-0.937012	0.343860
H	4.300617	-1.430701	1.922489
H	5.206645	0.073624	1.764111

TS$_{C2\text{-}I2}$

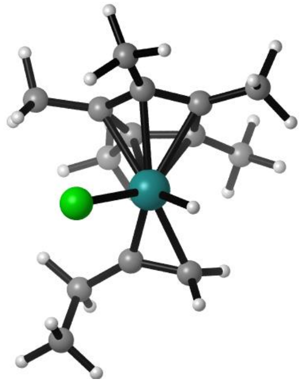

C	-1.248306	-1.195146	0.925618
C	-0.540423	-2.291365	0.407448
C	-0.769957	-2.334326	-1.014061
C	-1.725240	-1.321809	-1.343934
C	-1.979920	-0.583243	-0.167513
Ru	0.237291	-0.381906	-0.638652
C	2.068465	-0.725073	-1.694443
H	2.549507	0.110199	-2.201035
C	0.290192	-3.263690	1.165489
C	-0.261410	-3.381218	-1.938227
C	-2.333317	-1.115183	-2.684647
C	-2.906105	0.566832	-0.006202
C	-1.314619	-0.734071	2.334427
Cl	0.330220	1.764020	0.433655
C	2.020488	-0.736547	-0.298410
C	2.910895	-0.831366	0.858838
H	0.257830	0.556611	-1.924274
H	2.115853	-1.658153	-2.258687
H	0.679388	-2.832903	2.089210
H	1.141957	-3.612806	0.577446
H	-0.295901	-4.147177	1.436988
H	0.737636	-3.720146	-1.654717
H	-0.205240	-3.013895	-2.964293
H	-0.913754	-4.260431	-1.936487
H	-2.675864	-0.088477	-2.814366
H	-3.194216	-1.775722	-2.828425
H	-1.619337	-1.324611	-3.482453
H	-2.419826	1.387133	0.526916
H	-3.793842	0.272373	0.562516
H	-3.239275	0.951802	-0.970338
H	-2.319317	-0.873719	2.745496
H	-1.070476	0.329863	2.402936
H	-0.616863	-1.280234	2.970709
H	2.329677	-0.827936	1.786151
C	3.845691	0.380633	0.827000
H	3.499116	-1.756519	0.817867
H	3.262133	1.302906	0.839040
H	4.462927	0.372302	-0.072783
H	4.502460	0.371027	1.697026

I1

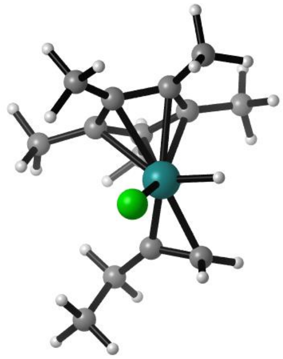

C	0.759316	0.227159	1.452982
C	0.557662	1.490730	0.827642
C	1.374331	1.547126	-0.353858
C	2.110353	0.316873	-0.425449
C	1.707705	-0.489980	0.669992
Ru	-0.117835	-0.028962	-0.671616
C	-1.889587	0.351423	-1.799737
H	-2.143071	-0.549175	-2.360297
C	-0.230174	2.636131	1.356079
C	1.562305	2.743993	-1.215381
C	3.132474	-0.053778	-1.441698
C	2.202674	-1.849545	0.996023
C	0.161190	-0.278682	2.717460
Cl	-0.324008	-2.353932	-1.183553
C	-1.929964	0.334939	-0.408981
C	-2.917042	0.573182	0.649167
H	0.236136	-0.004452	-2.214819
H	-2.044915	1.281250	-2.347986
H	-0.975533	2.321776	2.087099
H	-0.749968	3.170165	0.558204
H	0.429917	3.354116	1.852603
H	0.636469	3.313709	-1.311625
H	1.878045	2.463605	-2.220886
H	2.321950	3.415542	-0.801322
H	3.002165	-1.085572	-1.773684
H	4.145505	0.042964	-1.038886
H	3.063282	0.582222	-2.324725
H	2.772386	-2.280240	0.173508
H	1.375292	-2.529506	1.207637
H	2.848915	-1.813098	1.878935
H	0.911417	-0.346339	3.512026
H	-0.258618	-1.279026	2.581284
H	-0.636999	0.370828	3.079319
H	-2.442266	0.714275	1.623519
C	-3.830623	-0.652842	0.701077
H	-3.510346	1.469602	0.427766
H	-3.244869	-1.552883	0.899980
H	-4.344468	-0.795457	-0.250841
H	-4.579202	-0.539541	1.485943

TS$_{C2\text{-}I1}$

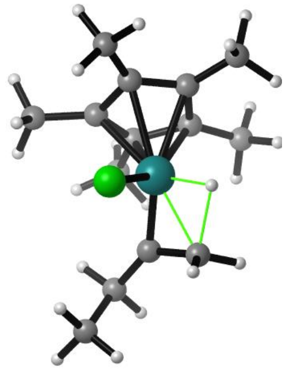

C	-0.911388	1.501128	0.580771
C	-0.454377	0.722305	1.670688
C	-1.069763	-0.576504	1.580668
C	-2.008553	-0.539413	0.492303
C	-1.886539	0.711083	-0.133164
Ru	0.195584	-0.324875	-0.196189
C	1.818797	-1.702824	-0.345001
H	2.163589	-1.853570	-1.370578
C	0.424840	1.174038	2.780160
C	-0.933324	-1.679751	2.568249
C	-2.931293	-1.643106	0.115487
C	-2.633874	1.196042	-1.319704
C	-0.565264	2.907603	0.250869
Cl	0.420823	0.246053	-2.505505
C	1.990816	-0.433460	0.240318
C	3.107520	0.321829	0.818797
H	0.134457	-1.799601	-0.819190
H	1.861327	-2.605021	0.267139
H	1.043095	2.024706	2.490018
H	1.090125	0.379693	3.124226
H	-0.177684	1.488661	3.637798
H	0.042661	-1.657897	3.056776
H	-1.036529	-2.654914	2.088089
H	-1.696415	-1.616024	3.351283
H	-3.159839	-1.629199	-0.950618
H	-3.877515	-1.568007	0.660894
H	-2.499092	-2.618536	0.343743
H	-3.252248	0.411674	-1.755717
H	-1.950318	1.546020	-2.096670
H	-3.288031	2.028148	-1.041667
H	-1.358503	3.599868	0.552979
H	-0.414841	3.025837	-0.824380
H	0.355344	3.220226	0.746788
H	2.759659	1.266994	1.245462
C	4.121777	0.595015	-0.291726
H	3.586768	-0.252505	1.622831
H	3.643689	1.118238	-1.122308
H	4.535926	-0.337431	-0.678863
H	4.943133	1.207995	0.080856

$TS_{C2\text{-}E2}$

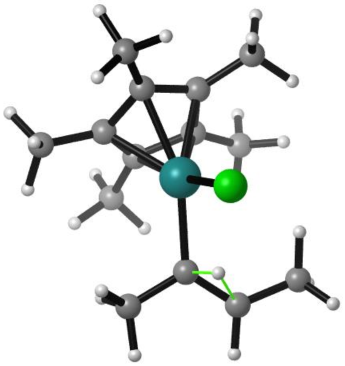

Ru	0.084553	-0.157825	-0.162085
Cl	1.110585	-2.200838	-0.817647
C	1.943961	0.794189	-0.499449
C	3.100345	0.615500	0.241043
H	4.012923	1.135835	-0.073753
H	2.455720	-0.318570	-0.649957
C	3.200208	-0.177119	1.491339
C	2.043420	1.683369	-1.703868
C	-1.726327	0.825587	-0.659735
C	-1.122680	1.523000	0.432876
C	-0.976728	0.586465	1.500696
C	-1.579044	-0.668672	1.091902
C	-2.052095	-0.516218	-0.231362
C	-2.048256	1.394716	-1.992099
H	-1.955848	0.639387	-2.774657
H	-1.379478	2.218608	-2.247654
H	-3.074435	1.777248	-2.022133
C	-0.733141	2.956356	0.466978
H	-0.543839	3.345371	-0.535544
H	0.174853	3.109687	1.055125
H	-1.520749	3.574212	0.910624
C	-0.407470	0.870619	2.841835
H	0.066277	-0.016195	3.267508
H	-1.183071	1.197844	3.543255
H	0.347274	1.658272	2.791900
C	-1.634632	-1.894388	1.927372
H	-1.737800	-2.789026	1.312625
H	-2.474692	-1.864173	2.628959
H	-0.717883	-2.010565	2.510411
C	-2.698460	-1.551614	-1.077034
H	-2.346783	-1.490151	-2.109217
H	-3.787775	-1.441270	-1.088242
H	-2.464590	-2.555739	-0.721537
H	1.594847	1.213120	-2.581021
H	3.069186	1.987623	-1.946750
H	1.463216	2.588597	-1.506631
H	2.329247	-0.824468	1.617947
H	3.229731	0.515655	2.338960
H	4.113710	-0.773186	1.529564

XYZ coordinates (Å) for the molecules present in the hydrogenation of the actual substrate using the neutral catalyst

Alkyne 8b

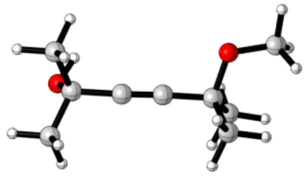

O	2.196052000	-1.006168000	-0.134210000
O	-2.905270000	-0.172328000	1.369312000
H	-2.399116000	-0.853764000	1.822197000
C	-0.980049000	0.063820000	0.006137000
C	0.216604000	0.149225000	-0.001352000
C	1.672858000	0.312776000	-0.003894000
C	3.583181000	-1.147331000	-0.041105000
H	3.807926000	-2.190328000	-0.265312000
H	3.964502000	-0.926788000	0.963898000
H	4.126257000	-0.522904000	-0.761963000
C	-2.439042000	-0.096411000	0.028097000
C	-3.120459000	1.118013000	-0.571746000
H	-4.202786000	0.987575000	-0.510812000
H	-2.833705000	1.242184000	-1.616800000
H	-2.844413000	2.016091000	-0.018699000
C	-2.822207000	-1.361351000	-0.727026000
H	-2.350194000	-2.235194000	-0.272212000
H	-2.501089000	-1.307537000	-1.769044000
H	-3.906319000	-1.486722000	-0.690661000
C	2.071202000	1.180483000	-1.193268000
H	3.145821000	1.373223000	-1.198788000
H	1.558063000	2.141911000	-1.140495000
H	1.792365000	0.686775000	-2.125041000
C	2.102379000	0.965044000	1.305536000
H	3.170604000	1.191231000	1.302360000
H	1.879505000	0.306044000	2.146383000
H	1.559526000	1.900574000	1.445902000

A1

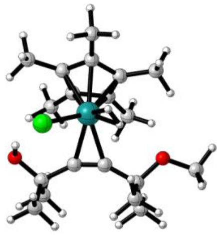

Ru	-0.576387000	-0.252017000	-0.440713000
Cl	-1.050383000	1.407521000	-2.189185000
O	2.387666000	-2.022211000	-0.132668000
O	-0.394366000	3.235091000	0.291596000
H	-0.799653000	2.911248000	-0.533869000
C	0.963780000	1.241187000	0.131043000
C	1.543529000	0.157011000	-0.060228000
C	2.764175000	-0.653091000	-0.283252000
C	3.414685000	-2.971081000	-0.135585000
H	2.937087000	-3.952226000	-0.142470000
H	4.048770000	-2.908349000	0.757454000
H	4.057447000	-2.903620000	-1.021788000
C	0.914553000	2.720884000	0.327625000
C	1.481527000	3.068604000	1.693083000
H	1.450388000	4.152109000	1.827855000
H	2.515855000	2.729704000	1.779446000
H	0.895585000	2.602998000	2.485777000
C	1.756095000	3.365926000	-0.770522000
H	1.346706000	3.116019000	-1.751024000
H	2.792023000	3.018392000	-0.726032000
H	1.739493000	4.450820000	-0.640596000
C	3.266692000	-0.382971000	-1.697370000
H	4.172656000	-0.952645000	-1.915561000
H	3.498837000	0.677510000	-1.809145000
H	2.502766000	-0.640471000	-2.433212000
C	3.808497000	-0.243486000	0.747110000
H	4.768085000	-0.732000000	0.562832000
H	3.467932000	-0.492814000	1.754568000
H	3.967903000	0.834915000	0.694941000
C	-2.607236000	-0.091583000	0.469750000
C	-1.637248000	0.041759000	1.501221000
C	-0.847375000	-1.157844000	1.528281000
C	-1.334731000	-2.024495000	0.507599000
C	-2.427318000	-1.355873000	-0.163030000
C	-3.639825000	0.913513000	0.121566000
H	-3.794958000	0.966582000	-0.956811000
H	-3.354023000	1.908967000	0.463245000
H	-4.594727000	0.655763000	0.591091000
C	-1.582316000	1.154387000	2.485030000
H	-1.571535000	2.128966000	1.994539000
H	-0.692704000	1.086497000	3.112100000
H	-2.452545000	1.109650000	3.147483000
C	0.230936000	-1.457252000	2.504121000
H	0.942329000	-2.176992000	2.097268000
H	-0.181713000	-1.865106000	3.432155000
H	0.791182000	-0.554491000	2.758726000
C	-0.851694000	-3.404081000	0.234531000
H	-1.029496000	-3.691192000	-0.803228000
H	-1.366778000	-4.130709000	0.870403000
H	0.220131000	-3.486855000	0.421060000
C	-3.296350000	-1.909274000	-1.236226000
H	-3.545642000	-1.142192000	-1.971790000
H	-4.234684000	-2.297476000	-0.827002000
H	-2.805844000	-2.726275000	-1.767419000
H	-0.341618000	-1.105122000	-1.970701000
H	0.399594000	-1.207189000	-1.572655000

TS$_{A2\text{-}A3}$

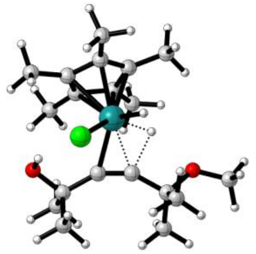

Ru	-0.488259000	-0.359553000	-0.355651000
Cl	-0.607069000	1.082953000	-2.335256000
O	2.772525000	-1.914514000	0.395121000
O	-0.407536000	3.099646000	0.128528000
H	-0.682863000	2.743284000	-0.735747000
C	0.976725000	1.131877000	0.213066000
C	1.636244000	0.081425000	0.036310000
C	2.922230000	-0.624817000	-0.196352000
C	3.762682000	-2.861863000	0.115661000
H	3.560376000	-3.727134000	0.747330000
H	4.772114000	-2.501383000	0.350594000
H	3.745859000	-3.189685000	-0.930330000
C	0.894206000	2.616244000	0.365485000
C	1.262208000	3.015574000	1.782942000
H	1.185753000	4.100992000	1.878868000
H	2.284547000	2.709712000	2.013101000
H	0.589066000	2.554941000	2.506920000
C	1.860926000	3.249800000	-0.631256000
H	1.587925000	2.965200000	-1.649151000
H	2.889651000	2.932647000	-0.443252000
H	1.804081000	4.336732000	-0.536161000
C	3.162067000	-0.699950000	-1.697373000
H	4.144960000	-1.118383000	-1.924806000
H	3.109535000	0.305200000	-2.120405000
H	2.390898000	-1.304922000	-2.177992000
C	4.031538000	0.145169000	0.503979000
H	5.000404000	-0.332414000	0.343938000
H	3.833166000	0.194942000	1.575857000
H	4.094986000	1.160708000	0.109269000
C	-2.660924000	0.167863000	0.211638000
C	-1.929232000	0.182686000	1.416232000
C	-1.275779000	-1.082358000	1.553331000
C	-1.704025000	-1.921629000	0.460944000
C	-2.530183000	-1.129985000	-0.396732000
C	-3.478736000	1.275401000	-0.339732000
H	-3.202522000	1.481771000	-1.377401000
H	-3.346111000	2.193865000	0.232245000
H	-4.540379000	1.010612000	-0.323758000
C	-1.890589000	1.295450000	2.398625000
H	-1.738995000	2.258426000	1.908608000
H	-1.091240000	1.154086000	3.127160000
H	-2.832592000	1.336983000	2.954487000
C	-0.427174000	-1.494872000	2.702721000
H	0.186327000	-2.360373000	2.449578000
H	-1.033195000	-1.751349000	3.577131000
H	0.255437000	-0.692812000	2.993651000
C	-1.416138000	-3.373962000	0.315777000
H	-1.450106000	-3.682332000	-0.729475000
H	-2.150309000	-3.969945000	0.866455000
H	-0.425788000	-3.625813000	0.698377000
C	-3.256484000	-1.562502000	-1.618482000
H	-3.171647000	-0.809262000	-2.404242000
H	-4.320688000	-1.715634000	-1.411524000
H	-2.852907000	-2.495649000	-2.012327000
H	-0.076090000	-1.346515000	-1.537629000
H	0.726628000	-1.344742000	-0.041271000

A3

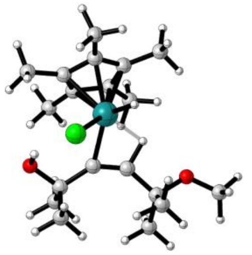

Ru	-0.628357000	-0.327766000	-0.384171000
Cl	-0.585830000	1.164013000	-2.322059000
O	2.955407000	-2.021336000	0.236171000
O	-0.189732000	3.057753000	0.249033000
H	-0.539161000	2.768863000	-0.612965000
C	0.926133000	0.940663000	0.233526000
C	1.610280000	-0.148785000	0.095333000
C	3.007930000	-0.651139000	-0.168747000
C	4.040028000	-2.834259000	-0.104418000
H	3.892897000	-3.787769000	0.403459000
H	5.001705000	-2.421809000	0.226128000
H	4.098178000	-3.027209000	-1.182275000
C	1.064866000	2.422308000	0.396745000
C	1.552090000	2.755785000	1.795905000
H	1.579742000	3.841348000	1.913046000
H	2.552956000	2.354857000	1.963812000
H	0.882793000	2.342866000	2.551333000
C	2.021691000	2.990418000	-0.648102000
H	1.659534000	2.761494000	-1.651734000
H	3.032594000	2.593014000	-0.541106000
H	2.061120000	4.075119000	-0.523330000
C	3.287987000	-0.517044000	-1.656986000
H	4.293955000	-0.859973000	-1.910368000
H	3.196735000	0.527726000	-1.957028000
H	2.557208000	-1.093012000	-2.230529000
C	4.021591000	0.094305000	0.678094000
H	5.030457000	-0.281404000	0.495087000
H	3.786160000	-0.029576000	1.736656000
H	4.023100000	1.158315000	0.441383000
C	-2.654827000	0.319599000	0.279564000
C	-1.901054000	0.183831000	1.473890000
C	-1.387486000	-1.143057000	1.525175000
C	-1.904520000	-1.869366000	0.394518000
C	-2.684485000	-0.954815000	-0.388567000
C	-3.391787000	1.525731000	-0.170948000
H	-3.199920000	1.726652000	-1.227289000
H	-3.100709000	2.409115000	0.397509000
H	-4.469729000	1.380545000	-0.048275000
C	-1.722674000	1.236357000	2.505291000
H	-1.501157000	2.203889000	2.052034000
H	-0.914890000	0.987963000	3.194847000
H	-2.638485000	1.335251000	3.096533000
C	-0.522249000	-1.696064000	2.599312000
H	0.017740000	-2.580150000	2.256584000
H	-1.107652000	-1.979670000	3.479090000
H	0.224582000	-0.964861000	2.918412000
C	-1.742691000	-3.329491000	0.161874000
H	-1.911704000	-3.584217000	-0.884455000
H	-2.451941000	-3.901814000	0.767825000
H	-0.736895000	-3.665375000	0.420057000
C	-3.488019000	-1.243825000	-1.604273000
H	-3.345847000	-0.463816000	-2.354545000
H	-4.555506000	-1.293606000	-1.366476000
H	-3.198240000	-2.191878000	-2.057956000
H	-0.496745000	-1.341697000	-1.616794000
H	0.961716000	-1.143941000	-0.034912000

TS$_{A3\text{-}E1}$

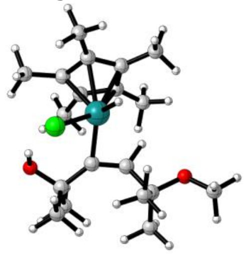

Ru	-0.643207000	-0.244586000	-0.374600000
Cl	-0.685697000	1.339390000	-2.237770000
O	3.030169000	-2.051926000	0.144957000
O	-0.107247000	3.046122000	0.317057000
H	-0.501589000	2.799089000	-0.539276000
C	0.887138000	0.871426000	0.303223000
C	1.607884000	-0.256978000	0.340917000
C	2.995764000	-0.648526000	-0.137951000
C	4.094669000	-2.789219000	-0.377822000
H	4.031231000	-3.787536000	0.056408000
H	5.074885000	-2.370696000	-0.115410000
H	4.042777000	-2.888704000	-1.468712000
C	1.118526000	2.351889000	0.407704000
C	1.650900000	2.656249000	1.801821000
H	1.742292000	3.738721000	1.911620000
H	2.625493000	2.194273000	1.963654000
H	0.960996000	2.291605000	2.565308000
C	2.070220000	2.893317000	-0.655394000
H	1.682322000	2.683562000	-1.653301000
H	3.077236000	2.483426000	-0.569918000
H	2.128212000	3.976068000	-0.522632000
C	3.129491000	-0.389950000	-1.627989000
H	4.126098000	-0.649349000	-1.992952000
H	2.951190000	0.661841000	-1.849191000
H	2.383312000	-0.971983000	-2.174555000
C	4.075537000	0.049868000	0.671365000
H	5.068878000	-0.286160000	0.366002000
H	3.944333000	-0.166384000	1.733430000
H	4.041948000	1.129962000	0.529960000
C	-2.650581000	0.296141000	0.341375000
C	-1.881569000	0.051842000	1.514087000
C	-1.396023000	-1.283980000	1.455689000
C	-1.912804000	-1.896647000	0.261244000
C	-2.699955000	-0.919465000	-0.427640000
C	-3.381897000	1.542835000	0.008303000
H	-3.250443000	1.802885000	-1.043708000
H	-3.030972000	2.384376000	0.605930000
H	-4.453328000	1.415735000	0.193391000
C	-1.658866000	1.022295000	2.616764000
H	-1.476133000	2.025909000	2.228575000
H	-0.804855000	0.737494000	3.233225000
H	-2.537280000	1.062353000	3.268636000
C	-0.579057000	-1.950083000	2.502313000
H	-0.092309000	-2.849571000	2.121584000
H	-1.198476000	-2.243090000	3.355131000
H	0.203636000	-1.287769000	2.882132000
C	-1.736653000	-3.325956000	-0.108137000
H	-1.936055000	-3.488075000	-1.167223000
H	-2.415559000	-3.964019000	0.466146000
H	-0.716647000	-3.662589000	0.086309000
C	-3.499222000	-1.098662000	-1.667162000
H	-3.342446000	-0.265056000	-2.354375000
H	-4.568845000	-1.152694000	-1.440444000
H	-3.219187000	-2.012020000	-2.192691000
H	-0.456564000	-1.189569000	-1.663498000
H	1.089438000	-1.171842000	0.672585000

E1

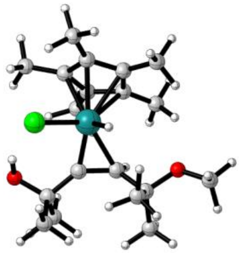

Ru	-0.509964000	0.005261000	-0.272140000
Cl	-1.203112000	1.668354000	-1.910136000
O	2.489985000	-2.157564000	0.056135000
O	0.058553000	3.366684000	0.346910000
H	-0.502711000	3.095243000	-0.401608000
C	0.900940000	1.130640000	0.187953000
C	1.473607000	-0.118902000	0.474552000
C	2.680774000	-0.760680000	-0.195805000
C	3.377986000	-3.049665000	-0.548953000
H	3.146728000	-4.042264000	-0.159202000
H	4.428626000	-2.834282000	-0.314979000
H	3.267751000	-3.078993000	-1.639930000
C	1.222045000	2.581247000	0.313364000
C	1.896319000	2.793454000	1.663432000
H	2.049768000	3.864379000	1.808808000
H	2.857044000	2.279424000	1.717090000
H	1.252758000	2.428722000	2.467620000
C	2.134084000	3.034248000	-0.823971000
H	1.645555000	2.876427000	-1.787608000
H	3.092402000	2.509484000	-0.817530000
H	2.320812000	4.102832000	-0.697007000
C	2.797308000	-0.478598000	-1.679908000
H	3.769362000	-0.797500000	-2.062478000
H	2.695919000	0.588817000	-1.880225000
H	2.012472000	-0.991661000	-2.236707000
C	3.931456000	-0.279251000	0.532759000
H	4.826003000	-0.795651000	0.177549000
H	3.837497000	-0.449925000	1.607619000
H	4.084541000	0.789070000	0.362187000
C	-2.590166000	0.025292000	0.605706000
C	-1.706931000	-0.387058000	1.628204000
C	-1.084566000	-1.605492000	1.199956000
C	-1.640506000	-1.977153000	-0.064706000
C	-2.554569000	-0.971415000	-0.447287000
C	-3.477860000	1.213635000	0.614209000
H	-3.511140000	1.683030000	-0.370633000
H	-3.133043000	1.965946000	1.324746000
H	-4.498880000	0.929209000	0.888432000
C	-1.453276000	0.308677000	2.917702000
H	-1.645936000	1.379120000	2.835145000
H	-0.418451000	0.186182000	3.244517000
H	-2.092218000	-0.089415000	3.712231000
C	-0.193787000	-2.472065000	2.013026000
H	0.622571000	-2.882304000	1.417170000
H	-0.770449000	-3.305115000	2.428264000
H	0.245759000	-1.933237000	2.853297000
C	-1.309207000	-3.232505000	-0.787427000
H	-1.564539000	-3.166285000	-1.845006000
H	-1.854521000	-4.080781000	-0.361376000
H	-0.242355000	-3.452470000	-0.715249000
C	-3.411880000	-0.947438000	-1.658577000
H	-3.345769000	0.016256000	-2.168169000
H	-4.459830000	-1.116181000	-1.392153000
H	-3.118313000	-1.719329000	-2.370446000
H	0.106354000	-0.641907000	-1.594483000
H	1.448623000	-0.425110000	1.526858000

TS$_{E1-E2}$

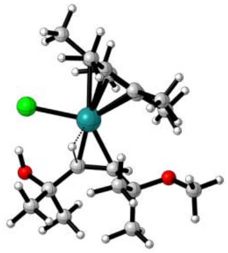

Ru	-0.555269000	0.214441000	-0.058528000
Cl	-1.612802000	2.243314000	-0.912994000
O	0.652760000	3.582855000	0.544779000
H	-0.178210000	3.406632000	0.070920000
C	1.026731000	1.210325000	0.186005000
C	1.479954000	-0.078151000	0.538872000
H	0.432862000	0.636859000	-1.329039000
H	1.601222000	-0.226614000	1.617380000
C	2.533200000	-0.920467000	-0.185937000
C	1.590511000	2.608463000	0.179228000
C	2.666668000	2.710024000	1.252924000
H	2.953171000	3.759834000	1.332710000
H	3.549097000	2.117648000	1.020121000
H	2.267198000	2.394726000	2.218789000
C	2.166504000	2.923993000	-1.199015000
H	1.372714000	2.905914000	-1.948641000
H	2.948385000	2.218627000	-1.493795000
H	2.595669000	3.927924000	-1.168460000
C	2.375255000	-0.987141000	-1.691317000
H	3.198294000	-1.546532000	-2.141707000
H	2.398520000	0.016622000	-2.118689000
H	1.431186000	-1.449976000	-1.980302000
C	3.916585000	-0.373976000	0.153094000
H	4.701454000	-1.038440000	-0.214683000
H	4.033959000	-0.267172000	1.233466000
H	4.074277000	0.598081000	-0.316275000
C	-2.802799000	-0.407658000	0.011797000
C	-2.194680000	-0.545315000	1.294716000
C	-1.155546000	-1.506514000	1.171261000
C	-1.113082000	-1.939207000	-0.207597000
C	-2.128443000	-1.236655000	-0.914759000
C	-4.011205000	0.401240000	-0.261809000
H	-4.111830000	0.646298000	-1.318022000
H	-4.012143000	1.335520000	0.298411000
H	-4.891935000	-0.176175000	0.040758000
C	-2.640365000	0.151968000	2.528368000
H	-2.832589000	1.209099000	2.334263000
H	-1.886544000	0.093639000	3.313834000
H	-3.564471000	-0.286781000	2.919681000
C	-0.370175000	-2.075332000	2.294355000
H	0.584546000	-2.475888000	1.953500000
H	-0.931705000	-2.879898000	2.779650000
H	-0.162469000	-1.319544000	3.054997000
C	-0.380370000	-3.116869000	-0.734814000
H	-0.086226000	-2.985653000	-1.777758000
H	-1.031552000	-3.996512000	-0.694548000
H	0.514567000	-3.324281000	-0.154228000
C	-2.447808000	-1.367024000	-2.359656000
H	-2.725113000	-0.401134000	-2.785891000
H	-3.278240000	-2.059595000	-2.529567000
H	-1.587464000	-1.735680000	-2.920575000
O	2.402697000	-2.212196000	0.423954000
C	3.151775000	-3.267583000	-0.100427000
H	4.227561000	-3.058037000	-0.137428000
H	3.001791000	-4.121640000	0.562532000
H	2.823582000	-3.559590000	-1.106860000

TS$_{C1-C2}$

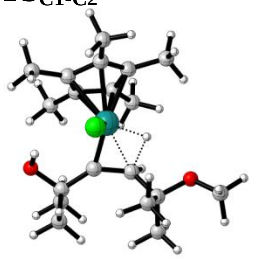

Ru	-0.431037000	-0.221794000	-0.174750000
Cl	-0.254290000	-0.427191000	-2.565243000
O	2.841813000	-1.953964000	0.602189000
O	-0.241953000	3.112508000	-0.501349000
H	-0.571034000	2.521929000	-1.194556000
C	0.842315000	1.083502000	0.103279000
C	1.564226000	-0.031626000	0.604620000
C	2.878543000	-0.619224000	0.091600000
C	3.845872000	-2.833440000	0.189369000
H	3.729075000	-3.744981000	0.776373000
H	4.855690000	-2.442853000	0.371381000
H	3.762849000	-3.097475000	-0.871410000
C	0.970679000	2.556229000	-0.031786000
C	1.269456000	3.191882000	1.314523000
H	1.301550000	4.277765000	1.203040000
H	2.232428000	2.845434000	1.695319000
H	0.493546000	2.941176000	2.040230000
C	2.068083000	2.883759000	-1.042636000
H	1.840545000	2.425832000	-2.008235000
H	3.053201000	2.549831000	-0.713953000
H	2.089579000	3.967841000	-1.171215000
C	3.019770000	-0.597471000	-1.413959000
H	4.008486000	-0.950844000	-1.714054000
H	2.905498000	0.417067000	-1.796408000
H	2.255139000	-1.211547000	-1.891942000
C	4.016523000	0.137086000	0.766188000
H	4.984760000	-0.291720000	0.499600000
H	3.908585000	0.095456000	1.852407000
H	4.029946000	1.184040000	0.457961000
C	-2.673274000	0.300146000	-0.176877000
C	-2.146233000	0.641976000	1.083500000
C	-1.592028000	-0.550465000	1.666341000
C	-1.929540000	-1.660713000	0.813882000
C	-2.558665000	-1.138170000	-0.324205000
C	-3.375404000	1.188682000	-1.138008000
H	-2.993360000	1.047059000	-2.152739000
H	-3.252819000	2.239719000	-0.871750000
H	-4.449488000	0.976374000	-1.162515000
C	-2.230026000	1.971542000	1.743466000
H	-1.989464000	2.785877000	1.060452000
H	-1.550344000	2.040298000	2.594460000
H	-3.242369000	2.130663000	2.127969000
C	-0.973552000	-0.662625000	3.013528000
H	-0.256840000	-1.485867000	3.051881000
H	-1.726687000	-0.844848000	3.786663000
H	-0.437724000	0.249817000	3.286809000
C	-1.642545000	-3.088363000	1.116122000
H	-1.616180000	-3.691884000	0.208580000
H	-2.407217000	-3.510361000	1.776030000
H	-0.677581000	-3.205276000	1.612865000
C	-3.086965000	-1.892793000	-1.486667000
H	-2.764568000	-1.440970000	-2.426808000
H	-4.181562000	-1.896063000	-1.468866000
H	-2.746727000	-2.928443000	-1.485718000
H	0.733029000	-1.336461000	-0.032029000
H	1.566328000	-0.143262000	1.697908000

C2

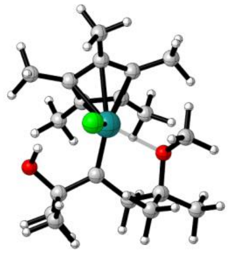

Ru	-0.357825000	-0.102775000	-0.132609000
Cl	-0.034710000	-0.315667000	-2.523698000
O	1.144019000	-1.841025000	0.131840000
O	0.482694000	2.948070000	-0.604465000
H	0.021721000	2.309376000	-1.171775000
C	1.240401000	0.822571000	0.251861000
C	2.319211000	0.015401000	0.923746000
H	2.017720000	-0.111396000	1.973803000
H	3.296227000	0.512270000	0.945328000
C	2.497477000	-1.366943000	0.301931000
C	0.964887000	-2.988802000	-0.674873000
H	-0.070281000	-3.304375000	-0.549387000
H	1.622369000	-3.800956000	-0.350281000
H	1.127513000	-2.766855000	-1.731847000
C	1.585250000	2.254672000	-0.057331000
C	2.021544000	3.042517000	1.168806000
H	2.179292000	4.083721000	0.879156000
H	2.954231000	2.658476000	1.584482000
H	1.255529000	3.016497000	1.944996000
C	2.699449000	2.255794000	-1.112120000
H	2.375775000	1.708092000	-2.000466000
H	3.635717000	1.826714000	-0.750602000
H	2.881320000	3.294044000	-1.396731000
C	3.221265000	-1.271010000	-1.028101000
H	3.435446000	-2.259205000	-1.439544000
H	4.178057000	-0.764548000	-0.883440000
H	2.630023000	-0.713799000	-1.758079000
C	3.232114000	-2.280941000	1.262493000
H	3.414287000	-3.266774000	0.830353000
H	2.659341000	-2.402116000	2.184816000
H	4.204482000	-1.850503000	1.513126000
C	-2.271749000	0.925376000	-0.162699000
C	-1.697145000	1.028894000	1.142161000
C	-1.544092000	-0.298365000	1.654220000
C	-2.177144000	-1.203504000	0.712793000
C	-2.633825000	-0.463204000	-0.374949000
C	-2.707669000	2.040986000	-1.041563000
H	-2.491732000	1.821771000	-2.089800000
H	-2.212185000	2.976593000	-0.780709000
H	-3.788278000	2.200219000	-0.956105000
C	-1.436585000	2.290228000	1.881109000
H	-1.064312000	3.074123000	1.221775000
H	-0.702765000	2.142549000	2.676154000
H	-2.360320000	2.642872000	2.351338000
C	-1.026991000	-0.694211000	2.990279000
H	-0.430483000	-1.608438000	2.927924000
H	-1.838048000	-0.880535000	3.703094000
H	-0.388054000	0.083250000	3.413712000
C	-2.289002000	-2.667395000	0.931633000
H	-2.545489000	-3.199525000	0.014584000
H	-3.063322000	-2.889229000	1.673118000
H	-1.353906000	-3.084087000	1.314643000
C	-3.301156000	-0.964934000	-1.601286000
H	-2.728590000	-0.696587000	-2.493468000
H	-4.301295000	-0.532052000	-1.700182000
H	-3.406760000	-2.050280000	-1.589962000

C2'

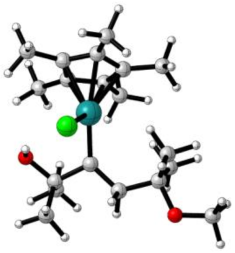

Ru	-0.646820000	-0.047856000	-0.222198000
Cl	-0.600555000	0.617569000	-2.525162000
O	4.311320000	-0.335217000	-0.249594000
O	-0.765532000	2.872609000	-0.066447000
H	-0.946050000	2.574445000	-0.971888000
C	0.890398000	1.089862000	0.056992000
C	2.334684000	0.811405000	-0.149034000
H	2.954142000	1.476400000	0.468671000
H	2.533758000	1.131692000	-1.185931000
C	2.925403000	-0.588014000	0.002150000
C	5.168180000	-1.436119000	-0.273775000
H	6.181524000	-1.048619000	-0.385194000
H	4.968574000	-2.109513000	-1.116978000
H	5.132257000	-2.024282000	0.652677000
C	0.597072000	2.574735000	0.208362000
C	0.800656000	2.930527000	1.674598000
H	0.568914000	3.988962000	1.813284000
H	1.828139000	2.751578000	1.995995000
H	0.125434000	2.344937000	2.301434000
C	1.445562000	3.473607000	-0.687739000
H	1.315303000	3.196423000	-1.736994000
H	2.508629000	3.440721000	-0.447635000
H	1.094805000	4.498991000	-0.559178000
C	2.736317000	-1.082177000	1.423508000
H	3.107507000	-2.100545000	1.561083000
H	1.676165000	-1.071082000	1.675973000
H	3.262894000	-0.422573000	2.118745000
C	2.364980000	-1.550066000	-1.025460000
H	2.734274000	-2.567305000	-0.872421000
H	2.631297000	-1.221256000	-2.033096000
H	1.272666000	-1.579016000	-0.965509000
C	-2.794320000	-0.434874000	-0.063962000
C	-2.229117000	-0.002264000	1.202488000
C	-1.304789000	-0.998499000	1.632824000
C	-1.215349000	-1.975000000	0.592312000
C	-2.188287000	-1.650371000	-0.423387000
C	-3.812093000	0.335476000	-0.819118000
H	-3.840774000	0.046191000	-1.869162000
H	-3.596018000	1.405282000	-0.780195000
H	-4.808704000	0.183750000	-0.392551000
C	-2.690896000	1.171768000	1.981976000
H	-2.751053000	2.064296000	1.358519000
H	-2.010734000	1.394397000	2.805538000
H	-3.681736000	0.981345000	2.408720000
C	-0.645691000	-1.047277000	2.965358000
H	0.153885000	-1.787573000	2.998192000
H	-1.372893000	-1.322262000	3.736076000
H	-0.216836000	-0.083659000	3.247729000
C	-0.416604000	-3.224844000	0.623243000
H	-0.139341000	-3.546412000	-0.381776000
H	-0.999347000	-4.034163000	1.076172000
H	0.500711000	-3.112130000	1.203132000
C	-2.392370000	-2.433588000	-1.668742000
H	-2.963296000	-1.870281000	-2.406193000
H	-2.926305000	-3.367105000	-1.465956000
H	-1.436989000	-2.689512000	-2.134062000

TS_{C2-E2}

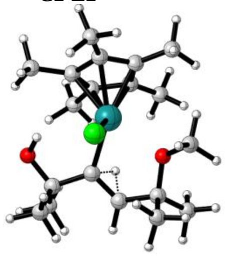

Ru	-0.457700000	-0.103521000	-0.137825000
Cl	0.198490000	-0.281061000	-2.485860000
O	1.139680000	-1.853844000	0.178591000
O	0.498458000	3.026451000	-0.257512000
H	-0.060190000	2.429105000	-0.780885000
C	1.418411000	0.832851000	0.130621000
C	2.506962000	0.035512000	0.452536000
H	1.386433000	0.307867000	1.274610000
H	3.494687000	0.458473000	0.659313000
C	2.506422000	-1.458426000	0.304815000
C	0.890967000	-3.082999000	-0.470383000
H	-0.128196000	-3.370585000	-0.219772000
H	1.568713000	-3.866301000	-0.116604000
H	0.967271000	-2.978100000	-1.556063000
C	1.693358000	2.318974000	-0.009165000
C	2.300809000	2.954107000	1.227182000
H	2.404444000	4.027878000	1.059513000
H	3.288461000	2.548221000	1.454052000
H	1.649739000	2.807603000	2.092454000
C	2.606147000	2.473548000	-1.224866000
H	2.157292000	1.978093000	-2.090395000
H	3.597608000	2.044402000	-1.057034000
H	2.716050000	3.536950000	-1.443708000
C	3.310988000	-1.727795000	-0.964196000
H	3.460164000	-2.798021000	-1.119625000
H	4.298421000	-1.269377000	-0.874798000
H	2.798776000	-1.298435000	-1.828829000
C	3.114404000	-2.141770000	1.516002000
H	3.157424000	-3.223941000	1.377912000
H	2.515945000	-1.928358000	2.405383000
H	4.133744000	-1.787398000	1.685760000
C	-2.214422000	1.098591000	0.004080000
C	-1.688819000	0.865401000	1.323786000
C	-1.692686000	-0.544203000	1.552442000
C	-2.295390000	-1.177610000	0.399507000
C	-2.610710000	-0.175153000	-0.538152000
C	-2.569909000	2.407602000	-0.608091000
H	-2.333043000	2.425845000	-1.675103000
H	-2.049947000	3.239303000	-0.132366000
H	-3.645892000	2.592048000	-0.514303000
C	-1.292828000	1.918987000	2.294685000
H	-0.823000000	2.763839000	1.788108000
H	-0.585510000	1.536680000	3.034917000
H	-2.165768000	2.291430000	2.840908000
C	-1.252975000	-1.255088000	2.782067000
H	-0.708195000	-2.170873000	2.535838000
H	-2.101039000	-1.537164000	3.415630000
H	-0.586812000	-0.632797000	3.383399000
C	-2.590674000	-2.627101000	0.260569000
H	-2.432950000	-2.973944000	-0.763322000
H	-3.632726000	-2.838786000	0.521614000
H	-1.967865000	-3.231869000	0.922920000
C	-3.229781000	-0.364269000	-1.873339000
H	-2.702900000	0.216530000	-2.632911000
H	-4.277225000	-0.045390000	-1.863526000
H	-3.196174000	-1.407788000	-2.188388000

TS$_{R1'-R3'}$

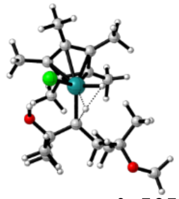

Ru	-0.737323000	-0.076599000	-0.575208000
Cl	-1.987524000	1.484264000	-1.918964000
O	4.447461000	-0.235211000	-0.753314000
O	-0.555796000	2.537114000	0.866705000
H	-1.170007000	2.467787000	0.116611000
C	0.931298000	0.952530000	-0.271693000
C	2.364075000	0.720085000	-0.723282000
H	2.998712000	1.567397000	-0.457461000
H	2.383000000	0.668155000	-1.820285000
C	3.143745000	-0.514682000	-0.239816000
C	5.434953000	-1.199779000	-0.540862000
H	6.365140000	-0.794266000	-0.939821000
H	5.225231000	-2.142579000	-1.061359000
H	5.591282000	-1.419965000	0.523187000
C	0.746996000	2.327364000	0.378337000
C	1.648835000	2.558970000	1.581907000
H	1.433240000	3.554374000	1.974247000
H	2.708321000	2.512156000	1.333880000
H	1.439600000	1.834140000	2.369300000
C	1.027122000	3.376550000	-0.709372000
H	0.364160000	3.223620000	-1.565346000
H	2.060250000	3.369997000	-1.056742000
H	0.809918000	4.356939000	-0.280351000
C	3.182922000	-0.551548000	1.277858000
H	3.622120000	-1.478101000	1.654042000
H	2.174115000	-0.470929000	1.685370000
H	3.768510000	0.290063000	1.654946000
C	2.603197000	-1.794576000	-0.840234000
H	3.083803000	-2.677331000	-0.411642000
H	2.768363000	-1.791879000	-1.920907000
H	1.529659000	-1.866586000	-0.668142000
C	-2.730242000	-1.030351000	-0.008630000
C	-2.443371000	-0.136862000	1.099661000
C	-1.270783000	-0.572429000	1.704865000
C	-0.778427000	-1.721339000	0.967837000
C	-1.742119000	-2.027920000	-0.046680000
C	-3.950745000	-0.961975000	-0.851063000
H	-3.806369000	-1.459565000	-1.810892000
H	-4.236123000	0.068785000	-1.060840000
H	-4.788261000	-1.449256000	-0.340475000
C	-3.319740000	0.992290000	1.493807000
H	-3.545236000	1.633964000	0.636872000
H	-2.863306000	1.609493000	2.266637000
H	-4.276565000	0.620475000	1.874380000
C	-0.649887000	-0.028898000	2.938187000
H	0.434295000	-0.159410000	2.938627000
H	-1.032242000	-0.553205000	3.820223000
H	-0.858417000	1.034393000	3.055939000
C	0.227556000	-2.689994000	1.480536000
H	0.608381000	-3.342605000	0.694457000
H	-0.236283000	-3.333275000	2.237031000
H	1.082530000	-2.207352000	1.952910000
C	-1.710456000	-3.220122000	-0.934571000
H	-2.236950000	-3.035354000	-1.871312000
H	-2.187655000	-4.076415000	-0.446920000
H	-0.688160000	-3.508495000	-1.183587000
H	0.400496000	0.564117000	-1.517705000
H	-0.118340000	-0.750521000	-1.891218000

XYZ coordinates (Å) for the molecules for the hydrogenation of 2-butyne using the cationic catalyst

A0

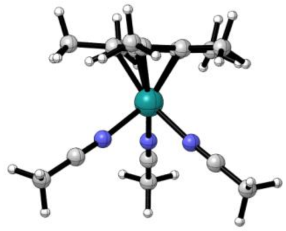

Ru	0.009857000	0.000049000	0.004442000
C	-1.709565000	-0.000267000	1.315973000
C	-1.736716000	-1.162118000	0.484941000
C	-1.825699000	-0.709270000	-0.882222000
C	-1.825704000	0.709896000	-0.881851000
C	-1.736648000	1.162029000	0.485533000
C	-1.662302000	-0.000645000	2.799114000
H	-1.152048000	0.882483000	3.186251000
H	-1.151961000	-0.883922000	3.185798000
H	-2.675649000	-0.000799000	3.210278000
C	-1.788935000	-2.576554000	0.936663000
H	-1.314316000	-2.703019000	1.910627000
H	-1.277799000	-3.237906000	0.234754000
H	-2.821444000	-2.928150000	1.024310000
C	-1.941642000	-1.587201000	-2.073642000
H	-1.522455000	-1.115732000	-2.963707000
H	-2.990506000	-1.816496000	-2.282997000
H	-1.426274000	-2.536802000	-1.922822000
C	-1.941666000	1.588439000	-2.072800000
H	-1.425850000	2.537759000	-1.921753000
H	-2.990511000	1.818259000	-2.281676000
H	-1.522975000	1.117237000	-2.963236000
C	-1.788777000	2.576234000	0.937993000
H	-1.313883000	2.702225000	1.911884000
H	-2.821259000	2.927796000	1.026087000
H	-1.277812000	3.237915000	0.236265000
C	2.084319000	-0.000275000	2.498625000
N	1.391430000	-0.000142000	1.584820000
C	2.948089000	-0.000437000	3.653324000
H	2.765049000	-0.886474000	4.262525000
H	3.994010000	-0.000012000	3.344693000
H	2.764509000	0.885042000	4.263174000
C	1.806315000	-2.299739000	-1.419371000
N	1.224980000	-1.456822000	-0.902604000
C	2.531320000	-3.362774000	-2.070651000
H	2.176727000	-3.491013000	-3.094093000
H	3.597051000	-3.133179000	-2.097843000
H	2.391155000	-4.301429000	-1.533129000
C	1.806587000	2.299816000	-1.419034000
N	1.225064000	1.456952000	-0.902391000
C	2.531908000	3.362672000	-2.070259000
H	2.255226000	3.419655000	-3.123733000
H	2.305879000	4.319723000	-1.598430000
H	3.605813000	3.185949000	-2.001487000

A1

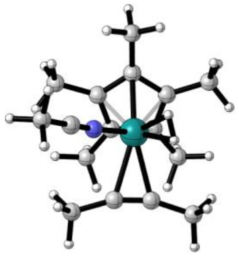

Ru	-0.033181000	0.081995000	-0.431147000
C	-0.558966000	2.225702000	0.291458000
C	0.254482000	2.372375000	-0.617860000
H	-0.196666000	-0.271098000	-2.158097000
H	-0.044591000	0.560590000	-2.160387000
C	1.139690000	2.975019000	-1.609078000
C	-1.544501000	2.474446000	1.335940000
C	0.159675000	-1.400132000	1.193425000
C	0.975321000	-0.279348000	1.502562000
C	1.958241000	-0.137020000	0.461160000
C	1.740460000	-1.175848000	-0.482600000
C	0.608140000	-1.955519000	-0.049771000
C	-0.955931000	-1.916813000	2.023993000
H	-1.680156000	-2.470015000	1.425416000
H	-1.483865000	-1.109273000	2.534627000
H	-0.577088000	-2.598961000	2.790239000
C	0.952394000	0.524009000	2.749651000
H	0.021812000	0.401649000	3.304115000
H	1.101588000	1.587411000	2.553835000
H	1.765477000	0.203286000	3.407866000
C	3.083907000	0.830163000	0.486471000
H	3.554966000	0.935631000	-0.491179000
H	3.856232000	0.490837000	1.183283000
H	2.762978000	1.818924000	0.819495000
C	2.566119000	-1.436394000	-1.689354000
H	1.997605000	-1.945541000	-2.468192000
H	3.418301000	-2.073025000	-1.435957000
H	2.960263000	-0.511293000	-2.112865000
C	0.095401000	-3.199218000	-0.682182000
H	-0.985891000	-3.290014000	-0.566570000
H	0.549584000	-4.085434000	-0.229557000
H	0.315029000	-3.227210000	-1.749836000
H	-1.409807000	1.814825000	2.194947000
H	-2.556864000	2.323316000	0.957254000
H	-1.470281000	3.507022000	1.684073000
H	2.117221000	2.489438000	-1.626613000
H	1.287345000	4.035097000	-1.391825000
H	0.714216000	2.891193000	-2.612078000
C	-3.244990000	-0.171978000	-0.630408000
N	-2.108476000	-0.037974000	-0.558642000
C	-4.672747000	-0.340361000	-0.731971000
H	-5.104675000	-0.517880000	0.253579000
H	-5.127773000	0.554434000	-1.158624000
H	-4.905842000	-1.190376000	-1.374623000

TS$_{A1-A3}$

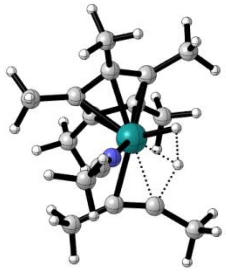

Ru	-0.076091000	0.098997000	-0.390660000
C	-0.850594000	1.906085000	0.525371000
C	-0.546215000	2.316088000	-0.621714000
H	-0.272496000	-0.142169000	-1.977136000
H	-0.175267000	1.179495000	-1.632272000
C	-0.385886000	3.406034000	-1.599424000
C	-1.466463000	2.086619000	1.839521000
C	0.634783000	-1.224975000	1.259845000
C	1.448547000	-0.064677000	1.297352000
C	2.099175000	0.077592000	0.031870000
C	1.755883000	-1.057539000	-0.764057000
C	0.818147000	-1.849849000	-0.029112000
C	-0.191936000	-1.769679000	2.367021000
H	-1.087493000	-2.264657000	1.988389000
H	-0.510044000	-0.988115000	3.058296000
H	0.368222000	-2.512258000	2.942543000
C	1.681149000	0.835040000	2.453425000
H	0.983500000	0.651461000	3.269826000
H	1.624008000	1.889076000	2.173713000
H	2.689394000	0.666754000	2.843227000
C	3.071075000	1.146901000	-0.312824000
H	3.191463000	1.247799000	-1.391834000
H	4.056571000	0.930444000	0.110096000
H	2.753730000	2.114516000	0.081015000
C	2.328680000	-1.385525000	-2.095075000
H	1.654348000	-2.005389000	-2.685465000
H	3.264606000	-1.937278000	-1.971200000
H	2.550407000	-0.488074000	-2.672856000
C	0.254083000	-3.162192000	-0.436594000
H	-0.754287000	-3.302831000	-0.044569000
H	0.868751000	-3.983858000	-0.057623000
H	0.204683000	-3.257906000	-1.521429000
H	-0.764936000	1.882439000	2.649619000
H	-2.307930000	1.399487000	1.960900000
H	-1.838050000	3.106476000	1.961833000
H	0.657105000	3.505001000	-1.909021000
H	-0.701137000	4.348499000	-1.149990000
H	-0.980383000	3.233385000	-2.498768000
C	-3.190611000	-0.704210000	-0.434868000
N	-2.087594000	-0.394762000	-0.417337000
C	-4.576209000	-1.099140000	-0.473979000
H	-4.862683000	-1.574998000	0.464456000
H	-5.210304000	-0.225939000	-0.631672000
H	-4.742259000	-1.803332000	-1.290218000

A3

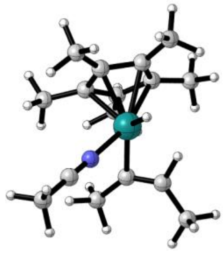

Ru	-0.054079000	0.002494000	-0.419890000
C	-1.041038000	1.626450000	0.395532000
C	-0.914507000	2.196712000	-0.772093000
H	-0.161150000	-0.652768000	-1.896378000
H	-0.164607000	1.696041000	-1.485230000
C	-1.643137000	3.314374000	-1.439779000
C	-1.821871000	1.879321000	1.611551000
C	0.852076000	-0.902093000	1.328038000
C	1.490818000	0.368540000	1.198655000
C	2.123963000	0.421831000	-0.074013000
C	1.957698000	-0.845832000	-0.714369000
C	1.155869000	-1.669483000	0.139538000
C	0.115281000	-1.401520000	2.515731000
H	-0.637371000	-2.139204000	2.235380000
H	-0.389844000	-0.592799000	3.045672000
H	0.798295000	-1.884772000	3.220422000
C	1.576089000	1.436352000	2.225081000
H	0.888328000	1.267523000	3.052751000
H	1.379588000	2.425339000	1.807211000
H	2.587773000	1.459014000	2.640418000
C	2.896790000	1.576940000	-0.592939000
H	2.989673000	1.546050000	-1.678620000
H	3.908116000	1.580774000	-0.175412000
H	2.430688000	2.525022000	-0.318048000
C	2.600798000	-1.253489000	-1.989651000
H	2.082744000	-2.091790000	-2.453558000
H	3.634618000	-1.558437000	-1.804312000
H	2.622295000	-0.435990000	-2.710357000
C	0.807588000	-3.095752000	-0.079028000
H	-0.154091000	-3.346310000	0.370170000
H	1.559753000	-3.748731000	0.372872000
H	0.751559000	-3.335201000	-1.140905000
H	-1.172263000	2.069411000	2.469821000
H	-2.441041000	1.014978000	1.866425000
H	-2.471630000	2.752265000	1.495509000
H	-0.955962000	4.083046000	-1.797609000
H	-2.345141000	3.775113000	-0.743977000
H	-2.202668000	2.952827000	-2.305244000
C	-2.982116000	-1.307593000	-0.362782000
N	-1.943918000	-0.824277000	-0.375540000
C	-4.285999000	-1.921317000	-0.365807000
H	-5.057769000	-1.160099000	-0.485554000
H	-4.363424000	-2.630502000	-1.190868000
H	-4.459375000	-2.451244000	0.571354000

TS$_{A3\text{-}E1}$

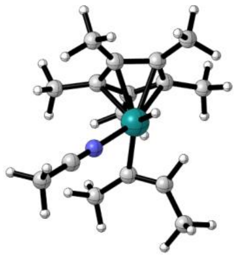

Ru	-0.055500000	0.000820000	-0.419910000
C	-1.022188000	1.627751000	0.386041000
C	-0.849028000	2.243075000	-0.758756000
H	-0.165090000	-0.647586000	-1.900881000
H	-0.054863000	1.799368000	-1.441378000
C	-1.602797000	3.356787000	-1.407033000
C	-1.854330000	1.858170000	1.572583000
C	0.838327000	-0.907940000	1.328317000
C	1.485757000	0.359427000	1.202606000
C	2.126979000	0.407868000	-0.065783000
C	1.954770000	-0.858802000	-0.707901000
C	1.145611000	-1.677849000	0.141866000
C	0.093664000	-1.403448000	2.512565000
H	-0.658120000	-2.140963000	2.229551000
H	-0.413469000	-0.592899000	3.037760000
H	0.772087000	-1.885745000	3.222382000
C	1.568197000	1.426438000	2.230265000
H	0.861529000	1.268805000	3.044187000
H	1.394530000	2.417918000	1.808426000
H	2.571570000	1.433850000	2.665801000
C	2.915101000	1.554515000	-0.580261000
H	2.996375000	1.534021000	-1.667252000
H	3.930797000	1.534125000	-0.173938000
H	2.471204000	2.508400000	-0.289552000
C	2.601229000	-1.268479000	-1.980740000
H	2.082908000	-2.105902000	-2.445895000
H	3.633890000	-1.575340000	-1.791982000
H	2.626652000	-0.451310000	-2.701719000
C	0.788442000	-3.101519000	-0.078977000
H	-0.178378000	-3.344936000	0.363057000
H	1.532337000	-3.759931000	0.378734000
H	0.738560000	-3.340833000	-1.141184000
H	-1.235697000	2.046660000	2.454382000
H	-2.471064000	0.984362000	1.797965000
H	-2.507627000	2.726826000	1.445218000
H	-0.935940000	4.167790000	-1.704985000
H	-2.353592000	3.759238000	-0.726431000
H	-2.109091000	3.008083000	-2.309781000
C	-2.987571000	-1.294589000	-0.376204000
N	-1.947528000	-0.814910000	-0.381192000
C	-4.293716000	-1.903334000	-0.389302000
H	-5.061569000	-1.139510000	-0.517464000
H	-4.366559000	-2.613844000	-1.213658000
H	-4.477395000	-2.430803000	0.547291000

E1

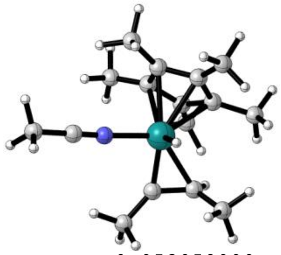

Ru	-0.053958000	0.323169000	-0.117549000
C	-0.319785000	2.118709000	0.408508000
C	1.013348000	2.188934000	0.009736000
H	-0.190299000	0.668253000	-1.677168000
H	1.774434000	2.060393000	0.780086000
C	1.456374000	2.993714000	-1.177099000
C	-1.369637000	3.105616000	0.645042000
C	0.203200000	-1.507119000	1.152890000
C	1.398719000	-0.749014000	1.220995000
C	1.950876000	-0.680611000	-0.107966000
C	1.129825000	-1.466482000	-0.973930000
C	0.040548000	-1.945500000	-0.217568000
C	-0.675909000	-1.897812000	2.284796000
H	-1.729335000	-1.885070000	2.000918000
H	-0.555540000	-1.230184000	3.137514000
H	-0.443699000	-2.912465000	2.621116000
C	2.033992000	-0.187207000	2.438184000
H	1.339771000	-0.148925000	3.276937000
H	2.420263000	0.822063000	2.277423000
H	2.887095000	-0.802280000	2.738405000
C	3.272697000	-0.118191000	-0.478153000
H	3.265751000	0.303623000	-1.484358000
H	4.027730000	-0.910103000	-0.464922000
H	3.603605000	0.656424000	0.213235000
C	1.413099000	-1.756267000	-2.401905000
H	0.504204000	-1.991124000	-2.955534000
H	2.083174000	-2.617087000	-2.484780000
H	1.898311000	-0.914233000	-2.895997000
C	-1.036331000	-2.836391000	-0.719826000
H	-1.915308000	-2.812882000	-0.075263000
H	-0.687860000	-3.872303000	-0.753079000
H	-1.346417000	-2.562914000	-1.729942000
H	-1.170330000	3.612320000	1.595418000
H	-2.373435000	2.686908000	0.679199000
H	-1.322471000	3.885466000	-0.124332000
H	1.877535000	3.950967000	-0.856163000
H	0.625194000	3.189352000	-1.856395000
H	2.232685000	2.472733000	-1.742015000
C	-3.249411000	0.055661000	-0.315578000
N	-2.115239000	0.182199000	-0.220560000
C	-4.675519000	-0.102361000	-0.444970000
H	-5.179943000	0.233409000	0.461748000
H	-5.041527000	0.480825000	-1.290970000
H	-4.919280000	-1.152086000	-0.613928000

TS_{E1-E2}

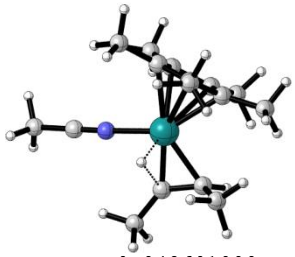

Ru	-0.042691000	0.347833000	-0.036848000
C	-0.242004000	2.217903000	0.237007000
C	1.146914000	2.104304000	0.373826000
H	-0.245075000	1.413103000	-1.296984000
H	1.519576000	2.140661000	1.400239000
C	2.098901000	2.641214000	-0.657781000
C	-1.218195000	3.306306000	0.241105000
H	-2.231876000	2.983724000	0.010977000
H	-0.920157000	4.093001000	-0.460514000
H	-1.211687000	3.764764000	1.235340000
H	1.655552000	2.633823000	-1.654847000
H	3.036376000	2.086256000	-0.696552000
H	2.357685000	3.676465000	-0.411395000
C	0.063577000	-1.769415000	-0.771754000
C	-0.119516000	-1.775095000	0.663609000
C	1.005956000	-1.165463000	1.260683000
C	1.893212000	-0.770955000	0.205211000
C	1.310934000	-1.156028000	-1.043528000
C	-0.843802000	-2.383098000	-1.774653000
H	-0.789180000	-1.863511000	-2.732046000
H	-1.884270000	-2.367323000	-1.448383000
H	-0.576782000	-3.428814000	-1.951192000
C	-1.251559000	-2.393725000	1.399195000
H	-2.121375000	-2.544867000	0.760247000
H	-1.556901000	-1.787072000	2.253371000
H	-0.957552000	-3.375034000	1.781311000
C	1.282651000	-1.008537000	2.709341000
H	1.704548000	-0.026567000	2.934478000
H	2.010692000	-1.753835000	3.044770000
H	0.381778000	-1.132613000	3.309678000
C	3.290122000	-0.319995000	0.415449000
H	3.732499000	0.101924000	-0.485712000
H	3.897988000	-1.185672000	0.696830000
H	3.379894000	0.408240000	1.222091000
C	1.937108000	-0.980917000	-2.377847000
H	1.187901000	-0.908878000	-3.166264000
H	2.584865000	-1.830418000	-2.612640000
H	2.549595000	-0.079412000	-2.417587000
C	-3.258895000	0.219681000	-0.126485000
N	-2.116561000	0.292291000	-0.087799000
C	-4.695438000	0.124278000	-0.174032000
H	-5.065976000	0.466079000	-1.141234000
H	-5.003018000	-0.911996000	-0.026900000
H	-5.140564000	0.734549000	0.612741000

E2

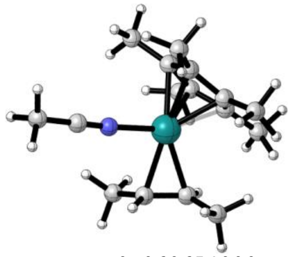

Ru	0.060654000	-0.226652000	-0.018922000
C	0.375509000	-2.447578000	0.092473000
C	-0.944332000	-2.257845000	-0.251792000
H	-1.684375000	-2.306256000	0.545267000
H	1.068836000	-2.712729000	-0.705239000
C	-1.440536000	-2.485928000	-1.643508000
C	0.830387000	-2.742088000	1.489916000
H	0.085976000	-2.433775000	2.227297000
H	1.777278000	-2.257786000	1.737172000
H	0.984462000	-3.819109000	1.610209000
H	-0.657564000	-2.299106000	-2.382247000
H	-2.300882000	-1.866211000	-1.895581000
H	-1.757842000	-3.527368000	-1.756598000
C	-0.058288000	1.731497000	0.856935000
C	-1.163474000	0.918814000	1.304240000
C	-1.967174000	0.588138000	0.172890000
C	-1.314674000	1.120233000	-0.982111000
C	-0.149989000	1.852880000	-0.552364000
C	0.981325000	2.307489000	1.744744000
H	1.867726000	2.609805000	1.187154000
H	1.292885000	1.591670000	2.508268000
H	0.598491000	3.191370000	2.262588000
C	-1.439163000	0.554760000	2.712686000
H	-0.515195000	0.404694000	3.273681000
H	-2.031777000	-0.358082000	2.785009000
H	-1.999004000	1.351986000	3.212218000
C	-3.299068000	-0.064526000	0.216668000
H	-3.527248000	-0.610400000	-0.698417000
H	-4.076628000	0.694110000	0.342253000
H	-3.389762000	-0.757756000	1.053351000
C	-1.782583000	1.049362000	-2.385949000
H	-0.971160000	0.774191000	-3.063241000
H	-2.153391000	2.027878000	-2.706771000
H	-2.590215000	0.330592000	-2.515813000
C	0.753372000	2.601524000	-1.459962000
H	1.709482000	2.826152000	-0.987152000
H	0.300503000	3.552325000	-1.755259000
H	0.952761000	2.037955000	-2.373797000
C	3.274967000	-0.228953000	-0.374764000
N	2.136837000	-0.274507000	-0.240706000
C	4.703766000	-0.174685000	-0.544254000
H	5.199695000	-0.364226000	0.408721000
H	5.024036000	-0.931763000	-1.261240000
H	5.006024000	0.807494000	-0.909393000

TS_{E1-C1}

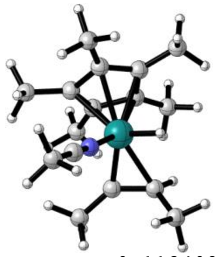

Ru	-0.113402000	0.217411000	-0.271894000
C	-0.481462000	1.890031000	0.517823000
C	-0.137050000	2.336872000	-0.752331000
H	-0.430193000	0.351510000	-1.842120000
H	0.891642000	2.650428000	-0.934201000
C	-1.170344000	3.010800000	-1.614929000
C	-1.015361000	2.442818000	1.752481000
H	-1.280383000	1.685266000	2.490688000
H	-1.907917000	3.029878000	1.496846000
H	-0.312993000	3.161623000	2.188989000
H	-2.171060000	2.630709000	-1.397554000
H	-0.970697000	2.836177000	-2.672455000
H	-1.170215000	4.092020000	-1.448175000
C	0.733300000	-1.271763000	1.215128000
C	1.607002000	-0.164492000	1.169195000
C	2.090743000	-0.028388000	-0.182416000
C	1.592063000	-1.134253000	-0.942284000
C	0.713259000	-1.864796000	-0.106995000
C	0.035564000	-1.817347000	2.407645000
H	-0.970848000	-2.161905000	2.163157000
H	-0.051572000	-1.073412000	3.200264000
H	0.577525000	-2.673892000	2.819448000
C	2.017315000	0.694002000	2.310019000
H	1.261748000	0.721929000	3.096017000
H	2.219917000	1.719700000	1.995909000
H	2.938307000	0.309902000	2.757485000
C	3.105739000	0.952296000	-0.644298000
H	3.011102000	1.154728000	-1.712094000
H	4.117097000	0.573867000	-0.469677000
H	3.025361000	1.903730000	-0.114898000
C	1.970096000	-1.461433000	-2.340437000
H	1.198144000	-2.042546000	-2.844243000
H	2.890128000	-2.052935000	-2.351002000
H	2.150392000	-0.562791000	-2.930387000
C	-0.023264000	-3.103394000	-0.471840000
H	-0.954411000	-3.196507000	0.088828000
H	0.577704000	-3.989244000	-0.247413000
H	-0.266840000	-3.130831000	-1.534541000
C	-3.175663000	-0.745859000	-0.168750000
N	-2.095577000	-0.363286000	-0.188653000
C	-4.535627000	-1.221403000	-0.156601000
H	-5.052172000	-0.905503000	-1.063948000
H	-4.550265000	-2.310950000	-0.108371000
H	-5.068624000	-0.826174000	0.708890000

C1

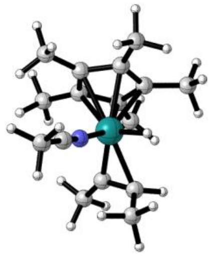

Ru	-0.133271000	0.261080000	-0.203896000
C	0.036709000	2.031654000	0.454886000
C	-0.443122000	2.308589000	-0.823881000
H	-0.628936000	0.321316000	-1.713602000
H	0.294645000	2.685254000	-1.539085000
C	-1.843519000	2.785939000	-1.096707000
C	0.682988000	2.803770000	1.509526000
H	1.031641000	2.210122000	2.353529000
H	-0.039293000	3.542015000	1.877813000
H	1.511339000	3.391796000	1.095663000
H	-2.529982000	2.488505000	-0.304010000
H	-2.222642000	2.399349000	-2.043594000
H	-1.855957000	3.877703000	-1.157362000
C	1.371181000	-0.774511000	1.227062000
C	2.041200000	-0.132185000	0.136915000
C	1.609768000	-0.747432000	-1.084298000
C	0.659979000	-1.764571000	-0.744512000
C	0.513826000	-1.760632000	0.677433000
C	1.597064000	-0.552014000	2.679502000
H	0.659330000	-0.455587000	3.230208000
H	2.191543000	0.341426000	2.869091000
H	2.140900000	-1.395880000	3.114204000
C	3.137340000	0.867881000	0.222970000
H	3.161468000	1.376677000	1.186204000
H	3.055469000	1.625353000	-0.559496000
H	4.104164000	0.373823000	0.091397000
C	2.176980000	-0.464224000	-2.427565000
H	1.510634000	-0.792842000	-3.224638000
H	3.132470000	-0.981485000	-2.555226000
H	2.361694000	0.601911000	-2.568053000
C	0.008508000	-2.720470000	-1.679624000
H	-1.030201000	-2.910497000	-1.403123000
H	0.527726000	-3.682973000	-1.676462000
H	0.011223000	-2.345278000	-2.702763000
C	-0.344002000	-2.677502000	1.468209000
H	-0.763434000	-2.180508000	2.344687000
H	0.243648000	-3.527611000	1.827253000
H	-1.165210000	-3.079842000	0.874414000
C	-3.236792000	-0.418361000	0.257027000
N	-2.140010000	-0.124848000	0.105134000
C	-4.620113000	-0.771743000	0.445770000
H	-4.985784000	-1.334837000	-0.413798000
H	-4.738658000	-1.378673000	1.344028000
H	-5.221773000	0.132044000	0.551954000

TS_{C1-C2}

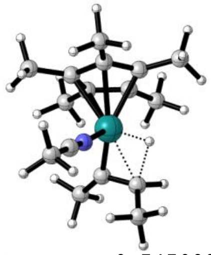

C	0.747988000	-1.414511000	1.100998000
C	1.721940000	-0.400251000	0.985336000
C	2.012620000	-0.220208000	-0.414957000
C	1.297517000	-1.229367000	-1.146091000
C	0.489193000	-1.927961000	-0.227949000
C	2.408838000	0.289031000	2.105554000
C	3.031679000	0.698271000	-0.986519000
C	1.427242000	-1.492025000	-2.601889000
C	-0.423990000	-3.058118000	-0.538757000
C	0.184204000	-1.973313000	2.356171000
H	1.761829000	0.404049000	2.976339000
H	2.776212000	1.274888000	1.820327000
H	3.276895000	-0.295937000	2.422794000
H	3.133744000	1.607760000	-0.391472000
H	2.773647000	0.995691000	-2.004380000
H	4.014657000	0.220012000	-1.025843000
H	0.534431000	-1.964583000	-3.011025000
H	2.271263000	-2.162157000	-2.788932000
H	1.608813000	-0.575523000	-3.163880000
H	-1.269969000	-3.092854000	0.149165000
H	0.102171000	-4.012691000	-0.447143000
H	-0.816746000	-2.995179000	-1.554336000
H	0.702113000	-2.895564000	2.636339000
H	-0.873790000	-2.219311000	2.250094000
H	0.280654000	-1.275176000	3.187626000
Ru	-0.116747000	0.266985000	-0.164065000
C	-0.427131000	2.280719000	-0.903429000
C	-1.806879000	2.797799000	-1.215699000
C	-0.020780000	2.056332000	0.422393000
C	0.449129000	2.932342000	1.489185000
H	-0.565895000	0.620384000	-1.661194000
H	0.342583000	2.638994000	-1.592365000
H	0.712265000	2.415400000	2.410674000
H	-0.344300000	3.658494000	1.705135000
H	1.302426000	3.529256000	1.143049000
H	-2.535102000	2.458717000	-0.478601000
H	-2.144393000	2.483553000	-2.203906000
H	-1.798251000	3.891196000	-1.200131000
C	-3.220302000	-0.494503000	0.218267000
N	-2.127213000	-0.176387000	0.087874000
C	-4.596644000	-0.885979000	0.382347000
H	-5.002106000	-0.463816000	1.302709000
H	-5.192420000	-0.534769000	-0.461069000
H	-4.671112000	-1.972994000	0.433999000

C2

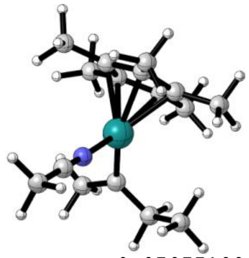

Ru	0.079771000	0.185618000	-0.136929000
C	-0.738745000	1.838674000	-0.686412000
C	-1.243840000	2.895313000	0.229459000
H	-2.338297000	2.760622000	0.253226000
H	-1.114995000	3.870652000	-0.261230000
C	-0.683592000	2.923090000	1.634729000
C	-1.078006000	2.097883000	-2.109851000
H	-2.133667000	2.381944000	-2.213565000
H	-0.858616000	1.269337000	-2.781877000
H	-0.503773000	2.968659000	-2.449720000
H	0.400692000	3.054421000	1.620084000
H	-0.896218000	1.993349000	2.166190000
H	-1.110340000	3.743854000	2.212169000
C	0.134832000	-1.979150000	-0.523045000
C	-1.148525000	-1.395096000	-0.880846000
C	-1.748055000	-0.891635000	0.309256000
C	-0.809845000	-1.100144000	1.375005000
C	0.319194000	-1.824398000	0.861126000
C	1.056017000	-2.618281000	-1.493242000
H	2.062115000	-2.724152000	-1.087296000
H	1.124949000	-2.036763000	-2.414956000
H	0.700458000	-3.616603000	-1.764676000
C	-1.757236000	-1.458586000	-2.229652000
H	-1.010568000	-1.319428000	-3.013738000
H	-2.533116000	-0.704057000	-2.362485000
H	-2.217874000	-2.438184000	-2.392099000
C	-3.109620000	-0.312059000	0.441109000
H	-3.153081000	0.444456000	1.226505000
H	-3.837679000	-1.087324000	0.694928000
H	-3.438579000	0.154841000	-0.488886000
C	-1.005497000	-0.758887000	2.802172000
H	-0.092429000	-0.358245000	3.246861000
H	-1.271721000	-1.658926000	3.366048000
H	-1.804177000	-0.032049000	2.947148000
C	1.492930000	-2.233673000	1.673662000
H	2.349301000	-2.487829000	1.048554000
H	1.259869000	-3.111044000	2.283296000
H	1.801590000	-1.438798000	2.356817000
C	3.231977000	0.902799000	-0.204553000
N	2.106451000	0.685682000	-0.194868000
C	4.642950000	1.185388000	-0.220493000
H	4.826876000	2.148255000	-0.698775000
H	5.029608000	1.218657000	0.798940000
H	5.174963000	0.409944000	-0.773037000

TS$_{C2-Z1}$

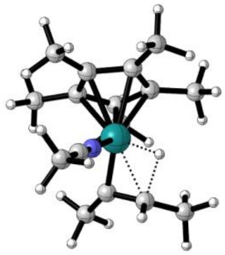

Ru	-0.101921000	-0.307200000	0.091615000
C	0.573527000	-1.884467000	-0.695208000
C	0.345304000	-2.381930000	0.602893000
H	-0.255991000	-1.063798000	1.511174000
C	1.497069000	-2.814040000	1.469617000
H	-0.576449000	-2.945661000	0.758370000
C	1.388301000	-2.371792000	-1.803935000
H	1.527007000	-1.640112000	-2.600113000
H	0.902946000	-3.256388000	-2.232258000
H	2.365998000	-2.715194000	-1.442429000
H	1.267557000	-2.705975000	2.530970000
H	2.391787000	-2.224132000	1.257108000
H	1.735142000	-3.865178000	1.285294000
C	1.691303000	0.938183000	-0.374025000
C	1.275379000	1.170577000	0.982596000
C	-0.015295000	1.779748000	0.951500000
C	-0.403515000	1.877533000	-0.420129000
C	0.652599000	1.386603000	-1.241625000
C	3.039996000	0.474295000	-0.790107000
H	3.027724000	-0.000818000	-1.771506000
H	3.469043000	-0.231364000	-0.076963000
H	3.725046000	1.324625000	-0.852679000
C	2.107854000	0.941979000	2.191426000
H	2.782096000	0.094090000	2.064879000
H	1.493852000	0.750694000	3.072107000
H	2.725074000	1.819971000	2.402395000
C	-0.790276000	2.276445000	2.119590000
H	-1.863255000	2.127837000	1.985609000
H	-0.627047000	3.347129000	2.272838000
H	-0.501892000	1.765166000	3.038244000
C	-1.670549000	2.466148000	-0.923000000
H	-2.030107000	1.947714000	-1.813366000
H	-1.518123000	3.514395000	-1.194906000
H	-2.457453000	2.437818000	-0.168744000
C	0.694502000	1.428099000	-2.725458000
H	1.098700000	2.383495000	-3.073838000
H	-0.298971000	1.317466000	-3.161117000
H	1.329023000	0.642778000	-3.138559000
C	-3.253346000	-0.907760000	-0.023906000
N	-2.124699000	-0.712712000	0.008785000
C	-4.671248000	-1.156030000	-0.069279000
H	-5.198814000	-0.254072000	-0.381947000
H	-4.888814000	-1.951681000	-0.783029000
H	-5.036305000	-1.454558000	0.914184000

Z1

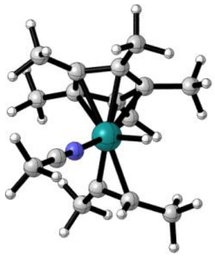

Ru	-0.126643000	0.285617000	-0.157897000
C	0.402033000	1.907718000	0.701034000
C	0.067711000	2.418829000	-0.544785000
H	-0.445049000	0.535067000	-1.696681000
H	-0.952025000	2.758236000	-0.718042000
C	1.106077000	3.025476000	-1.444320000
C	1.235481000	2.396959000	1.795521000
H	2.144385000	2.866725000	1.398904000
H	1.502927000	1.640874000	2.532308000
H	0.691077000	3.198769000	2.307139000
H	0.889515000	2.804935000	-2.491353000
H	2.102227000	2.635481000	-1.220827000
H	1.136817000	4.113402000	-1.334313000
C	-0.203066000	-1.887806000	0.536606000
C	0.843637000	-1.251600000	1.258309000
C	1.793386000	-0.765859000	0.307291000
C	1.332955000	-1.116806000	-1.004240000
C	0.095472000	-1.829498000	-0.856671000
C	-1.378552000	-2.553644000	1.150713000
H	-2.212270000	-2.627684000	0.451920000
H	-1.722306000	-2.022642000	2.040013000
H	-1.119984000	-3.570970000	1.458615000
C	0.953410000	-1.206907000	2.739601000
H	0.013771000	-0.910690000	3.209188000
H	1.728254000	-0.517020000	3.073179000
H	1.213678000	-2.194620000	3.131574000
C	3.121835000	-0.168537000	0.599239000
H	3.402892000	0.576570000	-0.146769000
H	3.890761000	-0.946275000	0.581637000
H	3.161659000	0.302292000	1.581082000
C	2.097620000	-0.915745000	-2.261885000
H	1.447860000	-0.938960000	-3.136455000
H	2.848564000	-1.702102000	-2.382306000
H	2.622075000	0.040758000	-2.264655000
C	-0.693378000	-2.473678000	-1.940479000
H	-1.767049000	-2.374636000	-1.771131000
H	-0.469642000	-3.542530000	-1.999832000
H	-0.473467000	-2.034251000	-2.913131000
C	-3.303729000	0.691074000	0.033976000
N	-2.165855000	0.570245000	-0.019599000
C	-4.734143000	0.845757000	0.101948000
H	-4.997214000	1.558547000	0.884493000
H	-5.120699000	1.209188000	-0.850985000
H	-5.203980000	-0.111735000	0.329938000

TS$_{Z1\text{-}Z2}$

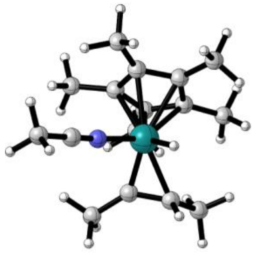

Ru	-0.053593000	0.259447000	-0.379402000
C	-0.060337000	2.109052000	-0.036307000
C	0.822047000	2.130722000	-1.112909000
H	-0.212537000	0.235723000	-1.978677000
H	0.406002000	2.488534000	-2.054609000
C	2.286139000	2.435729000	-0.958942000
C	-0.699734000	3.048848000	0.870725000
H	0.088222000	3.517699000	1.475922000
H	-1.425446000	2.580863000	1.537214000
H	-1.164846000	3.870622000	0.315332000
H	2.897265000	1.857814000	-1.653092000
H	2.639722000	2.242060000	0.054918000
H	2.462191000	3.493014000	-1.177621000
C	-0.216402000	-1.899542000	0.269510000
C	0.025466000	-1.071566000	1.435195000
C	1.302877000	-0.485401000	1.279973000
C	1.835224000	-0.906294000	0.008577000
C	0.909320000	-1.823986000	-0.578080000
C	-1.402185000	-2.770557000	0.061142000
H	-1.680839000	-2.828804000	-0.992348000
H	-2.267946000	-2.419430000	0.623397000
H	-1.189002000	-3.788001000	0.400466000
C	-0.862141000	-0.964541000	2.621894000
H	-1.916424000	-0.949371000	2.339577000
H	-0.658629000	-0.057017000	3.192050000
H	-0.721090000	-1.814179000	3.296664000
C	2.002847000	0.346777000	2.294004000
H	2.787552000	0.960319000	1.851817000
H	2.478636000	-0.291773000	3.043715000
H	1.314185000	1.008804000	2.822071000
C	3.214082000	-0.688147000	-0.497816000
H	3.226664000	-0.512644000	-1.574868000
H	3.819976000	-1.579425000	-0.308985000
H	3.710032000	0.150778000	-0.012838000
C	1.140001000	-2.594034000	-1.826318000
H	0.204488000	-2.914282000	-2.284528000
H	1.727641000	-3.491775000	-1.611555000
H	1.691654000	-2.010449000	-2.563495000
C	-3.253642000	0.350788000	-0.482483000
N	-2.108842000	0.353582000	-0.438476000
C	-4.692479000	0.341081000	-0.556053000
H	-5.066925000	-0.668108000	-0.378980000
H	-5.117795000	1.009020000	0.193710000
H	-5.019264000	0.665686000	-1.544902000

Z2

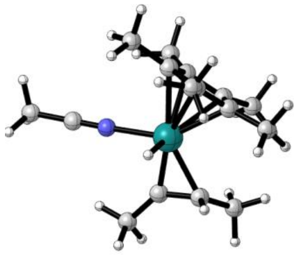

Ru	-0.053169000	0.331647000	-0.253010000
C	-0.286223000	2.194936000	-0.379778000
C	1.096147000	2.132500000	-0.552302000
H	-0.297173000	0.454303000	-1.826118000
C	1.994337000	2.737583000	0.489929000
H	1.489453000	2.174376000	-1.568953000
C	-1.301204000	3.242833000	-0.407502000
H	-2.300524000	2.905203000	-0.140741000
H	-1.330237000	3.709513000	-1.398175000
H	-0.996841000	4.034366000	0.287305000
H	3.002266000	2.322565000	0.466010000
H	1.588598000	2.617081000	1.495964000
H	2.093706000	3.811385000	0.297962000
C	-0.096034000	-1.932329000	-0.108449000
C	0.159335000	-1.389179000	1.212013000
C	1.392232000	-0.709773000	1.169446000
C	1.891543000	-0.789378000	-0.185664000
C	0.992502000	-1.599623000	-0.941246000
C	-1.238211000	-2.803138000	-0.487468000
H	-1.615865000	-2.561516000	-1.482880000
H	-2.064111000	-2.719743000	0.219234000
H	-0.929049000	-3.851865000	-0.500359000
C	-0.679656000	-1.620429000	2.414642000
H	-1.745298000	-1.583294000	2.183965000
H	-0.479596000	-0.882744000	3.191558000
H	-0.476185000	-2.608810000	2.837316000
C	2.101203000	-0.124934000	2.336083000
H	2.855338000	0.601503000	2.039586000
H	2.612467000	-0.915573000	2.893380000
H	1.414497000	0.367332000	3.026254000
C	3.229712000	-0.366625000	-0.668653000
H	3.179440000	0.067671000	-1.668474000
H	3.893123000	-1.234776000	-0.725444000
H	3.700279000	0.358590000	-0.007191000
C	1.200547000	-2.038421000	-2.343545000
H	0.258480000	-2.270931000	-2.839901000
H	1.818394000	-2.941216000	-2.364832000
H	1.713526000	-1.277991000	-2.932720000
C	-3.260824000	0.170307000	0.001732000
N	-2.121419000	0.260547000	-0.069816000
C	-4.694163000	0.053714000	0.086873000
H	-5.158301000	0.474914000	-0.805820000
H	-4.976524000	-0.997282000	0.163055000
H	-5.067935000	0.582356000	0.964487000

$TS_{Z2\text{-}Z3}$

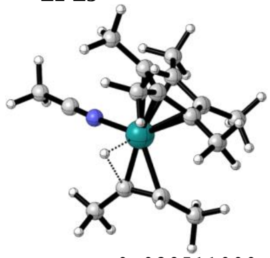

Ru	-0.038511000	0.311976000	-0.232662000
C	-0.257449000	2.171025000	-0.532089000
C	1.125896000	2.146425000	-0.386503000
H	-0.280803000	0.731273000	-1.782659000
C	1.772636000	2.864829000	0.761947000
H	1.742288000	2.056539000	-1.280605000
C	-1.279660000	3.198704000	-0.707275000
H	-2.299560000	2.831159000	-0.617516000
H	-1.160262000	3.691284000	-1.677784000
H	-1.112047000	3.974838000	0.048599000
H	2.750101000	2.446932000	1.007914000
H	1.151371000	2.837443000	1.659214000
H	1.939566000	3.914131000	0.496755000
C	-0.059481000	-1.941585000	-0.228921000
C	0.127390000	-1.474895000	1.130192000
C	1.344298000	-0.767667000	1.183697000
C	1.913164000	-0.764372000	-0.145874000
C	1.060373000	-1.535620000	-0.989947000
C	-1.170356000	-2.799639000	-0.714479000
H	-1.474191000	-2.528685000	-1.727174000
H	-2.047253000	-2.732080000	-0.070242000
H	-0.864539000	-3.849338000	-0.733148000
C	-0.757269000	-1.777653000	2.282912000
H	-1.802267000	-1.869962000	1.985304000
H	-0.693498000	-1.006774000	3.051398000
H	-0.469372000	-2.726904000	2.744121000
C	1.970134000	-0.210554000	2.408799000
H	2.703920000	0.560854000	2.181553000
H	2.489963000	-1.002627000	2.956194000
H	1.229289000	0.219557000	3.084358000
C	3.262192000	-0.277936000	-0.524940000
H	3.286613000	0.084976000	-1.553615000
H	3.987308000	-1.093995000	-0.453209000
H	3.610910000	0.524389000	0.124641000
C	1.320680000	-1.864692000	-2.413233000
H	0.396664000	-2.047952000	-2.961519000
H	1.932409000	-2.768871000	-2.483736000
H	1.860175000	-1.064611000	-2.920631000
C	-3.245307000	0.175950000	0.083900000
N	-2.109189000	0.275508000	-0.024074000
C	-4.673780000	0.046702000	0.219476000
H	-5.027832000	0.620903000	1.076458000
H	-5.171712000	0.409220000	-0.680694000
H	-4.939207000	-1.001342000	0.365670000

Z3

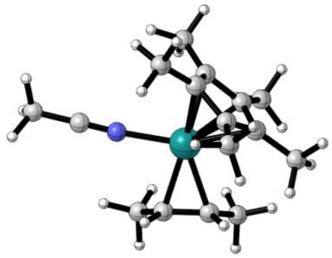

Ru	-0.054503000	0.192286000	-0.147491000
C	-0.377043000	2.301362000	-0.829059000
C	0.988999000	2.124707000	-0.721010000
H	-0.824517000	2.117020000	-1.805832000
C	1.804493000	2.791167000	0.342546000
H	1.523279000	1.840674000	-1.624913000
C	-1.177958000	3.161784000	0.103207000
H	-2.248284000	2.996355000	-0.013029000
H	-0.988218000	4.218075000	-0.112798000
H	-0.925813000	3.001902000	1.153183000
H	2.805647000	2.369856000	0.421010000
H	1.334799000	2.742081000	1.326339000
H	1.924502000	3.852370000	0.099037000
C	0.010470000	-1.963499000	-0.217597000
C	0.171582000	-1.529733000	1.122999000
C	1.368033000	-0.723633000	1.186403000
C	1.959454000	-0.693993000	-0.109610000
C	1.088640000	-1.406558000	-0.991227000
C	-1.065097000	-2.825223000	-0.767072000
H	-1.442337000	-2.435142000	-1.714836000
H	-1.906339000	-2.914121000	-0.079817000
H	-0.687222000	-3.833622000	-0.956223000
C	-0.695184000	-1.842484000	2.285119000
H	-1.679578000	-2.192806000	1.974779000
H	-0.835639000	-0.966512000	2.921430000
H	-0.244907000	-2.625299000	2.902309000
C	1.903358000	-0.122498000	2.429778000
H	2.668596000	0.625961000	2.228776000
H	2.349286000	-0.896932000	3.061655000
H	1.111662000	0.352536000	3.013258000
C	3.300204000	-0.177685000	-0.482570000
H	3.285995000	0.392943000	-1.412579000
H	3.981919000	-1.019116000	-0.635638000
H	3.733049000	0.450176000	0.294449000
C	1.317562000	-1.619859000	-2.437792000
H	0.383193000	-1.786623000	-2.974341000
H	1.949669000	-2.500369000	-2.594807000
H	1.825698000	-0.768520000	-2.892743000
C	-3.283223000	0.178563000	-0.080179000
N	-2.138660000	0.237853000	-0.117936000
C	-4.720704000	0.109574000	-0.033766000
H	-5.096062000	0.709748000	0.796288000
H	-5.143784000	0.493936000	-0.962822000
H	-5.047357000	-0.922067000	0.102009000

$TS_{A3\text{-}Z1}$

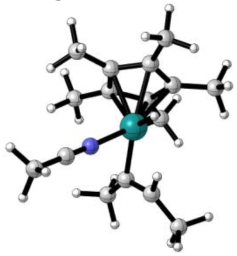

Ru	-0.101366000	0.024842000	-0.339399000
C	-1.128470000	1.643220000	0.397328000
C	-1.372775000	1.991186000	-0.847715000
H	-0.122514000	-0.599016000	-1.821038000
H	-1.290913000	1.175789000	-1.625853000
C	-1.613824000	3.338302000	-1.449623000
C	-1.309971000	2.329306000	1.683299000
C	1.058978000	-0.502877000	1.432044000
C	1.718802000	0.573300000	0.760279000
C	2.080252000	0.116685000	-0.550723000
C	1.719809000	-1.262166000	-0.660441000
C	1.033855000	-1.627367000	0.533157000
C	0.562185000	-0.530665000	2.831675000
H	-0.423748000	-0.995388000	2.896333000
H	0.483789000	0.469928000	3.255461000
H	1.238422000	-1.108870000	3.467898000
C	2.115428000	1.894241000	1.313297000
H	1.740555000	2.043743000	2.324854000
H	1.761111000	2.719876000	0.692165000
H	3.205393000	1.966468000	1.356707000
C	2.824962000	0.911645000	-1.558110000
H	2.612382000	0.574089000	-2.572885000
H	3.903978000	0.824067000	-1.398108000
H	2.569691000	1.970341000	-1.495608000
C	2.067436000	-2.174154000	-1.778928000
H	1.323537000	-2.959619000	-1.909871000
H	3.025225000	-2.659076000	-1.568710000
H	2.167068000	-1.641639000	-2.724220000
C	0.493874000	-2.971521000	0.851621000
H	-0.331107000	-2.911497000	1.562316000
H	1.269581000	-3.597566000	1.302305000
H	0.135719000	-3.482356000	-0.043016000
H	-0.361078000	2.524570000	2.186628000
H	-1.900150000	1.705939000	2.361412000
H	-1.837870000	3.279594000	1.562782000
H	-0.771874000	3.640142000	-2.077172000
H	-1.738929000	4.089946000	-0.669541000
H	-2.503589000	3.340616000	-2.081583000
C	-2.991368000	-1.410028000	-0.267110000
N	-1.975515000	-0.882436000	-0.293248000
C	-4.268991000	-2.075552000	-0.239554000
H	-5.021066000	-1.426062000	0.209569000
H	-4.582653000	-2.327891000	-1.253211000
H	-4.203348000	-2.993446000	0.345690000

TS$_{C2-R1}$

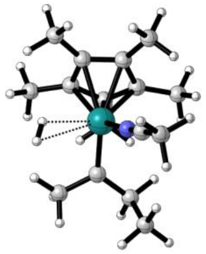

Ru	-0.059661000	-0.025859000	-0.296998000
H	-0.746329000	0.115236000	-3.072448000
H	-0.231131000	0.491453000	-2.668473000
C	-0.590049000	-1.859795000	-0.443995000
C	-1.182128000	-2.674803000	0.652971000
H	-0.962533000	-2.232071000	1.627970000
H	-0.781768000	-3.697697000	0.643450000
C	-2.701162000	-2.731305000	0.478191000
C	-0.279074000	-2.649810000	-1.663303000
H	0.497282000	-3.384661000	-1.409834000
H	0.066798000	-2.062477000	-2.513113000
H	-1.149783000	-3.243302000	-1.968439000
H	-2.981935000	-3.172052000	-0.479737000
H	-3.134413000	-1.731417000	0.533133000
H	-3.145038000	-3.339024000	1.266380000
C	1.618310000	1.470068000	-0.402837000
C	2.094838000	0.094114000	-0.447417000
C	1.761795000	-0.530467000	0.782422000
C	0.960989000	0.398705000	1.532507000
C	0.947890000	1.655339000	0.809118000
C	1.853875000	2.474374000	-1.470081000
H	1.132441000	3.290636000	-1.428139000
H	1.801113000	2.030497000	-2.465422000
H	2.851671000	2.911899000	-1.369052000
C	2.907408000	-0.480564000	-1.547859000
H	2.925287000	-1.569912000	-1.511079000
H	3.942257000	-0.129596000	-1.487651000
H	2.523720000	-0.185513000	-2.526489000
C	2.189368000	-1.877482000	1.238131000
H	1.463436000	-2.331153000	1.913688000
H	3.135932000	-1.806809000	1.780887000
H	2.343800000	-2.560588000	0.401707000
C	0.432116000	0.204074000	2.902971000
H	-0.528374000	0.705123000	3.033996000
H	1.122729000	0.622783000	3.642317000
H	0.297324000	-0.850843000	3.142732000
C	0.271861000	2.886581000	1.288355000
H	0.902735000	3.418309000	2.006099000
H	-0.666331000	2.654310000	1.796294000
H	0.051902000	3.573895000	0.470745000
C	-2.933153000	1.472844000	-0.380404000
N	-1.940089000	0.900554000	-0.352126000
C	-4.184705000	2.183398000	-0.418483000
H	-4.200166000	2.869910000	-1.266057000
H	-5.011700000	1.479754000	-0.522141000
H	-4.322301000	2.755632000	0.499757000

R1

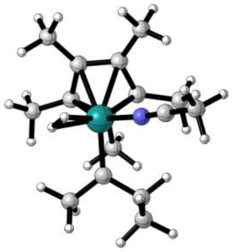

Ru	0.042732000	-0.044321000	-0.501194000
H	-0.216450000	0.070034000	-2.281735000
H	0.308855000	-0.585854000	-2.170574000
C	-0.877037000	-1.724685000	-0.289252000
C	-2.041084000	-1.905804000	0.624787000
H	-2.137140000	-1.058725000	1.308375000
H	-1.905463000	-2.812022000	1.230593000
C	-3.333998000	-2.053108000	-0.183298000
C	-0.398507000	-3.000783000	-0.873389000
H	0.031520000	-3.604350000	-0.061924000
H	0.353943000	-2.888518000	-1.652241000
H	-1.232061000	-3.598915000	-1.259107000
H	-3.329285000	-2.957150000	-0.792741000
H	-3.483559000	-1.202490000	-0.850413000
H	-4.188025000	-2.114165000	0.491078000
C	2.036103000	1.022376000	-0.444976000
C	2.176961000	-0.381071000	-0.088841000
C	1.476735000	-0.590811000	1.131724000
C	0.785243000	0.612648000	1.451806000
C	1.194763000	1.629133000	0.494557000
C	2.716165000	1.663577000	-1.599698000
H	2.251888000	2.611119000	-1.871882000
H	2.708128000	1.021301000	-2.481900000
H	3.764101000	1.864695000	-1.359113000
C	3.094151000	-1.341602000	-0.757022000
H	2.782076000	-2.375461000	-0.603764000
H	4.110376000	-1.243930000	-0.363467000
H	3.144950000	-1.167036000	-1.832591000
C	1.483074000	-1.830412000	1.948667000
H	0.516501000	-2.019059000	2.419205000
H	2.220126000	-1.740022000	2.751510000
H	1.755547000	-2.706726000	1.360255000
C	-0.026533000	0.870308000	2.668546000
H	-0.880894000	1.515011000	2.453421000
H	0.572245000	1.373559000	3.433843000
H	-0.406365000	-0.053705000	3.105649000
C	0.791813000	3.056363000	0.562948000
H	1.382709000	3.584041000	1.317358000
H	-0.256575000	3.168055000	0.845028000
H	0.939797000	3.567389000	-0.388327000
C	-2.728419000	1.631574000	-0.396865000
N	-1.775541000	0.996671000	-0.457340000
C	-3.934093000	2.418647000	-0.337356000
H	-3.988615000	3.090432000	-1.194951000
H	-4.807281000	1.764481000	-0.348229000
H	-3.954479000	3.013348000	0.576538000

TS_{R1-R3}

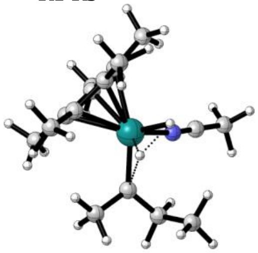

Ru	0.078774000	0.156012000	-0.307497000
C	1.010484000	1.793044000	0.250118000
C	2.488060000	1.989991000	0.259713000
H	3.008975000	1.104493000	0.622796000
H	2.746419000	2.824665000	0.921336000
C	2.973081000	2.312629000	-1.156011000
C	0.298347000	3.038599000	0.614072000
H	0.423096000	3.172922000	1.697594000
H	-0.763643000	3.043610000	0.382941000
H	0.775141000	3.915717000	0.159627000
H	2.430749000	3.161058000	-1.579037000
H	2.830055000	1.458607000	-1.818911000
H	4.031934000	2.569740000	-1.138498000
C	-1.898709000	-0.600590000	-0.988712000
C	-2.118126000	0.493436000	-0.075228000
C	-1.652518000	0.090484000	1.209389000
C	-1.087348000	-1.207151000	1.076791000
C	-1.267543000	-1.653324000	-0.279743000
C	-2.372576000	-0.629260000	-2.396440000
H	-1.812350000	-1.343045000	-2.999861000
H	-2.283954000	0.349092000	-2.869575000
H	-3.427405000	-0.916735000	-2.432209000
C	-2.931130000	1.698309000	-0.389704000
H	-2.841773000	2.468729000	0.376071000
H	-3.989549000	1.427980000	-0.448762000
H	-2.655779000	2.139527000	-1.348623000
C	-1.784214000	0.851187000	2.477545000
H	-0.944451000	0.669186000	3.149680000
H	-2.693844000	0.549136000	3.004996000
H	-1.854107000	1.925818000	2.307996000
C	-0.511232000	-2.018479000	2.179089000
H	0.218857000	-2.739582000	1.810156000
H	-1.298114000	-2.586361000	2.684202000
H	-0.023596000	-1.395704000	2.930158000
C	-0.925967000	-3.002148000	-0.800372000
H	-1.702499000	-3.728209000	-0.543749000
H	0.011724000	-3.371214000	-0.381434000
H	-0.823849000	-2.997231000	-1.885691000
C	2.743393000	-1.597520000	0.079168000
N	1.837461000	-0.905204000	-0.034870000
C	3.887814000	-2.462847000	0.212385000
H	4.126016000	-2.921400000	-0.748204000
H	4.753003000	-1.893258000	0.554113000
H	3.681576000	-3.251346000	0.937167000
H	0.565141000	-0.084549000	-1.813514000
H	0.073042000	1.342114000	-1.389140000

R3

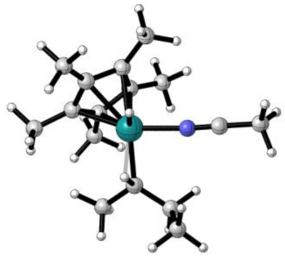

Ru	0.028170000	0.013257000	-0.362232000
C	1.081522000	1.730801000	-0.036876000
C	2.552315000	1.826368000	0.297667000
H	2.896004000	0.941378000	0.835429000
H	2.694800000	2.671841000	0.977918000
C	3.407731000	2.031761000	-0.944503000
C	0.393295000	3.044703000	0.164955000
H	0.356253000	3.284024000	1.231848000
H	-0.614214000	3.081994000	-0.245848000
H	0.975034000	3.850047000	-0.301808000
H	3.089917000	2.915651000	-1.503145000
H	3.334532000	1.172547000	-1.616379000
H	4.457117000	2.169131000	-0.681692000
C	-2.023242000	-0.705361000	-0.832070000
C	-2.166603000	0.567656000	-0.189878000
C	-1.632319000	0.463530000	1.122207000
C	-1.101151000	-0.855899000	1.278774000
C	-1.372273000	-1.595071000	0.073545000
C	-2.589285000	-1.037461000	-2.163967000
H	-2.137547000	-1.933862000	-2.587126000
H	-2.448105000	-0.222583000	-2.874640000
H	-3.665604000	-1.213434000	-2.076674000
C	-2.891952000	1.720743000	-0.781526000
H	-2.524906000	1.957779000	-1.782288000
H	-2.812218000	2.617973000	-0.168966000
H	-3.955865000	1.485172000	-0.878105000
C	-1.693855000	1.480527000	2.200353000
H	-0.792118000	1.477193000	2.814472000
H	-2.536726000	1.259009000	2.861878000
H	-1.839783000	2.488924000	1.815988000
C	-0.504976000	-1.406708000	2.520645000
H	0.149065000	-2.253255000	2.311472000
H	-1.291841000	-1.761696000	3.193252000
H	0.074039000	-0.655319000	3.059013000
C	-1.104255000	-3.041007000	-0.134413000
H	-1.031430000	-3.285995000	-1.194164000
H	-1.904785000	-3.649442000	0.295512000
H	-0.171590000	-3.347945000	0.340748000
C	2.734115000	-1.652593000	-0.036311000
N	1.790076000	-1.010140000	-0.132419000
C	3.930604000	-2.447574000	0.073745000
H	3.922497000	-3.246462000	-0.668799000
H	4.808677000	-1.822110000	-0.094472000
H	4.002381000	-2.890534000	1.067717000
H	0.143347000	-0.750677000	-1.761518000
H	0.935030000	1.401854000	-1.178579000

TS$_{R3\text{-}D1}$

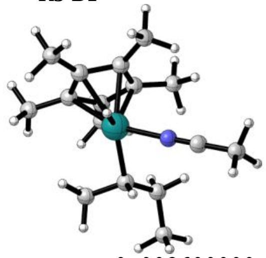

Ru	0.093600000	0.211192000	-0.570753000
C	-1.362152000	-1.338552000	-0.609908000
C	-2.297664000	-1.679342000	0.516068000
H	-2.688352000	-0.756490000	0.958573000
H	-1.742773000	-2.194716000	1.308115000
C	-3.457065000	-2.561801000	0.075527000
C	-0.658973000	-2.495070000	-1.265421000
H	-0.251578000	-3.203609000	-0.541419000
H	0.149940000	-2.166062000	-1.932554000
H	-1.351410000	-3.046275000	-1.913465000
H	-3.107627000	-3.535868000	-0.271969000
H	-4.014910000	-2.098457000	-0.742363000
H	-4.151946000	-2.737360000	0.897488000
C	2.237526000	0.634841000	-0.285890000
C	2.093570000	-0.767112000	-0.036017000
C	1.224544000	-0.945835000	1.070728000
C	0.753723000	0.340860000	1.471542000
C	1.418991000	1.337017000	0.653213000
C	3.195105000	1.221023000	-1.258774000
H	2.964923000	2.263188000	-1.476531000
H	3.193763000	0.675550000	-2.202879000
H	4.211503000	1.177693000	-0.856349000
C	2.840751000	-1.825079000	-0.758243000
H	2.827738000	-1.660439000	-1.837506000
H	2.444950000	-2.820296000	-0.560663000
H	3.890513000	-1.816881000	-0.448855000
C	0.983729000	-2.215450000	1.797237000
H	0.058751000	-2.193858000	2.371631000
H	1.799567000	-2.376596000	2.508747000
H	0.960341000	-3.083398000	1.139014000
C	-0.126597000	0.622176000	2.630933000
H	-0.614608000	1.592414000	2.538375000
H	0.458349000	0.638019000	3.555812000
H	-0.901023000	-0.138130000	2.744123000
C	1.348357000	2.804588000	0.856350000
H	1.613423000	3.348214000	-0.050187000
H	2.036852000	3.113694000	1.648436000
H	0.347565000	3.119853000	1.153805000
C	-2.505482000	2.068447000	-0.417279000
N	-1.587336000	1.385530000	-0.477198000
C	-3.653588000	2.936793000	-0.354695000
H	-4.115409000	3.014513000	-1.339905000
H	-4.389314000	2.544447000	0.348040000
H	-3.353382000	3.934136000	-0.031056000
H	0.423174000	1.367139000	-1.638058000
H	-1.914698000	-0.776609000	-1.387095000

D1

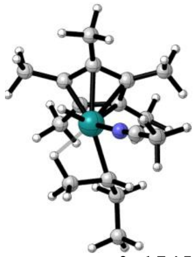

Ru	0.174548000	-0.032717000	-0.467718000
C	-0.297318000	2.048125000	-0.206872000
C	-1.541337000	2.568800000	0.453287000
H	-1.484245000	2.426796000	1.534436000
H	-2.417301000	2.012982000	0.100503000
C	-1.729817000	4.051218000	0.163106000
C	-0.350447000	1.884183000	-1.670435000
H	-1.337063000	2.077922000	-2.095508000
H	-0.176735000	0.776334000	-2.062079000
H	0.432227000	2.398771000	-2.226523000
H	-1.840157000	4.237609000	-0.907810000
H	-0.871495000	4.629762000	0.512452000
H	-2.618355000	4.441009000	0.661256000
C	-0.876681000	-1.929236000	-0.634395000
C	-1.742350000	-1.035305000	0.076550000
C	-1.118418000	-0.707409000	1.318511000
C	0.162821000	-1.317480000	1.339075000
C	0.312776000	-2.105768000	0.148446000
C	-1.236862000	-2.638820000	-1.888569000
H	-0.355840000	-2.994252000	-2.422133000
H	-1.799905000	-1.995052000	-2.565472000
H	-1.865415000	-3.505617000	-1.664148000
C	-3.119971000	-0.684357000	-0.357383000
H	-3.142741000	-0.355151000	-1.398434000
H	-3.555373000	0.105150000	0.253792000
H	-3.774561000	-1.557571000	-0.283224000
C	-1.718565000	0.008899000	2.469354000
H	-1.010332000	0.687896000	2.946799000
H	-2.017633000	-0.724075000	3.225186000
H	-2.606545000	0.575843000	2.197187000
C	1.133584000	-1.242933000	2.458672000
H	2.146943000	-1.471047000	2.127957000
H	0.872549000	-1.966964000	3.236383000
H	1.139673000	-0.254279000	2.920178000
C	1.444968000	-3.026098000	-0.133075000
H	1.486511000	-3.300643000	-1.186761000
H	1.346094000	-3.945889000	0.450146000
H	2.403198000	-2.574141000	0.127848000
C	3.188983000	1.035569000	-0.141349000
N	2.105131000	0.684828000	-0.262160000
C	4.555244000	1.469936000	0.004415000
H	5.215205000	0.842426000	-0.595798000
H	4.656967000	2.503504000	-0.328451000
H	4.862495000	1.407545000	1.048944000
H	0.907780000	-0.684730000	-1.733801000
H	0.588370000	2.575689000	0.145767000

TS_{D1-D3}

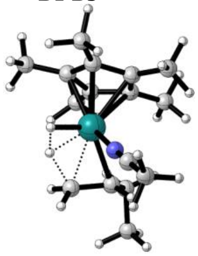

Ru	0.160460000	0.056286000	-0.482204000
C	-1.376337000	1.653387000	-0.251727000
C	-2.686521000	1.404584000	0.432716000
H	-2.548522000	1.310828000	1.511184000
H	-3.125077000	0.467403000	0.076534000
C	-3.646037000	2.552887000	0.152745000
C	-1.254958000	1.397812000	-1.636367000
H	-2.078411000	0.882978000	-2.128162000
H	-0.107741000	0.406213000	-2.100289000
H	-0.757779000	2.134934000	-2.262138000
H	-3.854165000	2.639267000	-0.916172000
H	-3.227636000	3.505213000	0.486516000
H	-4.595187000	2.407735000	0.669970000
C	0.167967000	-2.114578000	-0.599058000
C	-0.978636000	-1.736420000	0.160596000
C	-0.515654000	-1.107059000	1.366020000
C	0.899594000	-1.056250000	1.318832000
C	1.340753000	-1.677821000	0.107727000
C	0.133430000	-2.915461000	-1.849693000
H	1.027619000	-2.767795000	-2.455346000
H	-0.733688000	-2.665027000	-2.462136000
H	0.067674000	-3.980844000	-1.610490000
C	-2.372487000	-2.135094000	-0.171050000
H	-2.638189000	-1.866256000	-1.195679000
H	-3.101328000	-1.674924000	0.494621000
H	-2.486888000	-3.219262000	-0.082330000
C	-1.322143000	-0.734165000	2.555075000
H	-1.035012000	0.237842000	2.960159000
H	-1.159289000	-1.472290000	3.346265000
H	-2.389804000	-0.713163000	2.345837000
C	1.773597000	-0.503267000	2.382100000
H	2.753966000	-0.224723000	1.995042000
H	1.931949000	-1.248888000	3.166982000
H	1.328286000	0.375030000	2.852117000
C	2.756133000	-1.935448000	-0.266949000
H	2.861567000	-2.117863000	-1.336419000
H	3.143690000	-2.812314000	0.259203000
H	3.397770000	-1.090302000	-0.012278000
C	2.210860000	2.521882000	-0.154604000
N	1.456547000	1.668438000	-0.282191000
C	3.165150000	3.590318000	0.001605000
H	4.173517000	3.226140000	-0.198891000
H	2.940571000	4.398058000	-0.696000000
H	3.128576000	3.984700000	1.017697000
H	0.912270000	-0.041651000	-1.943067000
H	-0.853073000	2.531482000	0.121774000

D3

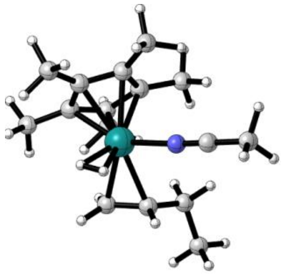

Ru	0.122401000	0.008458000	-0.564735000
C	-1.748248000	-1.305620000	-0.875971000
C	-2.530251000	-1.556503000	0.373973000
H	-2.907072000	-0.613407000	0.783668000
H	-1.876005000	-1.991368000	1.136016000
C	-3.694651000	-2.495668000	0.096319000
C	-0.626650000	-2.020950000	-1.232208000
H	-0.237349000	-2.776644000	-0.559000000
H	-0.147679000	0.450181000	-2.310625000
H	-0.345332000	-2.144703000	-2.271095000
H	-3.336867000	-3.460680000	-0.268522000
H	-4.360720000	-2.082194000	-0.664390000
H	-4.284797000	-2.671455000	0.996347000
C	2.100267000	-0.759167000	-0.052006000
C	1.233711000	-1.014178000	1.061205000
C	0.727385000	0.229965000	1.524517000
C	1.358243000	1.275684000	0.749147000
C	2.206518000	0.669315000	-0.203334000
C	2.914641000	-1.766390000	-0.780905000
H	3.120730000	-1.450694000	-1.804492000
H	2.416541000	-2.734856000	-0.830158000
H	3.877870000	-1.918221000	-0.285155000
C	1.004800000	-2.331843000	1.701051000
H	1.205274000	-3.163684000	1.026354000
H	-0.009420000	-2.438725000	2.087078000
H	1.684719000	-2.439190000	2.551405000
C	-0.158526000	0.439220000	2.697391000
H	-0.864166000	1.255078000	2.531118000
H	0.429514000	0.694513000	3.583895000
H	-0.734931000	-0.455682000	2.931843000
C	1.197719000	2.734483000	0.971852000
H	1.242961000	3.295416000	0.037145000
H	1.997561000	3.107774000	1.617778000
H	0.252749000	2.967433000	1.463065000
C	3.084868000	1.374970000	-1.168688000
H	3.230525000	0.798143000	-2.082616000
H	4.072366000	1.538739000	-0.727464000
H	2.682733000	2.350241000	-1.443956000
C	-2.225231000	2.232280000	-0.376390000
N	-1.420344000	1.421988000	-0.474867000
C	-3.232485000	3.256913000	-0.262957000
H	-3.455686000	3.671317000	-1.246991000
H	-4.148969000	2.843491000	0.159440000
H	-2.879467000	4.062159000	0.382579000
H	0.562369000	0.014865000	-2.288205000
H	-2.303369000	-0.808995000	-1.670636000

D4

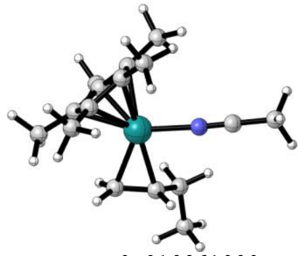

Ru	0.019861000	0.091405000	-0.174725000
C	1.693821000	1.445888000	-0.763549000
C	2.626120000	1.691338000	0.390533000
H	3.173307000	0.779300000	0.645717000
H	2.045201000	1.965432000	1.279262000
C	3.609175000	2.802756000	0.054240000
C	0.502812000	2.128459000	-0.898066000
H	0.209806000	2.852177000	-0.141827000
H	2.142629000	1.004937000	-1.653410000
H	0.031250000	2.244133000	-1.868749000
H	3.082065000	3.736554000	-0.151440000
H	4.195525000	2.552057000	-0.832608000
H	4.303122000	2.978970000	0.876872000
C	-1.976809000	-0.224604000	-0.892585000
C	-2.013148000	0.888288000	0.003449000
C	-1.523717000	0.435763000	1.264577000
C	-1.269166000	-0.982215000	1.171863000
C	-1.552217000	-1.389175000	-0.157255000
C	-2.387292000	-0.212958000	-2.314431000
H	-1.825334000	-0.941844000	-2.899494000
H	-2.247230000	0.768675000	-2.767720000
H	-3.448283000	-0.468073000	-2.404231000
C	-2.571980000	2.229311000	-0.297956000
H	-2.396545000	2.527925000	-1.331560000
H	-2.162544000	3.003130000	0.351046000
H	-3.654550000	2.217145000	-0.143524000
C	-1.349660000	1.251831000	2.487675000
H	-0.488752000	0.916547000	3.068668000
H	-2.230658000	1.169847000	3.132213000
H	-1.209444000	2.307502000	2.253820000
C	-0.791812000	-1.830045000	2.291562000
H	-0.377606000	-2.773574000	1.936483000
H	-1.613577000	-2.065098000	2.973798000
H	-0.019865000	-1.322168000	2.873115000
C	-1.446317000	-2.755534000	-0.725238000
H	-1.003190000	-2.738171000	-1.723006000
H	-2.436048000	-3.211535000	-0.815313000
H	-0.839250000	-3.409051000	-0.098899000
C	2.378107000	-2.122889000	-0.204772000
N	1.570862000	-1.308623000	-0.199398000
C	3.393390000	-3.143722000	-0.214053000
H	4.379678000	-2.692721000	-0.328976000
H	3.369544000	-3.706105000	0.720195000
H	3.223020000	-3.832399000	-1.042709000

TS$_{R3\text{-}B1}$

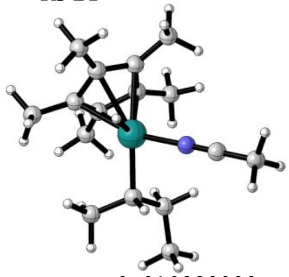

Ru	0.019289000	-0.130871000	-0.345402000
C	1.655810000	1.141312000	-0.659606000
C	2.489911000	1.283750000	0.611917000
H	2.717832000	0.295487000	1.027120000
H	1.900843000	1.806026000	1.378200000
C	3.790255000	2.043262000	0.397039000
C	1.268263000	2.465658000	-1.277515000
H	0.825778000	3.143213000	-0.542437000
H	0.561045000	2.346530000	-2.102001000
H	2.143119000	2.979689000	-1.692100000
H	3.609947000	3.082691000	0.119024000
H	4.381836000	1.584460000	-0.399390000
H	4.395537000	2.048069000	1.304780000
C	-2.302961000	-0.566282000	-0.379755000
C	-1.987438000	0.860371000	-0.426922000
C	-1.326526000	1.208570000	0.766723000
C	-1.070015000	-0.033833000	1.465664000
C	-1.749777000	-1.109197000	0.776816000
C	-3.137959000	-1.258594000	-1.391604000
H	-2.993452000	-2.338334000	-1.376195000
H	-2.936527000	-0.900016000	-2.401835000
H	-4.194799000	-1.062140000	-1.187863000
C	-2.453484000	1.772433000	-1.497364000
H	-2.233073000	1.366675000	-2.487413000
H	-1.985081000	2.753308000	-1.427049000
H	-3.537663000	1.910172000	-1.439311000
C	-1.053515000	2.566873000	1.297470000
H	-0.069484000	2.644140000	1.762823000
H	-1.792315000	2.812909000	2.066018000
H	-1.119756000	3.329623000	0.522884000
C	-0.382068000	-0.155164000	2.772115000
H	0.052521000	-1.146233000	2.908091000
H	-1.095883000	0.007182000	3.586993000
H	0.410359000	0.586867000	2.885286000
C	-1.796731000	-2.518483000	1.236308000
H	-2.084128000	-3.196104000	0.432611000
H	-2.519665000	-2.640382000	2.047942000
H	-0.827423000	-2.845494000	1.618018000
C	2.188882000	-2.465539000	-0.467114000
N	1.409982000	-1.626632000	-0.399793000
C	3.163021000	-3.522201000	-0.548725000
H	3.789695000	-3.521503000	0.344206000
H	2.666925000	-4.489992000	-0.630724000
H	3.800133000	-3.376240000	-1.422044000
H	-0.606793000	-0.871385000	-1.568408000
H	2.247883000	0.557900000	-1.383664000

B1

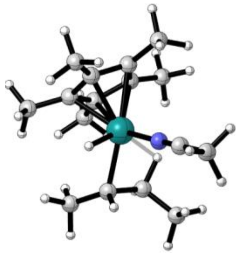

Ru	0.116755000	0.128134000	-0.107861000
C	0.661016000	2.089721000	-0.725431000
C	0.858296000	2.223716000	0.736770000
H	0.715251000	1.200122000	1.305523000
H	0.061017000	2.820900000	1.188158000
C	2.239601000	2.660258000	1.187857000
C	-0.368108000	2.956651000	-1.384261000
H	-1.305812000	2.969613000	-0.823693000
H	-0.582095000	2.623954000	-2.401392000
H	-0.015616000	3.991819000	-1.450214000
H	2.424322000	3.683010000	0.855584000
H	3.010673000	2.025416000	0.748813000
H	2.346730000	2.630920000	2.272437000
C	-1.064281000	-1.544191000	-0.775912000
C	-1.882884000	-0.351252000	-0.818878000
C	-1.994242000	0.134443000	0.507753000
C	-1.316737000	-0.801833000	1.383144000
C	-0.777910000	-1.838171000	0.602236000
C	-0.756459000	-2.443147000	-1.917387000
H	0.243102000	-2.871928000	-1.828872000
H	-0.807461000	-1.915426000	-2.869482000
H	-1.467783000	-3.273294000	-1.956593000
C	-2.561200000	0.193498000	-2.021308000
H	-1.983549000	0.013541000	-2.928415000
H	-2.729274000	1.267380000	-1.941939000
H	-3.536536000	-0.284152000	-2.149711000
C	-2.813216000	1.281268000	0.977674000
H	-2.291109000	1.881327000	1.725984000
H	-3.734240000	0.920838000	1.445423000
H	-3.100361000	1.939549000	0.158337000
C	-1.269417000	-0.680291000	2.862761000
H	-0.433703000	-1.231149000	3.294069000
H	-2.189146000	-1.080230000	3.300492000
H	-1.192674000	0.360156000	3.182567000
C	-0.029621000	-3.025071000	1.082544000
H	0.753139000	-3.315172000	0.380121000
H	-0.701252000	-3.882187000	1.186073000
H	0.432754000	-2.850086000	2.054203000
C	3.170391000	-0.853361000	-0.305266000
N	2.093273000	-0.473052000	-0.208991000
C	4.524408000	-1.329959000	-0.428261000
H	4.705817000	-1.685273000	-1.443477000
H	5.226667000	-0.524264000	-0.210201000
H	4.703538000	-2.148973000	0.269235000
H	0.331038000	0.284440000	-1.672609000
H	1.608607000	2.088950000	-1.265797000

TS$_{B1-B2}$

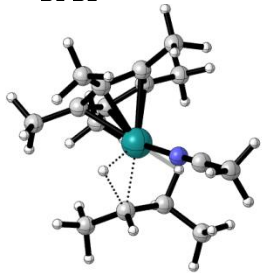

Ru	-0.104735000	0.128539000	0.074280000
C	-0.668729000	2.101224000	0.718302000
C	-0.843790000	2.233216000	-0.753125000
H	-0.698632000	1.219485000	-1.330018000
H	-0.040108000	2.835949000	-1.185058000
C	-2.217955000	2.687257000	-1.208713000
C	0.348941000	2.987769000	1.373046000
H	1.301970000	2.968389000	0.839088000
H	0.531835000	2.699648000	2.409530000
H	0.000638000	4.025925000	1.380183000
H	-2.406398000	3.701066000	-0.851325000
H	-2.995992000	2.040896000	-0.799419000
H	-2.309524000	2.687799000	-2.295069000
C	1.030512000	-1.535768000	0.814574000
C	1.869367000	-0.360703000	0.844341000
C	2.016433000	0.097620000	-0.490200000
C	1.338891000	-0.842566000	-1.357967000
C	0.758670000	-1.849035000	-0.565147000
C	0.679690000	-2.397256000	1.972286000
H	-0.314551000	-2.832247000	1.858647000
H	0.690301000	-1.836219000	2.906738000
H	1.390164000	-3.223236000	2.071162000
C	2.521244000	0.207165000	2.050935000
H	1.916585000	0.056578000	2.945918000
H	2.706998000	1.276362000	1.949572000
H	3.485813000	-0.279876000	2.218871000
C	2.854102000	1.229122000	-0.964656000
H	2.351788000	1.814043000	-1.738275000
H	3.785872000	0.856869000	-1.400660000
H	3.122245000	1.905321000	-0.153200000
C	1.314982000	-0.747751000	-2.839905000
H	0.479286000	-1.297044000	-3.273156000
H	2.236298000	-1.165541000	-3.257027000
H	1.253955000	0.287649000	-3.179085000
C	-0.003497000	-3.030418000	-1.037947000
H	-0.791156000	-3.305560000	-0.335225000
H	0.657777000	-3.896259000	-1.134516000
H	-0.462403000	-2.856238000	-2.011499000
C	-3.163512000	-0.849032000	0.290542000
N	-2.086748000	-0.467648000	0.191416000
C	-4.516539000	-1.328040000	0.416290000
H	-4.702185000	-1.665478000	1.436817000
H	-5.220866000	-0.529512000	0.179399000
H	-4.688950000	-2.160487000	-0.266882000
H	-0.349091000	0.546401000	1.594098000
H	-1.628973000	2.126327000	1.236208000

B2

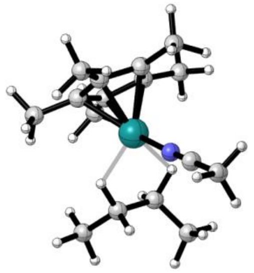

Ru	0.041363000	-0.012070000	-0.003341000
C	1.510542000	1.898441000	-0.756600000
C	1.448520000	1.938814000	0.771430000
H	1.092753000	0.997781000	1.298746000
H	0.738660000	2.708556000	1.086342000
C	2.818422000	2.196084000	1.371281000
C	1.164782000	3.242026000	-1.366701000
H	0.133684000	3.517475000	-1.128882000
H	1.274183000	3.235813000	-2.451183000
H	1.816654000	4.022738000	-0.968113000
H	3.249554000	3.108020000	0.951432000
H	3.498427000	1.371322000	1.145682000
H	2.772119000	2.314194000	2.453616000
C	-1.480353000	-1.194395000	-0.888805000
C	-1.881917000	0.187220000	-0.925915000
C	-1.961484000	0.674399000	0.409788000
C	-1.616741000	-0.411902000	1.287283000
C	-1.333695000	-1.564242000	0.491922000
C	-1.337368000	-2.097981000	-2.057005000
H	-0.586246000	-2.868149000	-1.876063000
H	-1.042659000	-1.551529000	-2.953593000
H	-2.281858000	-2.605126000	-2.275503000
C	-2.146194000	0.971519000	-2.156926000
H	-1.526984000	0.632750000	-2.989091000
H	-1.957657000	2.035599000	-2.006554000
H	-3.191072000	0.863558000	-2.462035000
C	-2.375606000	2.036047000	0.833796000
H	-1.897734000	2.328758000	1.770189000
H	-3.456535000	2.083807000	0.993808000
H	-2.125089000	2.787481000	0.083025000
C	-1.588006000	-0.344439000	2.768376000
H	-0.909527000	-1.084711000	3.192946000
H	-2.584532000	-0.536932000	3.177264000
H	-1.277149000	0.640290000	3.120819000
C	-0.977503000	-2.911499000	1.000774000
H	-0.385020000	-3.469411000	0.274746000
H	-1.878460000	-3.496472000	1.205988000
H	-0.404550000	-2.852134000	1.927267000
C	2.703389000	-1.840257000	-0.240201000
N	1.780731000	-1.163752000	-0.152872000
C	3.857482000	-2.694817000	-0.350860000
H	4.005030000	-2.992420000	-1.389915000
H	4.750111000	-2.171172000	-0.006513000
H	3.721361000	-3.591721000	0.254719000
H	0.831901000	1.164238000	-1.299676000
H	2.509908000	1.574632000	-1.062172000

TS$_{C2\text{-}R1'}$

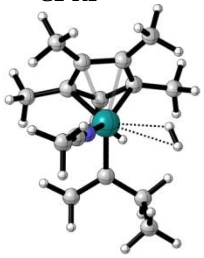

Ru	-0.125377000	0.059575000	-0.112671000
H	-1.323928000	0.727437000	-2.657817000
H	-0.795658000	0.234190000	-2.436995000
C	-0.644675000	1.835597000	0.378277000
C	-0.743548000	2.926580000	-0.639860000
H	-0.190263000	2.669536000	-1.547816000
H	-0.311786000	3.854401000	-0.239856000
C	-2.209342000	3.173689000	-1.000543000
C	-0.840104000	2.304684000	1.768948000
H	0.010677000	2.943058000	2.044897000
H	-0.928937000	1.502700000	2.500936000
H	-1.718911000	2.956454000	1.840511000
H	-2.791320000	3.485744000	-0.132240000
H	-2.671191000	2.270770000	-1.406922000
H	-2.284915000	3.959987000	-1.751229000
C	1.035009000	-1.799131000	0.343075000
C	1.303008000	-0.712267000	1.268877000
C	1.939627000	0.342001000	0.528373000
C	1.915947000	-0.029008000	-0.840622000
C	1.395711000	-1.383702000	-0.942247000
C	0.432052000	-3.096620000	0.736407000
H	-0.020922000	-3.608565000	-0.113182000
H	-0.336593000	-2.963060000	1.500260000
H	1.191534000	-3.761769000	1.156748000
C	1.146903000	-0.788983000	2.740244000
H	1.094436000	0.201027000	3.194490000
H	1.995233000	-1.314102000	3.191174000
H	0.242123000	-1.333111000	3.016121000
C	2.549439000	1.572628000	1.094047000
H	2.428207000	2.430739000	0.430134000
H	3.622336000	1.425473000	1.244849000
H	2.121029000	1.831947000	2.062567000
C	2.454755000	0.756893000	-1.977054000
H	1.846180000	0.637512000	-2.875046000
H	3.466691000	0.422718000	-2.226795000
H	2.507916000	1.820785000	-1.744216000
C	1.304438000	-2.157527000	-2.206234000
H	2.274458000	-2.595686000	-2.459620000
H	1.009331000	-1.528773000	-3.047822000
H	0.586211000	-2.974340000	-2.131854000
C	-3.017378000	-1.398285000	-0.005470000
N	-2.009442000	-0.852694000	-0.030424000
C	-4.286330000	-2.077349000	0.028478000
H	-5.095324000	-1.354248000	0.139358000
H	-4.438264000	-2.635496000	-0.896287000
H	-4.318744000	-2.772976000	0.867971000

R1'

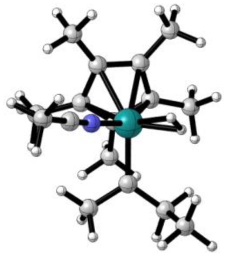

Ru	-0.067283000	0.083555000	-0.382775000
H	0.127004000	0.964843000	-1.913445000
H	-0.447841000	0.376483000	-2.119011000
C	-0.733538000	1.773065000	0.260516000
C	-0.322975000	3.104654000	-0.269587000
H	0.653956000	3.047051000	-0.757496000
H	-0.252030000	3.838756000	0.543815000
C	-1.359300000	3.596722000	-1.283705000
C	-1.611463000	1.870891000	1.448083000
H	-1.008199000	2.257819000	2.281223000
H	-2.058054000	0.926794000	1.756717000
H	-2.396551000	2.622037000	1.297681000
H	-2.349976000	3.683786000	-0.834589000
H	-1.432972000	2.919319000	-2.135835000
H	-1.075496000	4.580283000	-1.656808000
C	0.877082000	-1.882521000	0.205342000
C	0.740452000	-0.987698000	1.345948000
C	1.591608000	0.133285000	1.115783000
C	2.124241000	0.015609000	-0.197453000
C	1.716677000	-1.271654000	-0.733206000
C	0.240956000	-3.220278000	0.106085000
H	0.142213000	-3.548730000	-0.928805000
H	-0.751262000	-3.230774000	0.559902000
H	0.840759000	-3.967158000	0.634250000
C	0.022169000	-1.318449000	2.603632000
H	-0.182584000	-0.428604000	3.199761000
H	0.611714000	-2.001103000	3.223009000
H	-0.932564000	-1.808619000	2.403065000
C	1.904008000	1.221687000	2.076332000
H	2.001680000	2.189966000	1.582299000
H	2.857480000	1.016079000	2.570879000
H	1.149086000	1.310411000	2.857813000
C	3.120252000	0.923866000	-0.823946000
H	3.032261000	0.932570000	-1.911126000
H	4.137700000	0.600972000	-0.582976000
H	3.013193000	1.950371000	-0.471116000
C	2.167905000	-1.816909000	-2.039523000
H	3.183405000	-2.215008000	-1.956544000
H	2.187381000	-1.047812000	-2.813492000
H	1.524729000	-2.624745000	-2.387837000
C	-3.044932000	-1.194650000	-0.379797000
N	-2.009829000	-0.701931000	-0.386411000
C	-4.347025000	-1.812284000	-0.388137000
H	-4.381300000	-2.632588000	0.329685000
H	-5.111615000	-1.081772000	-0.121284000
H	-4.569555000	-2.205540000	-1.380943000

$TS_{R1'-R3'}$

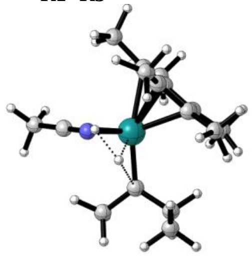

Ru	-0.085157000	0.173564000	-0.351617000
H	-0.439240000	0.308941000	-1.917406000
H	0.632742000	1.029379000	-1.534691000
C	0.484962000	2.015829000	0.031553000
C	1.813478000	2.374523000	0.597876000
H	2.219466000	1.576714000	1.219708000
H	1.699037000	3.264826000	1.229167000
C	2.807626000	2.695913000	-0.520904000
C	-0.318057000	3.215226000	-0.309326000
H	-0.834018000	3.510130000	0.614705000
H	-1.076707000	3.039907000	-1.068625000
H	0.301691000	4.071365000	-0.593740000
H	2.450775000	3.504344000	-1.160798000
H	2.984039000	1.820277000	-1.148072000
H	3.761079000	3.005235000	-0.093372000
C	-0.479662000	-2.040510000	-0.229542000
C	-0.395446000	-1.524792000	1.108913000
C	0.923015000	-1.041056000	1.327917000
C	1.647610000	-1.177813000	0.109987000
C	0.779749000	-1.817705000	-0.845288000
C	-1.650298000	-2.743811000	-0.816121000
H	-1.623094000	-2.726485000	-1.905770000
H	-2.591936000	-2.289329000	-0.503198000
H	-1.675904000	-3.791236000	-0.502670000
C	-1.458715000	-1.599434000	2.143209000
H	-1.461124000	-0.716229000	2.784452000
H	-1.300159000	-2.469123000	2.788024000
H	-2.449780000	-1.702969000	1.700920000
C	1.446849000	-0.570022000	2.635571000
H	2.426411000	-0.101377000	2.544428000
H	1.561815000	-1.418483000	3.316760000
H	0.772481000	0.140020000	3.118166000
C	3.102170000	-0.943827000	-0.088638000
H	3.333231000	-0.702517000	-1.126841000
H	3.668524000	-1.844051000	0.167572000
H	3.483303000	-0.134478000	0.535249000
C	1.190321000	-2.255047000	-2.205186000
H	1.697395000	-3.222689000	-2.153930000
H	1.881586000	-1.547791000	-2.664747000
H	0.334745000	-2.363974000	-2.871428000
C	-3.157202000	1.055726000	-0.149318000
N	-2.042850000	0.801565000	-0.231715000
C	-4.558814000	1.377448000	-0.055421000
H	-4.987597000	0.938243000	0.846124000
H	-4.694199000	2.458987000	-0.014470000
H	-5.093237000	0.990721000	-0.924059000

R3'

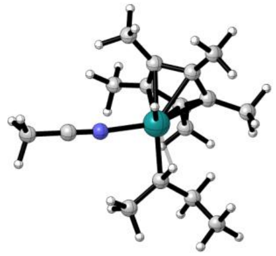

Ru	0.114016000	-0.073551000	-0.400834000
H	0.647931000	0.336816000	-1.845975000
H	-0.577093000	-1.565381000	-1.231031000
C	-0.517325000	-1.985787000	-0.107220000
C	-1.913009000	-2.312036000	0.354055000
H	-2.474109000	-1.408288000	0.587873000
H	-1.837899000	-2.894013000	1.280145000
C	-2.699367000	-3.100628000	-0.688149000
C	0.388047000	-3.177904000	-0.025040000
H	0.762008000	-3.262774000	1.000751000
H	1.244499000	-3.120061000	-0.692096000
H	-0.147018000	-4.108575000	-0.236424000
H	-2.202068000	-4.031217000	-0.965595000
H	-2.833177000	-2.508724000	-1.597603000
H	-3.689166000	-3.356442000	-0.309602000
C	0.517468000	2.037399000	-0.007301000
C	0.308809000	1.355022000	1.240715000
C	-1.052321000	0.920870000	1.296630000
C	-1.667935000	1.257354000	0.063066000
C	-0.704757000	1.956302000	-0.740219000
C	1.750591000	2.765021000	-0.402488000
H	1.811735000	2.890808000	-1.483481000
H	2.649014000	2.236157000	-0.081046000
H	1.776987000	3.759213000	0.052104000
C	1.284006000	1.245250000	2.353718000
H	1.215365000	0.279010000	2.856324000
H	1.088697000	2.018814000	3.102723000
H	2.309055000	1.378965000	2.008742000
C	-1.682625000	0.314628000	2.496488000
H	-2.674416000	-0.085770000	2.293594000
H	-1.793174000	1.077075000	3.273416000
H	-1.073004000	-0.487056000	2.917011000
C	-3.089607000	1.070054000	-0.327126000
H	-3.178210000	0.601756000	-1.309934000
H	-3.593851000	2.038843000	-0.387479000
H	-3.640168000	0.459852000	0.387612000
C	-1.012948000	2.595873000	-2.044488000
H	-1.547719000	3.536536000	-1.882635000
H	-1.649161000	1.960670000	-2.661772000
H	-0.110255000	2.818125000	-2.612386000
C	3.145203000	-1.060919000	-0.264013000
N	2.036997000	-0.775872000	-0.321956000
C	4.536110000	-1.431602000	-0.201456000
H	4.999138000	-1.016495000	0.694535000
H	4.632628000	-2.517826000	-0.172506000
H	5.065718000	-1.056067000	-1.077905000

TS$_{R3'-D1'}$

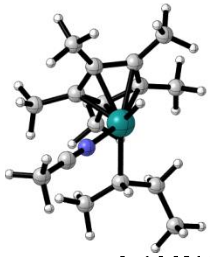

Ru	-0.106310000	-0.102817000	-0.508906000
H	-0.362160000	-0.986870000	-1.827331000
H	-1.399342000	1.855000000	-0.880004000
C	-0.661054000	1.900419000	-0.055629000
C	0.501727000	2.740791000	-0.520629000
H	1.053813000	2.207355000	-1.312528000
H	1.205602000	2.908097000	0.300162000
C	0.051157000	4.080654000	-1.092031000
C	-1.332368000	2.340255000	1.208189000
H	-0.614609000	2.442232000	2.026287000
H	-2.115568000	1.646241000	1.520994000
H	-1.803554000	3.322198000	1.087291000
H	-0.445171000	4.685398000	-0.330977000
H	-0.650484000	3.939722000	-1.917332000
H	0.900932000	4.650891000	-1.468496000
C	0.533884000	-1.968192000	0.286849000
C	0.407053000	-1.012007000	1.369955000
C	1.426702000	-0.025654000	1.210911000
C	2.116274000	-0.302203000	0.002715000
C	1.589309000	-1.507914000	-0.561366000
C	-0.215214000	-3.243212000	0.172171000
H	-0.222374000	-3.613076000	-0.852871000
H	-1.251466000	-3.129291000	0.492663000
H	0.239677000	-4.011165000	0.804836000
C	-0.508132000	-1.132090000	2.530349000
H	-0.853393000	-0.155687000	2.874440000
H	0.003934000	-1.614393000	3.368827000
H	-1.382809000	-1.737082000	2.291718000
C	1.799250000	0.989616000	2.224587000
H	2.243465000	1.882920000	1.787134000
H	2.545266000	0.558247000	2.899387000
H	0.951709000	1.290119000	2.839089000
C	3.274419000	0.440558000	-0.550728000
H	3.187070000	0.572554000	-1.630995000
H	4.196685000	-0.121262000	-0.373145000
H	3.392248000	1.423100000	-0.095979000
C	2.166635000	-2.206714000	-1.738378000
H	3.083171000	-2.730348000	-1.450769000
H	2.425631000	-1.507512000	-2.533952000
H	1.476899000	-2.940972000	-2.152628000
C	-3.274429000	-0.538453000	-0.452593000
N	-2.142815000	-0.359446000	-0.473812000
C	-4.696137000	-0.772456000	-0.440960000
H	-5.024148000	-1.067921000	0.556288000
H	-5.226715000	0.136075000	-0.728385000
H	-4.951435000	-1.564886000	-1.145641000

D1'

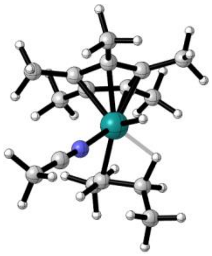

Ru	-0.119872000	0.116736000	-0.325741000
H	-0.536933000	-0.367785000	-1.793843000
H	-1.852899000	1.719585000	1.000162000
C	-0.842210000	1.904127000	0.636615000
C	-0.801623000	2.309441000	-0.778038000
H	-0.358093000	1.456791000	-1.497760000
H	-0.029913000	3.066548000	-0.949784000
C	-2.117779000	2.655973000	-1.442101000
C	-0.039153000	2.714607000	1.605141000
H	0.980563000	2.889026000	1.254551000
H	0.003585000	2.261155000	2.595012000
H	-0.505068000	3.698550000	1.731891000
H	-2.516467000	3.574545000	-1.008134000
H	-2.851929000	1.864432000	-1.286472000
H	-2.006085000	2.809597000	-2.515726000
C	0.973778000	-1.755477000	-0.452886000
C	0.800565000	-1.463744000	0.941981000
C	1.519856000	-0.280124000	1.250150000
C	2.075733000	0.225085000	0.035864000
C	1.770097000	-0.702844000	-1.014444000
C	0.512212000	-2.992382000	-1.135030000
H	0.503379000	-2.873562000	-2.218166000
H	-0.497184000	-3.268272000	-0.825850000
H	1.170160000	-3.832196000	-0.894736000
C	0.086827000	-2.314682000	1.925952000
H	-0.433081000	-1.714339000	2.674355000
H	0.796232000	-2.957117000	2.456148000
H	-0.642509000	-2.964668000	1.442459000
C	1.745186000	0.224909000	2.625423000
H	2.114286000	1.248279000	2.644229000
H	2.495865000	-0.402300000	3.116260000
H	0.841035000	0.175594000	3.234354000
C	2.973904000	1.399103000	-0.121869000
H	2.693468000	2.008715000	-0.983474000
H	4.003780000	1.068928000	-0.285567000
H	2.972755000	2.040743000	0.758455000
C	2.316905000	-0.631883000	-2.393882000
H	3.338284000	-1.023096000	-2.416042000
H	2.353911000	0.395688000	-2.757809000
H	1.721427000	-1.212799000	-3.097588000
C	-3.124253000	-0.923407000	0.138859000
N	-2.071322000	-0.504424000	-0.029737000
C	-4.449683000	-1.451718000	0.341529000
H	-4.403945000	-2.368018000	0.931293000
H	-5.067228000	-0.725923000	0.871766000
H	-4.915928000	-1.675420000	-0.618798000

TS$_{D1'-D3'}$

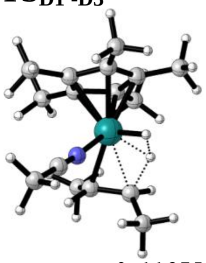

Ru	-0.112758000	0.149934000	-0.331552000
H	-0.559865000	-0.043653000	-1.893220000
H	-1.771276000	1.860859000	1.036365000
C	-0.772291000	2.050891000	0.646944000
C	-0.709581000	2.337529000	-0.734163000
H	-0.338444000	1.081136000	-1.680936000
H	0.159052000	2.919641000	-1.045560000
C	-1.960265000	2.607699000	-1.525862000
C	0.131891000	2.750795000	1.608968000
H	1.133440000	2.895049000	1.199828000
H	0.210524000	2.237151000	2.565761000
H	-0.274726000	3.745305000	1.820153000
H	-2.288858000	3.632156000	-1.334564000
H	-2.768333000	1.938730000	-1.227742000
H	-1.810110000	2.504578000	-2.601596000
C	0.912098000	-1.770936000	-0.510018000
C	0.711526000	-1.558152000	0.890454000
C	1.425094000	-0.399699000	1.281903000
C	2.060631000	0.146348000	0.115253000
C	1.767779000	-0.718363000	-0.982922000
C	0.429468000	-2.944116000	-1.285145000
H	0.428912000	-2.744156000	-2.356626000
H	-0.587489000	-3.220619000	-1.001769000
H	1.067532000	-3.814864000	-1.110040000
C	-0.063134000	-2.434890000	1.802514000
H	-0.583561000	-1.858581000	2.569222000
H	0.607363000	-3.129990000	2.316740000
H	-0.798175000	-3.032466000	1.262851000
C	1.607871000	0.029271000	2.691006000
H	2.057782000	1.016698000	2.772779000
H	2.273477000	-0.674901000	3.199415000
H	0.667297000	0.036538000	3.245010000
C	3.039385000	1.265149000	0.066135000
H	2.840988000	1.944935000	-0.764990000
H	4.051731000	0.874890000	-0.073145000
H	3.041372000	1.850370000	0.984754000
C	2.353363000	-0.596759000	-2.342940000
H	3.335278000	-1.077849000	-2.372850000
H	2.493021000	0.446878000	-2.627428000
H	1.729910000	-1.071461000	-3.100719000
C	-3.151448000	-0.790466000	0.162329000
N	-2.090677000	-0.397149000	-0.022027000
C	-4.485527000	-1.287612000	0.386401000
H	-5.082471000	-0.542270000	0.913206000
H	-4.967138000	-1.514490000	-0.565582000
H	-4.452323000	-2.196802000	0.987986000

D3'

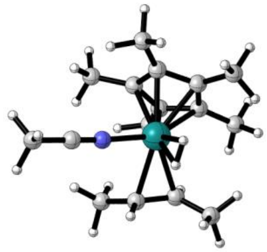

Ru	-0.019862000	0.144072000	-0.404219000
H	-0.222981000	0.754326000	-2.104625000
H	-1.195243000	2.641487000	-0.921747000
C	-0.547469000	2.375538000	-0.086648000
C	0.803236000	2.289718000	-0.357097000
H	-0.003171000	-0.044852000	-2.172001000
H	1.469516000	2.305538000	0.502074000
C	1.433809000	2.707793000	-1.645441000
C	-1.123659000	2.627744000	1.266744000
H	-0.433779000	2.341202000	2.061174000
H	-2.066608000	2.099007000	1.420888000
H	-1.335706000	3.694897000	1.387856000
H	1.879788000	3.700377000	-1.530058000
H	0.701343000	2.770422000	-2.453259000
H	2.233763000	2.032981000	-1.958240000
C	1.429030000	-1.527909000	-0.524485000
C	0.205012000	-1.976395000	0.054816000
C	0.013457000	-1.243142000	1.282778000
C	1.101302000	-0.345356000	1.434973000
C	1.971938000	-0.498740000	0.297094000
C	2.058813000	-2.052109000	-1.762268000
H	2.581559000	-1.267174000	-2.310891000
H	1.329052000	-2.506771000	-2.432433000
H	2.796237000	-2.819287000	-1.509263000
C	-0.650840000	-3.084541000	-0.441633000
H	-1.701439000	-2.922089000	-0.197880000
H	-0.355945000	-4.036639000	0.008710000
H	-0.576792000	-3.195793000	-1.523989000
C	-1.087061000	-1.446877000	2.257469000
H	-1.376262000	-0.510881000	2.738124000
H	-0.776668000	-2.140264000	3.044310000
H	-1.972833000	-1.869985000	1.783353000
C	1.397299000	0.463402000	2.643279000
H	1.856388000	1.425080000	2.410470000
H	2.107135000	-0.079447000	3.275010000
H	0.506057000	0.647453000	3.243086000
C	3.312563000	0.117971000	0.130724000
H	4.076856000	-0.511530000	0.596474000
H	3.379815000	1.101008000	0.597097000
H	3.582746000	0.227800000	-0.920477000
C	-3.248361000	-0.004702000	-0.558476000
N	-2.106010000	0.087271000	-0.524898000
C	-4.683980000	-0.123025000	-0.609415000
H	-5.032161000	-0.816309000	0.157238000
H	-5.149364000	0.848524000	-0.439501000
H	-4.995849000	-0.495361000	-1.586075000

D4'

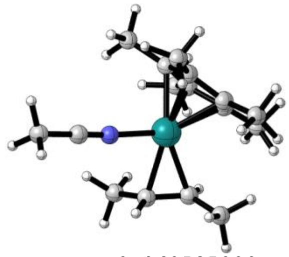

Ru	-0.060535000	0.226707000	-0.019224000
H	-1.067533000	2.712733000	-0.706792000
C	-0.374739000	2.447692000	0.091417000
C	0.945308000	2.257622000	-0.251844000
H	1.684771000	2.305959000	0.545759000
C	1.442677000	2.485249000	-1.643211000
C	-0.830347000	2.742779000	1.488536000
H	-0.086379000	2.434651000	2.226439000
H	-1.777352000	2.258597000	1.735573000
H	-0.984322000	3.819865000	1.608354000
H	1.760529000	3.526534000	-1.756199000
H	0.660194000	2.298685000	-2.382525000
H	2.302957000	1.865119000	-1.894526000
C	1.315423000	-1.120459000	-0.981141000
C	0.150086000	-1.852846000	-0.552692000
C	0.056964000	-1.731554000	0.856521000
C	1.161884000	-0.919212000	1.305059000
C	1.966844000	-0.588614000	0.174567000
C	1.784900000	-1.049788000	-2.384472000
H	2.593356000	-0.331773000	-2.513356000
H	0.974467000	-0.773788000	-3.062612000
H	2.155125000	-2.028610000	-2.705027000
C	-0.752436000	-2.601177000	-1.461338000
H	-1.709191000	-2.825581000	-0.989778000
H	-0.299430000	-3.552042000	-1.756218000
H	-0.950722000	-2.037488000	-2.375335000
C	-0.983867000	-2.307111000	1.743205000
H	-1.297315000	-1.590546000	2.505262000
H	-0.601505000	-3.190163000	2.262799000
H	-1.869020000	-2.610626000	1.184252000
C	1.436078000	-0.555299000	2.713831000
H	2.028744000	0.357453000	2.786868000
H	1.995247000	-1.352640000	3.213933000
H	0.511495000	-0.405131000	3.273785000
C	3.298812000	0.063801000	0.219742000
H	4.076214000	-0.695066000	0.344885000
H	3.389130000	0.756081000	1.057250000
H	3.527519000	0.610681000	-0.694612000
C	-3.275024000	0.229425000	-0.374206000
N	-2.136743000	0.274970000	-0.241435000
C	-4.703779000	0.175435000	-0.544267000
H	-5.060078000	-0.850487000	-0.445127000
H	-5.194597000	0.794294000	0.207960000
H	-4.974276000	0.547731000	-1.533380000

$TS_{R3'-B1'}$

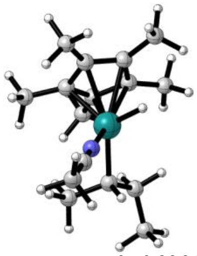

Ru	0.062296000	0.172605000	-0.255429000
H	-0.517061000	0.520123000	-1.662441000
H	2.564944000	0.192889000	-0.794727000
C	2.049244000	-0.480986000	-0.091330000
C	2.250750000	-1.914077000	-0.549656000
H	1.684602000	-2.091343000	-1.471640000
H	1.847021000	-2.604311000	0.199052000
C	3.712196000	-2.253618000	-0.807113000
C	2.584638000	-0.210598000	1.306007000
H	2.098954000	-0.850123000	2.050858000
H	2.449896000	0.827532000	1.619199000
H	3.656608000	-0.425103000	1.367533000
H	4.310424000	-2.189456000	0.103548000
H	4.148315000	-1.570567000	-1.540410000
H	3.817978000	-3.267430000	-1.195853000
C	-2.107363000	0.603251000	0.408389000
C	-1.271417000	-0.089631000	1.364930000
C	-0.979638000	-1.415304000	0.859864000
C	-1.468705000	-1.451427000	-0.460007000
C	-2.222596000	-0.223951000	-0.706053000
C	-2.687929000	1.953544000	0.605639000
H	-3.006593000	2.397755000	-0.337182000
H	-1.968902000	2.632473000	1.068363000
H	-3.560037000	1.911007000	1.264323000
C	-0.927980000	0.404560000	2.718664000
H	0.023657000	0.001009000	3.069509000
H	-1.695238000	0.093844000	3.436078000
H	-0.871522000	1.493341000	2.747841000
C	-0.406928000	-2.530824000	1.652908000
H	-0.085524000	-3.358271000	1.021990000
H	-1.166834000	-2.915949000	2.339508000
H	0.444275000	-2.219699000	2.260473000
C	-1.399471000	-2.582461000	-1.414264000
H	-1.137566000	-2.238757000	-2.417548000
H	-2.369835000	-3.083282000	-1.487737000
H	-0.659625000	-3.322602000	-1.111925000
C	-3.018815000	0.021285000	-1.933348000
H	-3.975972000	-0.503199000	-1.857430000
H	-2.519027000	-0.355933000	-2.826435000
H	-3.232391000	1.079309000	-2.081339000
C	1.359407000	3.079569000	-0.444772000
N	0.888326000	2.038091000	-0.353253000
C	1.944924000	4.389660000	-0.558449000
H	2.286529000	4.731835000	0.419448000
H	2.799378000	4.360024000	-1.235859000
H	1.212316000	5.099671000	-0.944016000

B1'

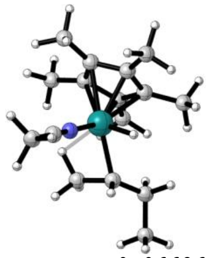

Ru	-0.066267000	0.194692000	0.041908000
H	0.255390000	0.774802000	-1.399724000
H	1.179469000	2.571800000	-0.417824000
C	1.504287000	1.620533000	0.004421000
C	2.864270000	1.223226000	-0.496387000
H	2.818552000	1.053100000	-1.576659000
H	3.159294000	0.270514000	-0.041839000
C	3.908351000	2.284280000	-0.188565000
C	1.254197000	1.528876000	1.462664000
H	2.069293000	1.058404000	2.014385000
H	0.333418000	0.871475000	1.760238000
H	0.948324000	2.468299000	1.925393000
H	4.004419000	2.446565000	0.888052000
H	3.640377000	3.240296000	-0.644218000
H	4.889689000	1.998850000	-0.568906000
C	-1.520482000	-1.550328000	0.128004000
C	-0.511636000	-1.769862000	1.081054000
C	0.768823000	-1.805463000	0.400988000
C	0.531364000	-1.627808000	-0.984481000
C	-0.887528000	-1.395695000	-1.153825000
C	-2.981377000	-1.468746000	0.369591000
H	-3.450242000	-0.718720000	-0.269260000
H	-3.210819000	-1.223621000	1.406633000
H	-3.458407000	-2.426633000	0.142920000
C	-0.690886000	-1.988577000	2.539310000
H	0.126599000	-1.556559000	3.118844000
H	-0.702838000	-3.060692000	2.758006000
H	-1.626841000	-1.567708000	2.906263000
C	2.056357000	-2.154879000	1.054394000
H	2.908175000	-1.967066000	0.401555000
H	2.071522000	-3.216994000	1.316379000
H	2.212020000	-1.596633000	1.980112000
C	1.526517000	-1.755135000	-2.078106000
H	1.274175000	-1.123408000	-2.930234000
H	1.563502000	-2.789168000	-2.432154000
H	2.529442000	-1.484166000	-1.748756000
C	-1.608901000	-1.249635000	-2.443720000
H	-2.011568000	-2.212829000	-2.770568000
H	-0.953481000	-0.878019000	-3.231077000
H	-2.447310000	-0.556822000	-2.355049000
C	-2.199541000	2.601797000	0.000541000
N	-1.433744000	1.749796000	0.038668000
C	-3.166529000	3.668435000	-0.053214000
H	-4.120397000	3.336045000	0.357927000
H	-2.814825000	4.523621000	0.525054000
H	-3.318672000	3.984145000	-1.086157000

TS$_{B1'-B2'}$

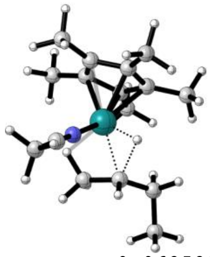

Ru	0.062522000	0.178850000	-0.069711000
H	-0.470531000	0.873649000	1.263913000
H	-1.228958000	2.585293000	0.396106000
C	-1.530974000	1.614774000	0.000560000
C	-2.891429000	1.210499000	0.501344000
H	-2.856046000	1.068781000	1.586069000
H	-3.164745000	0.241057000	0.068942000
C	-3.946141000	2.247641000	0.154444000
C	-1.292232000	1.508845000	-1.463799000
H	-2.112053000	1.026597000	-1.997829000
H	-0.369308000	0.864888000	-1.775079000
H	-1.014963000	2.453457000	-1.933620000
H	-4.035250000	2.379810000	-0.926704000
H	-3.696734000	3.219234000	0.587190000
H	-4.926541000	1.957903000	0.533524000
C	1.544903000	-1.511945000	-0.107718000
C	0.540919000	-1.790172000	-1.052804000
C	-0.735230000	-1.842707000	-0.370760000
C	-0.498267000	-1.623678000	1.010460000
C	0.910030000	-1.348830000	1.175253000
C	3.002504000	-1.397655000	-0.357387000
H	3.458872000	-0.639102000	0.280160000
H	3.219700000	-1.144302000	-1.395234000
H	3.501671000	-2.345579000	-0.136924000
C	0.723738000	-2.032657000	-2.506641000
H	-0.104166000	-1.629756000	-3.092482000
H	0.760098000	-3.108056000	-2.705408000
H	1.649345000	-1.597212000	-2.882684000
C	-2.018238000	-2.227898000	-1.013351000
H	-2.871165000	-2.042866000	-0.360808000
H	-2.017834000	-3.294216000	-1.257811000
H	-2.186228000	-1.686294000	-1.946995000
C	-1.492625000	-1.733368000	2.106987000
H	-1.250208000	-1.072641000	2.939996000
H	-1.512056000	-2.756481000	2.492599000
H	-2.500215000	-1.490108000	1.770013000
C	1.623449000	-1.142637000	2.461238000
H	2.028348000	-2.087145000	2.836445000
H	0.961390000	-0.737506000	3.226502000
H	2.459113000	-0.450511000	2.346126000
C	2.172974000	2.612298000	-0.014815000
N	1.411842000	1.755793000	-0.053455000
C	3.134789000	3.683784000	0.038226000
H	4.033327000	3.413545000	-0.517698000
H	2.712476000	4.589891000	-0.397715000
H	3.409759000	3.889950000	1.073447000

B2'

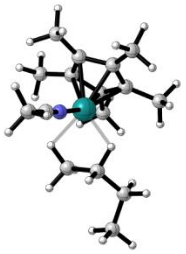

Ru	0.104299000	0.095886000	-0.103610000
H	-1.487759000	0.610681000	0.868160000
H	-2.321009000	2.021508000	0.248738000
C	-2.283698000	0.935181000	0.125298000
C	-3.556866000	0.296135000	0.659973000
H	-3.638923000	0.508994000	1.729515000
H	-3.465267000	-0.793540000	0.569587000
C	-4.799139000	0.780631000	-0.059154000
C	-2.030486000	0.591796000	-1.336826000
H	-2.396176000	-0.403916000	-1.591514000
H	-0.970351000	0.670946000	-1.712996000
H	-2.534533000	1.312318000	-1.984567000
H	-4.800977000	0.491056000	-1.112142000
H	-4.877924000	1.870035000	-0.013091000
H	-5.700520000	0.366448000	0.392450000
C	2.073417000	-0.719890000	-0.084036000
C	1.276979000	-1.431010000	-1.031994000
C	0.180726000	-2.049095000	-0.332355000
C	0.298774000	-1.704181000	1.041910000
C	1.459832000	-0.865616000	1.206334000
C	3.323623000	0.022800000	-0.377266000
H	3.499162000	0.814950000	0.351388000
H	3.297399000	0.477061000	-1.368909000
H	4.186012000	-0.648856000	-0.344272000
C	1.543475000	-1.550266000	-2.485685000
H	0.618043000	-1.605898000	-3.061097000
H	2.112051000	-2.461531000	-2.694971000
H	2.123723000	-0.706826000	-2.860410000
C	-0.850247000	-2.924500000	-0.945005000
H	-1.782577000	-2.911605000	-0.378234000
H	-0.506604000	-3.962106000	-0.983437000
H	-1.077834000	-2.622542000	-1.968938000
C	-0.622918000	-2.111771000	2.130429000
H	-0.713668000	-1.333654000	2.890450000
H	-0.253078000	-3.012540000	2.628736000
H	-1.622319000	-2.332367000	1.752742000
C	1.981362000	-0.335373000	2.489998000
H	2.692439000	-1.034511000	2.940239000
H	1.179973000	-0.171810000	3.211368000
H	2.499387000	0.614240000	2.348604000
C	0.977358000	3.211673000	-0.065896000
N	0.630254000	2.118379000	-0.090578000
C	1.418097000	4.582423000	-0.033988000
H	2.489283000	4.638929000	-0.231464000
H	0.890626000	5.165768000	-0.789727000
H	1.218758000	5.017506000	0.946275000

TS$_{C2\text{-}E2}$

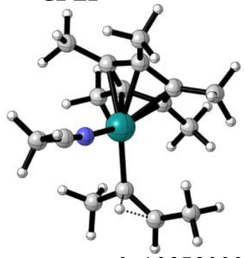

Ru	-0.103730000	0.127194000	0.139764000
C	0.956325000	1.882226000	0.706525000
C	1.538926000	2.846118000	-0.111211000
H	2.187697000	3.601370000	0.345912000
H	0.186995000	2.755334000	0.355439000
C	1.374485000	2.918684000	-1.579459000
C	1.292262000	1.962668000	2.171112000
H	2.103757000	1.257498000	2.367758000
H	0.456744000	1.659125000	2.803275000
H	1.632359000	2.953719000	2.489357000
H	0.572406000	2.259412000	-1.921296000
H	2.305194000	2.574505000	-2.043326000
H	1.210753000	3.938455000	-1.931453000
C	-0.346603000	-1.992652000	0.476498000
C	0.989435000	-1.568042000	0.843001000
C	1.635224000	-1.061662000	-0.318566000
C	0.684274000	-1.124916000	-1.392555000
C	-0.520778000	-1.741325000	-0.903153000
C	-1.323840000	-2.605032000	1.409438000
H	-2.347137000	-2.500299000	1.047032000
H	-1.270913000	-2.144702000	2.397795000
H	-1.125692000	-3.673625000	1.536822000
C	1.573731000	-1.720159000	2.196869000
H	0.868045000	-1.417088000	2.973711000
H	2.483982000	-1.132782000	2.318430000
H	1.831020000	-2.767152000	2.385459000
C	3.042399000	-0.596624000	-0.422832000
H	3.153734000	0.182748000	-1.178957000
H	3.706839000	-1.418106000	-0.704614000
H	3.407021000	-0.193563000	0.523381000
C	0.923501000	-0.732890000	-2.801142000
H	0.024648000	-0.315066000	-3.258416000
H	1.214049000	-1.606003000	-3.394530000
H	1.723701000	0.002455000	-2.889708000
C	-1.729097000	-2.012912000	-1.721206000
H	-2.614584000	-2.147834000	-1.099015000
H	-1.603398000	-2.922396000	-2.315593000
H	-1.930798000	-1.195133000	-2.416652000
C	-3.107421000	1.281047000	0.184847000
N	-2.012191000	0.934852000	0.167109000
C	-4.482387000	1.708098000	0.205812000
H	-4.858327000	1.801918000	-0.814070000
H	-5.095020000	0.977313000	0.735687000
H	-4.576240000	2.673650000	0.704324000

$TS_{C2\text{-}I1}$

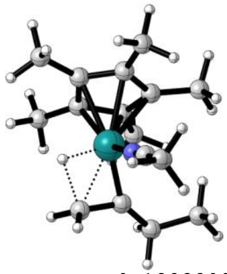

Ru	-0.132339000	0.067894000	0.460522000
C	0.133782000	1.891467000	0.879509000
C	0.596232000	3.176376000	0.344778000
H	1.688387000	3.197111000	0.487170000
H	0.225685000	3.975556000	0.996936000
C	0.236225000	3.442724000	-1.102849000
C	-0.125036000	1.374991000	2.159994000
H	0.675730000	1.315873000	2.897917000
H	-0.471962000	-0.342922000	1.977561000
H	-1.098871000	1.567384000	2.610828000
H	-0.847107000	3.442448000	-1.235250000
H	0.645647000	2.679243000	-1.764189000
H	0.615035000	4.412134000	-1.426003000
C	0.707488000	-2.031751000	0.189089000
C	1.737007000	-1.061347000	0.441282000
C	1.786669000	-0.165925000	-0.685147000
C	0.736066000	-0.512674000	-1.572989000
C	0.070772000	-1.660443000	-1.015016000
C	0.410026000	-3.225464000	1.023307000
H	-0.639335000	-3.514747000	0.959063000
H	0.642126000	-3.052900000	2.074376000
H	1.005844000	-4.080935000	0.692204000
C	2.706847000	-1.098894000	1.566293000
H	2.248478000	-1.490719000	2.475592000
H	3.099508000	-0.106449000	1.793531000
H	3.559675000	-1.740045000	1.325186000
C	2.855603000	0.841281000	-0.908649000
H	2.548613000	1.634029000	-1.590353000
H	3.732011000	0.356173000	-1.347773000
H	3.183819000	1.301695000	0.024492000
C	0.452881000	0.053716000	-2.918630000
H	-0.600668000	0.315044000	-3.038249000
H	0.698769000	-0.670375000	-3.701171000
H	1.039642000	0.951259000	-3.112647000
C	-1.063662000	-2.363898000	-1.664971000
H	-1.669022000	-2.915505000	-0.944826000
H	-0.689280000	-3.083797000	-2.398615000
H	-1.712425000	-1.668554000	-2.200172000
C	-3.338878000	0.117970000	0.194071000
N	-2.196856000	0.129949000	0.286299000
C	-4.774248000	0.106587000	0.077133000
H	-5.073566000	-0.465250000	-0.802086000
H	-5.223237000	-0.346730000	0.961777000
H	-5.149239000	1.125699000	-0.025586000

I1

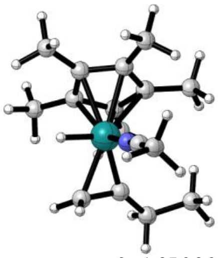

Ru	0.165080000	0.055476000	-0.475233000
C	-0.153680000	1.900778000	-0.853832000
C	-0.770168000	3.133645000	-0.351792000
H	-1.835725000	3.078452000	-0.626230000
H	-0.384523000	3.966534000	-0.950675000
C	-0.604447000	3.411082000	1.127497000
C	0.216346000	1.427504000	-2.108676000
H	-0.518608000	1.389757000	-2.913389000
H	0.616111000	-0.670104000	-1.810963000
H	1.235175000	1.568152000	-2.465102000
H	0.452529000	3.433880000	1.399039000
H	-1.082747000	2.647205000	1.738385000
H	-1.039788000	4.374956000	1.390354000
C	-0.050386000	-1.561045000	1.134024000
C	-0.511916000	-2.072449000	-0.104780000
C	-1.594733000	-1.233968000	-0.540048000
C	-1.834166000	-0.262057000	0.492607000
C	-0.872915000	-0.459330000	1.524480000
C	1.058365000	-2.111040000	1.953412000
H	1.597090000	-1.320319000	2.478528000
H	1.772008000	-2.672600000	1.349732000
H	0.664476000	-2.793244000	2.712597000
C	-0.020440000	-3.299761000	-0.785032000
H	1.053531000	-3.435098000	-0.652207000
H	-0.221102000	-3.277558000	-1.855853000
H	-0.514900000	-4.184403000	-0.373812000
C	-2.444620000	-1.452339000	-1.738961000
H	-2.848874000	-0.514157000	-2.121983000
H	-3.292738000	-2.101073000	-1.500994000
H	-1.883520000	-1.923415000	-2.546485000
C	-3.006791000	0.650072000	0.518753000
H	-2.857598000	1.508599000	1.173049000
H	-3.884471000	0.109801000	0.885089000
H	-3.255865000	1.018401000	-0.477785000
C	-0.810111000	0.198308000	2.857741000
H	0.165404000	0.648595000	3.052835000
H	-0.992650000	-0.534672000	3.649000000
H	-1.563781000	0.977838000	2.962579000
C	3.351092000	0.288224000	-0.134749000
N	2.212528000	0.235810000	-0.248821000
C	4.782349000	0.357144000	0.010437000
H	5.270099000	0.117181000	-0.935135000
H	5.080902000	1.360377000	0.317081000
H	5.115665000	-0.353322000	0.767869000

TS_{I1-I2}

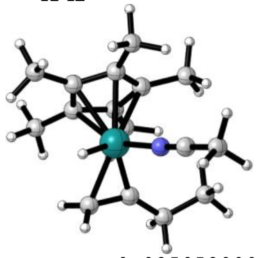

Ru	0.025952000	0.008788000	-0.562389000
C	0.497621000	1.810181000	-0.900924000
C	1.144725000	2.966649000	-0.291537000
H	0.365200000	3.737644000	-0.186316000
H	1.806532000	3.391196000	-1.060431000
C	1.875778000	2.728860000	1.010344000
C	-0.117583000	1.489203000	-2.107706000
H	-1.142860000	1.800474000	-2.307356000
H	0.069493000	-0.719911000	-1.986774000
H	0.489924000	1.418250000	-3.007662000
H	2.658573000	1.978837000	0.886375000
H	1.196804000	2.370577000	1.784890000
H	2.340886000	3.646436000	1.369342000
C	-0.689407000	-1.614547000	0.821691000
C	-1.700548000	-1.419675000	-0.147267000
C	-2.129618000	-0.057824000	-0.049522000
C	-1.464054000	0.540814000	1.080995000
C	-0.551946000	-0.401378000	1.602943000
C	0.035991000	-2.882247000	1.096707000
H	1.028148000	-2.694813000	1.509802000
H	0.152596000	-3.486486000	0.196071000
H	-0.509145000	-3.484114000	1.829545000
C	-2.254615000	-2.437178000	-1.076055000
H	-1.536553000	-3.229974000	-1.284673000
H	-2.551859000	-1.996143000	-2.027497000
H	-3.142048000	-2.901619000	-0.636518000
C	-3.235158000	0.559521000	-0.825332000
H	-3.128418000	1.643495000	-0.895840000
H	-4.198151000	0.363214000	-0.344832000
H	-3.286676000	0.160728000	-1.839379000
C	-1.778795000	1.891540000	1.614622000
H	-0.996491000	2.270941000	2.271625000
H	-2.705543000	1.857459000	2.194467000
H	-1.933308000	2.619415000	0.815315000
C	0.303914000	-0.273509000	2.811570000
H	1.343577000	-0.538440000	2.605041000
H	-0.047633000	-0.940183000	3.604321000
H	0.294354000	0.740645000	3.210274000
C	3.047597000	-1.068502000	-0.449593000
N	1.987111000	-0.636860000	-0.494707000
C	4.381599000	-1.610891000	-0.407906000
H	4.693953000	-1.916788000	-1.407291000
H	5.082673000	-0.864787000	-0.031989000
H	4.411308000	-2.480820000	0.249376000

I2

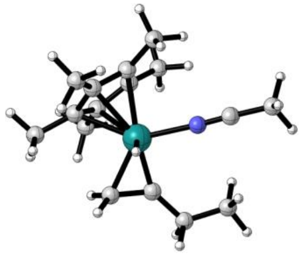

Ru	0.007326000	0.311156000	-0.308446000
C	1.007589000	1.916779000	-0.284630000
C	2.315882000	2.571977000	-0.200384000
H	2.166074000	3.387964000	0.522422000
H	2.466511000	3.104540000	-1.150296000
C	3.536832000	1.762392000	0.170714000
C	-0.250295000	2.365497000	-0.701337000
H	-0.906463000	2.848652000	0.022080000
H	0.219590000	0.222006000	-1.892747000
H	-0.398272000	2.713952000	-1.722413000
H	3.809368000	1.070015000	-0.626592000
H	3.371026000	1.192931000	1.087142000
H	4.388066000	2.422740000	0.335800000
C	-1.848124000	-0.996816000	-0.754174000
C	-2.223901000	0.246026000	-0.161678000
C	-1.687658000	0.288574000	1.179988000
C	-0.920449000	-0.881250000	1.372772000
C	-1.019984000	-1.674118000	0.161729000
C	-2.296220000	-1.489967000	-2.080414000
H	-1.593998000	-2.207313000	-2.504816000
H	-2.415573000	-0.674512000	-2.793888000
H	-3.264904000	-1.990069000	-1.987880000
C	-3.212179000	1.188313000	-0.742446000
H	-3.025946000	1.365656000	-1.802646000
H	-3.216078000	2.150925000	-0.232796000
H	-4.217961000	0.766566000	-0.653566000
C	-1.972492000	1.350037000	2.175453000
H	-1.306086000	1.288585000	3.034974000
H	-2.999433000	1.261101000	2.541018000
H	-1.879767000	2.352565000	1.750876000
C	-0.229457000	-1.308477000	2.616151000
H	0.728319000	-1.786393000	2.404301000
H	-0.837672000	-2.033686000	3.164651000
H	-0.038459000	-0.466340000	3.280655000
C	-0.448416000	-3.030204000	-0.032694000
H	-0.093963000	-3.176420000	-1.054555000
H	-1.210703000	-3.790091000	0.160442000
H	0.379889000	-3.224817000	0.648632000
C	2.717505000	-1.422621000	-0.241256000
N	1.774237000	-0.774511000	-0.243837000
C	3.929840000	-2.198849000	-0.236715000
H	4.766759000	-1.564531000	0.062117000
H	3.848659000	-3.025622000	0.469826000
H	4.128163000	-2.601472000	-1.230722000

TS$_{I2\text{-}D4}$

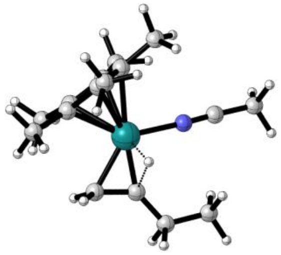

Ru	-0.013459000	-0.365325000	-0.094322000
C	-1.040990000	-1.964219000	-0.012277000
C	-2.353084000	-2.617222000	-0.099809000
H	-2.417650000	-3.238757000	0.804445000
H	-2.284832000	-3.352555000	-0.913630000
C	-3.593780000	-1.769819000	-0.255475000
C	0.266334000	-2.451487000	0.116036000
H	0.582470000	-2.827557000	1.089276000
H	-0.639264000	-1.011593000	-1.477348000
H	0.773531000	-2.914744000	-0.728270000
H	-3.536060000	-1.147319000	-1.150716000
H	-3.744651000	-1.125369000	0.611642000
H	-4.471811000	-2.408306000	-0.351349000
C	1.740277000	0.712672000	-1.111735000
C	2.202413000	-0.304845000	-0.225185000
C	1.813000000	0.059125000	1.120853000
C	1.060187000	1.248220000	1.042363000
C	1.015042000	1.649902000	-0.351760000
C	1.998197000	0.769215000	-2.571659000
H	1.225362000	1.329915000	-3.097479000
H	2.047673000	-0.227669000	-3.010294000
H	2.955202000	1.261610000	-2.768086000
C	3.137317000	-1.392302000	-0.602080000
H	2.870176000	-1.842414000	-1.559773000
H	3.173892000	-2.183316000	0.146217000
H	4.149378000	-0.989629000	-0.705840000
C	2.200700000	-0.670450000	2.350751000
H	1.545801000	-0.430465000	3.188027000
H	3.221967000	-0.406668000	2.640979000
H	2.181546000	-1.752324000	2.207501000
C	0.502670000	2.026201000	2.176972000
H	-0.462051000	2.471201000	1.929778000
H	1.176048000	2.844518000	2.447952000
H	0.364528000	1.404593000	3.061255000
C	0.403963000	2.896720000	-0.876914000
H	-0.095285000	2.730414000	-1.833306000
H	1.175658000	3.654275000	-1.039366000
H	-0.320843000	3.320179000	-0.181933000
C	-2.714703000	1.397018000	0.023780000
N	-1.774976000	0.743935000	-0.004477000
C	-3.924607000	2.176375000	0.056481000
H	-4.781461000	1.514448000	-0.085180000
H	-4.028276000	2.683733000	1.016213000
H	-3.921646000	2.919907000	-0.741265000

10. References

(1) Zhao, Y.; Truhlar, D. G. *Theor. Chem. Acc.* **2008**, *120*, 215-241.

(2) a) Schäfer, A.; Horn, H.; Ahlrichs, R. *J. Chem. Phys.* **1992**, *97*, 2571-2577. b) Weigend, F.; Ahlrichs, R. *Phys. Chem. Chem. Phys.* **2005**, *7*, 3297-3305. c) Weigend, F. *Phys. Chem. Chem. Phys.* **2006**, *8*, 1057-1065.

(3) Andrae, D.; Häussermann, U.; Dolg, M.; Stoll, H.; *Theor. Chim. Acta* **1990**, *77*, 123-141.

(4) Marenich, A. V.; Cramer, C. J.; Truhlar, D. G. *J. Phys. Chem. B* **2009**, *113*, 6378-6396.

(5) Gaussian 09, Revision D.01, M. J. Frisch, G. W. Trucks, H. B. Schlegel, G. E. Scuseria, M. A. Robb, J. R. Cheeseman, G. Scalmani, V. Barone, B. Mennucci, G. A. Petersson, H. Nakatsuji, M. Caricato, X. Li, H. P. Hratchian, A. F. Izmaylov, J. Bloino, G. Zheng, J. L. Sonnenberg, M. Hada, M. Ehara, K. Toyota, R. Fukuda, J. Hasegawa, M. Ishida, T. Nakajima, Y. Honda, O. Kitao, H. Nakai, T. Vreven, J. A. Montgomery, Jr., J. E. Peralta, F. Ogliaro, M. Bearpark, J. J. Heyd, E. Brothers, K. N. Kudin, V. N. Staroverov, T. Keith, R. Kobayashi, J. Normand, K. Raghavachari, A. Rendell, J. C. Burant, S. S. Iyengar, J. Tomasi, M. Cossi, N. Rega, J. M. Millam, M. Klene, J. E. Knox, J. B. Cross, V. Bakken, C. Adamo, J. Jaramillo, R. Gomperts, R. E. Stratmann, O. Yazyev, A. J. Austin, R. Cammi, C. Pomelli, J. W. Ochterski, R. L. Martin, K. Morokuma, V. G. Zakrzewski, G. A. Voth, P. Salvador, J. J. Dannenberg, S. Dapprich, A. D. Daniels, O. Farkas, J. B. Foresman, J. V. Ortiz, J. Cioslowski, and D. J. Fox, Gaussian, Inc., Wallingford CT, 2013.

(6) CYLview, 1.0b; Legault, C. Y., Université de Sherbrooke, **2009**(http://www.cylview.org)

CRYSTALLOGRAPHIC SECTION

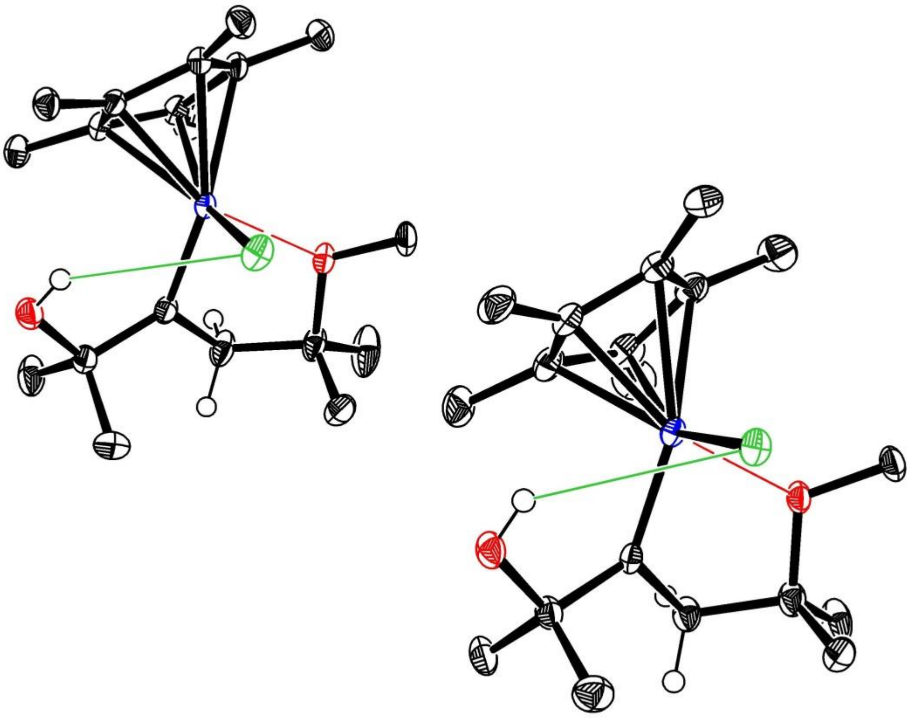

Figure S-1. Structure of the ruthenium carbene **9b** (R = Me) in the solid state, showing the two independent molecules in the unit cell; hydrogen atoms are omitted for clarity, except those of the –OH group and the -CH_2-group flanking the carbene center; color code: Ru = blue, O = red, Cl = green

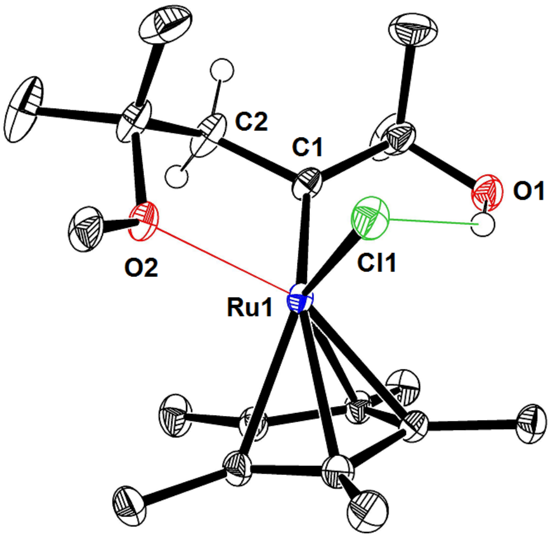

Figure S-2. Different projection showing one of the two independent molecules of ruthenium carbene **9b** (R = Me) present in the unit cell; hydrogen atoms are omitted for clairity, except the one on O1 involved in hydrogen bonding with Cl1 and the geminal H-atoms at C2 flanking the carbene center C1; color code: Ru = blue, O = red, Cl = green

X-ray Crystal Structure Analysis of Complex 9b: $C_{19} H_{33} Cl O_2 Ru$, M_r = 429.97 g · mol^{-1}, orange block, crystal size 0.18 x 0.10 x 0.02 mm, triclinic, space group $P\bar{1}$, a = 9.1699(13) Å, b = 14.0446(13) Å, c = 15.7493(16) Å, α = 86.283(10)°, β = 89.332(10)°, γ = 83.746(10)°, V = 2012.0(4) Å^3, T = 100 K, Z = 4, D_{calc} = 1.419 g·cm^3, λ = 0.71073 Å, $\mu(Mo\text{-}K_\alpha)$ = 0.919 mm^{-1}, Gaussian absorption correction (T_{min} = 0.85, T_{max} = 0.98), Bruker-AXS Smart APEX-II diffractometer, 2.799 < θ < 32.032°, 39856 measured reflections, 13943 independent reflections, 10867 reflections with $I > 2\sigma(I)$, Structure solved by direct methods and refined by full-matrix least-squares against F^2 to R_1 = 0.038 [$I > 2\sigma(I)$], wR_2 = 0.099, 437 parameters, H atoms riding, S = 1.019, residual electron density 1.5 / -1.5 e Å^{-3}.

CCDC 1406683 contains the supporting crystallographic data for this paper. These data can be obtained free of charge from the Cambridge Crystallographic Data Centre via www.ccdc.cam.ac.uk/data_request/cif.

NMR INVESTIGATIONS

General. The CD_2Cl_2 used in these experiments was dried by distillation over $CaCO_3$ and stored in a Schlenk-flask in a glovebox. Unless stated otherwise, all commercially available compounds were used as received and stored under argon. [Cp*Ru(CH_3CN)$_3$]PF_6 was prepared according to a literature procedure.[1] [Cp*Ru(cod)Cl] was purchased from Strem.

Sample Preparation. All samples were prepared in a glovebox. The substrate (0.1 mmol) and the catalyst (5.5 mol%) were dissolved in 0.4 mL CD_2Cl_2 in a 2 mL GC vial. After transferring the material into the pressure NMR-tube (5 mm medium wall precision pressure/vacuum valve NMR sample tube, Wilmad) via syringe, the tube was connected to the *p*-H_2-storage container or directly to the generator. The tubing was flushed with *p*-H_2 to ensure that no other gases were present. Then the Swagelok® connection to the NMR tube was tightened and the pressure valve opened to fill the tube with hydrogen. After closing the valve, the tube was shaken and directly transferred into the NMR magnet.

NMR Measurements. All spectra were acquired on an Bruker Ascend AVIII 500 MHz NMR spectrometer (11.7 Tesla) equipped with an Bruker 5mm BBFO^{plus} 500 MHz SmartProbe™ (PA BBO 500S1 BBF-H-D-05 Z Plus) or Bruker 5mm TBI Probe (PH TBI 500S1 H/C-BB-D-05 Z) at 298 K unless otherwise mentioned.

The acquired ^{1}H NMR spectra were referenced to the residual solvent signal (δ_{CHDCl2} = 5.32 ppm)[2]. The ^{13}C NMR spectra were referenced with the Ξ-scale.[3,4]

For the OPSY-EXSY spectrum, a mixing time of 300 ms was used.

NMR data was processed with Bruker's Topspin 3.2. For the simulation of the NMR spectra, the DAISY module of Topspin was used. The NMR assignment of the carbenes **9** was performed with MestreNova 9.1.

***para*-Hydrogen Generation**. The *p*-H_2 enrichment above the thermal equilibrium of 25% was achieved in two different ways.

Initially, the *p*-H_2 was enriched to 50% using the "U-shaped tube" method (Figure S-3).[5] The tube was filled with a mixture (3:1) of activated charcoal (Norit PK1-3, Sigma Aldrich) and iron(III) oxide (99%, meshed powder, Alfa Aesar). The filled tube was evacuated and heated with a heat gun (150 °C) to remove any residual water and oxygen from the catalyst. This tube was used several times before the catalyst had to be reactivated. To enrich the *p*-H_2, the tube was loaded with 20 bar of hydrogen gas (99.995%, dry) and placed in a Dewar flask filled with liquid nitrogen (77 K). After an equilibration time of 1h, the enriched hydrogen gas was transferred to an evacuated storage bottle or directly transferred to the NMR tube.

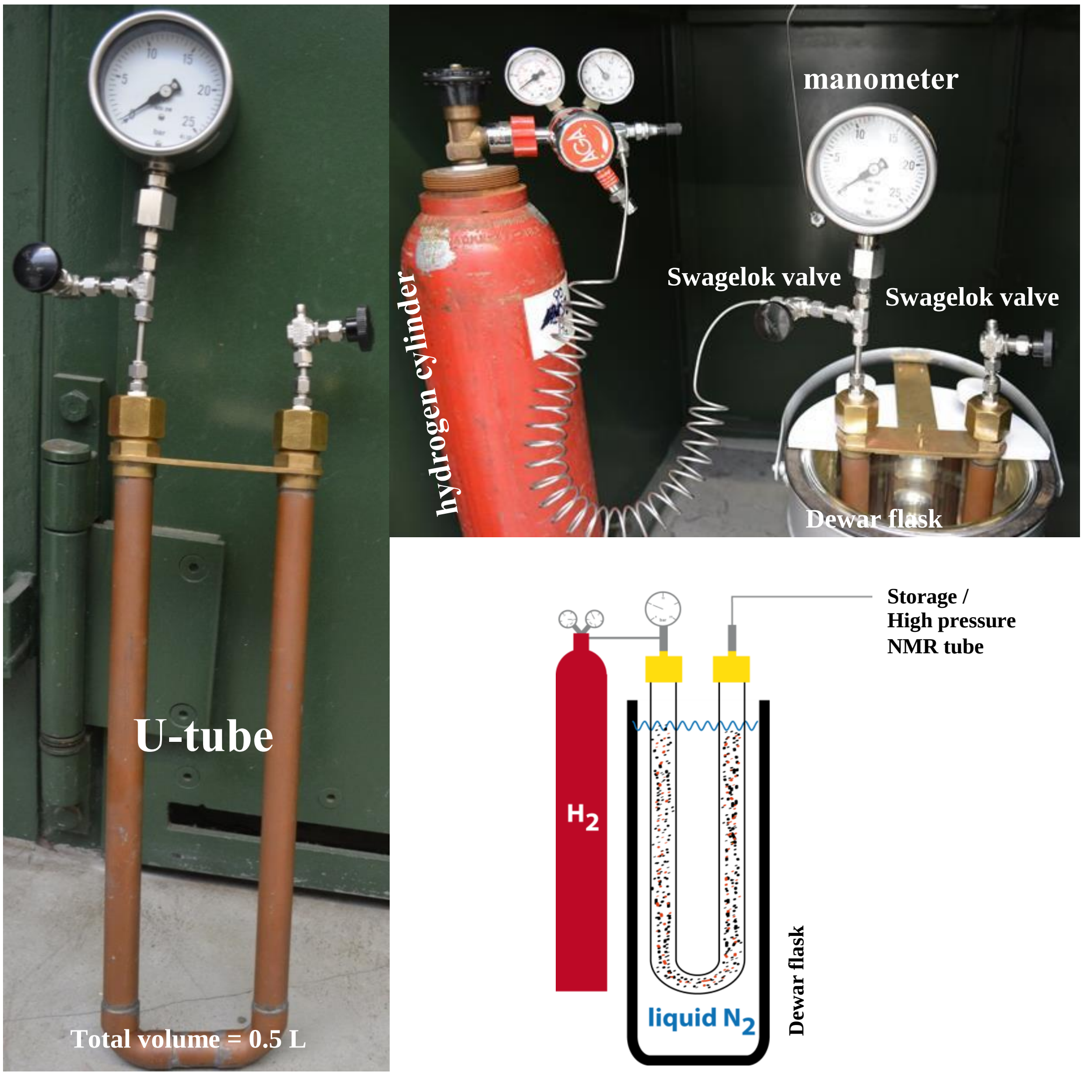

Figure S-3. Apparatus for the *p*-H_2 enrichment to 50%

An enrichment of approx. 92 % was achieved with the commercially available *Parahydrogen pH2 Generator* from *Bruker BioSpin GmbH.*

2D-EXSY with OPSY Filter. A 2D-EXSY (Exchange SpectrosocopY) experiment was adapted to *p*–H_2 induced polarization with an OPSY-d-filter (Figure S-4) to follow chemical exchanges involving the hydrogenated species during the reaction. Typically, experiments were recorded with 512 increments and 2 scans (8k points) per increments. Mixing times of τ_{mix} = 2τ = 300 ms were used. Short repetition times of 1.1 s (τ_{mix} + aq) allowed for an overall experimental time of 20 min (relaxation delays were unnecessary).

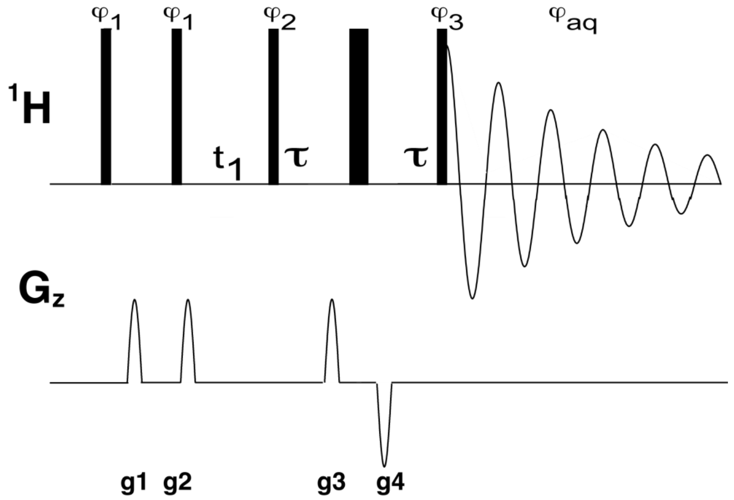

Figure S-4. NOESY/EXSY with OPSY-*d*-Filter (OPSY-*d*-EXSY): *Black thin bars* represent 90° *pulses* and thick *bars* represent 180° *pulses; pulses are applied with x-phase unless the phase is indicated above the bar.* Phase cycle: φ_1 = [x,-x], φ_2 = [(x)$_8$ (-x)$_8$], φ_3 = [x,x,-x,-x,y,y,-y,-y], φ_{aq} =[x,-x,-x,x,y,-y,-y,y, -x,x,x,-x,-y,y,y,-y]. Half-sine 1ms gradients were used with gradient ratio g1:g2:g3:g4 = 10:20:4:-4, and were each followed by a 0.2 ms recovery delay. The chemical exchange mixing time is represented by 2 τ.

NMR Assignments of the Stable Carbenes. Standard ^{1}H,^{1}H-NOESY (t_{mix} = 1s), ^{1}H,^{13}C-HSQC and ^{1}H,^{13}C-HMBC experiments were used for the characterization of the stable carbenes.

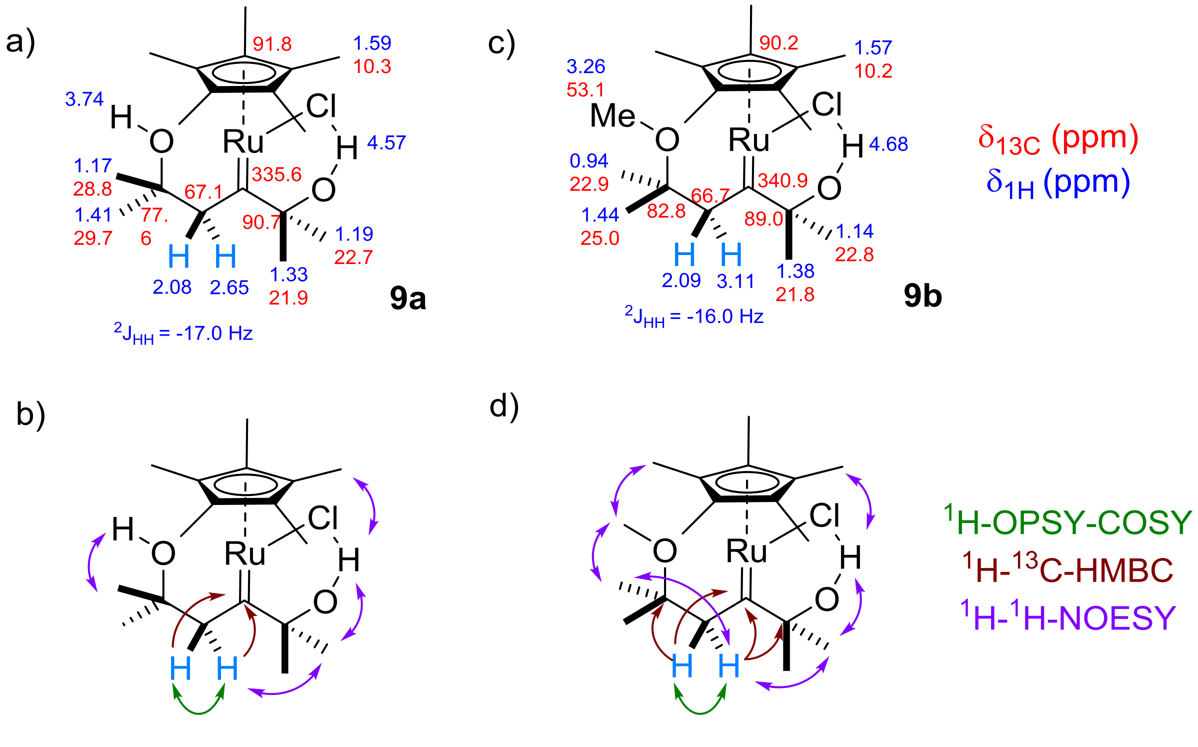

Figure S-5. NMR assignments of **9a** (*a*) and **9b** (*c*) and selected connections observed in the 2D NMR spectra (*b* and *d*)

Sign of the J_{HH} Couplings. The shape of the PASADENA antiphase signal gives information about the sign of the coupling constant. In the case of a positive coupling the first signal of the doublet is positive, whereas the second one is negative. This can be seen for the vicinal coupling of the olefinic protons of **5a** (**Fehler! Verweisquelle konnte nicht gefunden werden.**, left). If the coupling is negative, the sign of the antiphase signals is inverted. This can be nicely seen in the case the hyperpolarized protons of **7a** and **6a** proving that the coupling is indeed negative (Figure S1, middle & left). Negative couplings are normally observed for geminal proton-proton couplings.

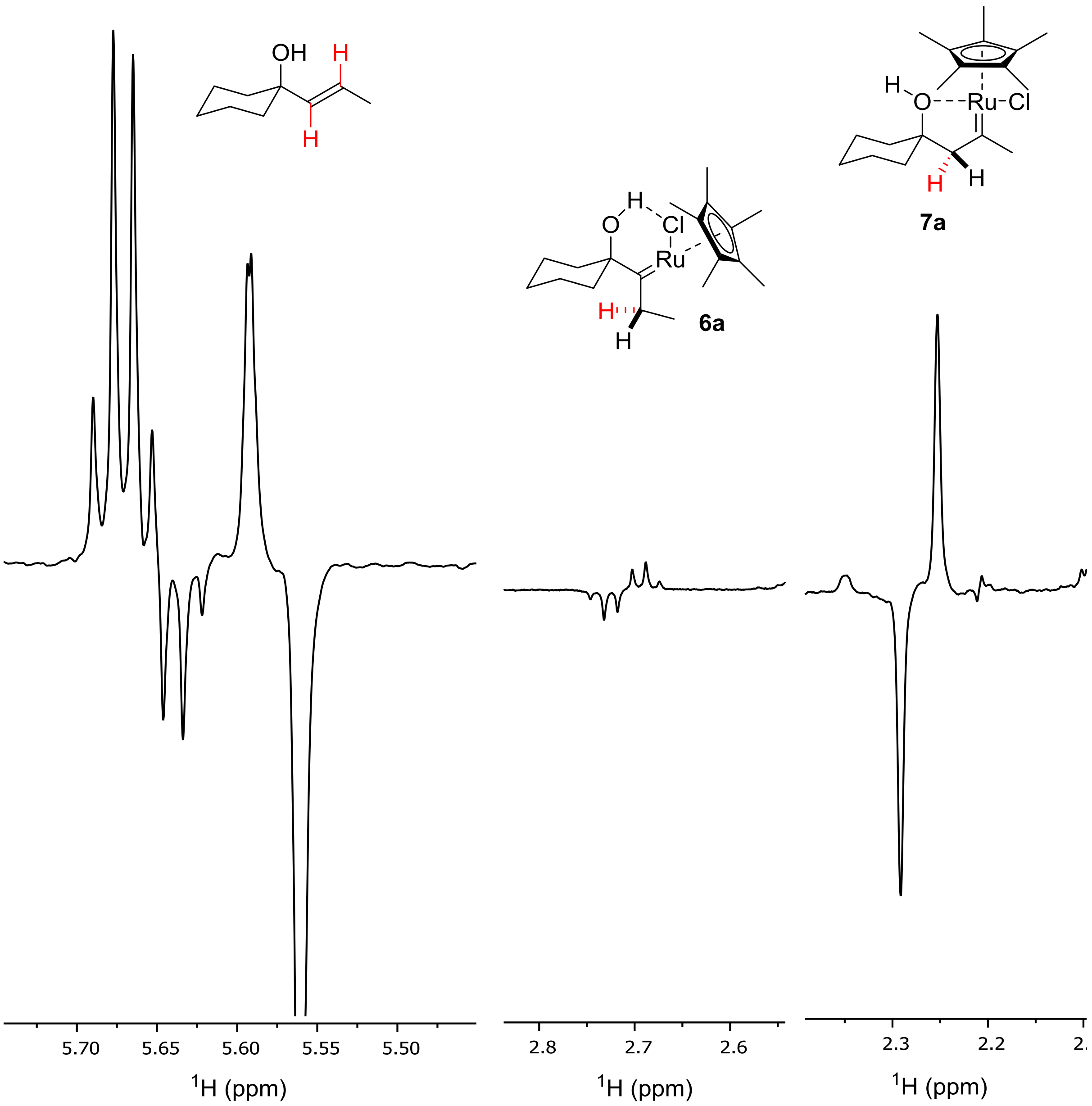

Figure S-6: Hyperpolarized antiphase signals of **5a**, **6a** and **7a**

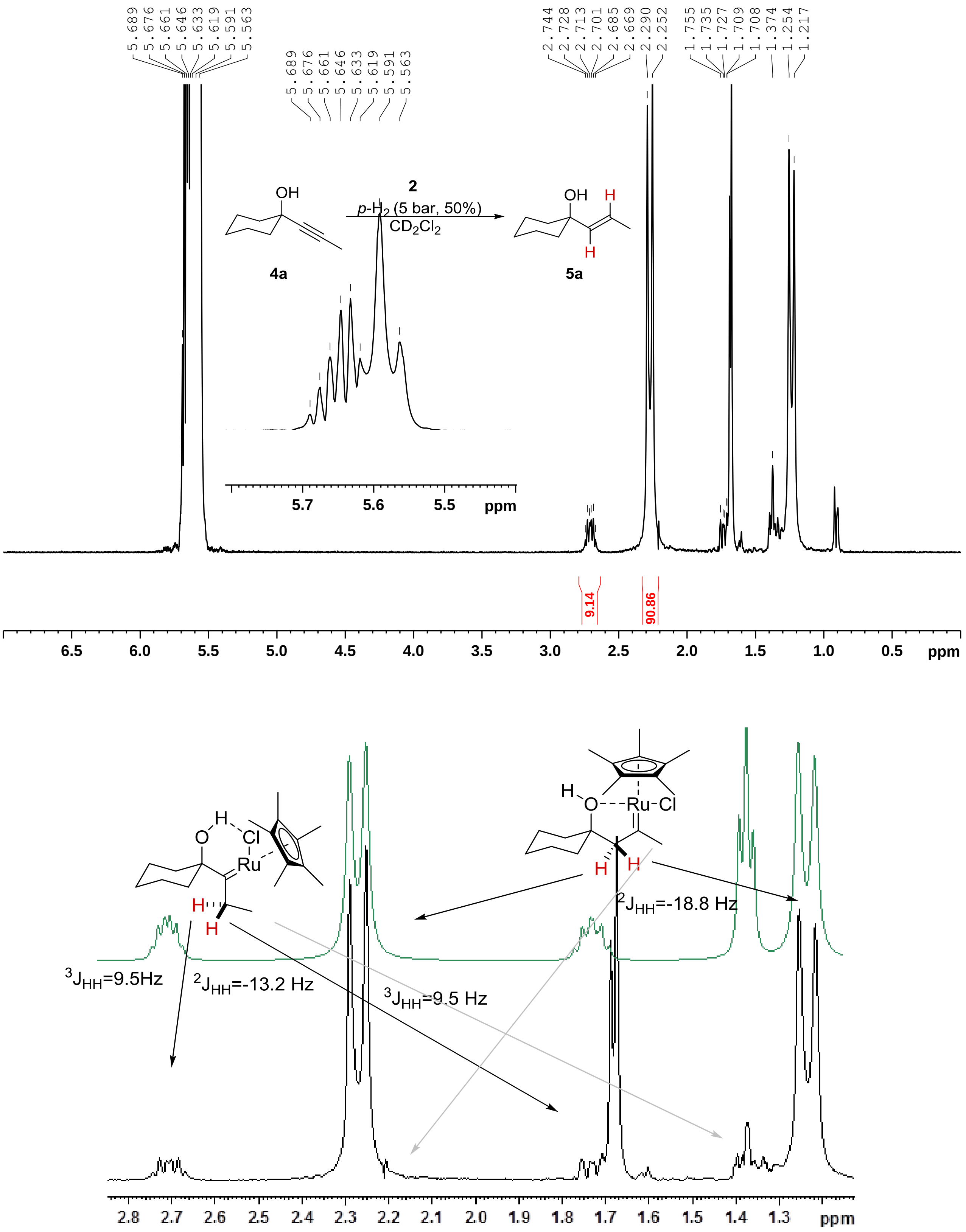

Figure S-7: Top: OPSY-*d*-spectrum during the reaction **4a** in the presence of *p*-H_2 and **2**; Bottom: Comparison of the acquired OPSY-Spectrum (black)and the simulated spectrum (green)

The ^{1}H-OPSY-COSY spectrum contains various structure informations about the carbene intermediates **6a** and **7b**. On the one hand it clearly shows the cross peaks between the geminal protons (**H2α↔H2β**, **H1α↔H1β**). On the other hand asymmetrical cross peaks to the CH_3-groups (H3) can be seen. The asymmetrical cross peaks are explained by the different polarization of the methyl and the methylene protons. The hyperpolarized geminal protons generate this cross peak (H1/2→H3), whereas the non-hyperpolarized methyl group may generate a cross peak (H3→H2/1), but the intensity is lower than the noise level and so not visible.

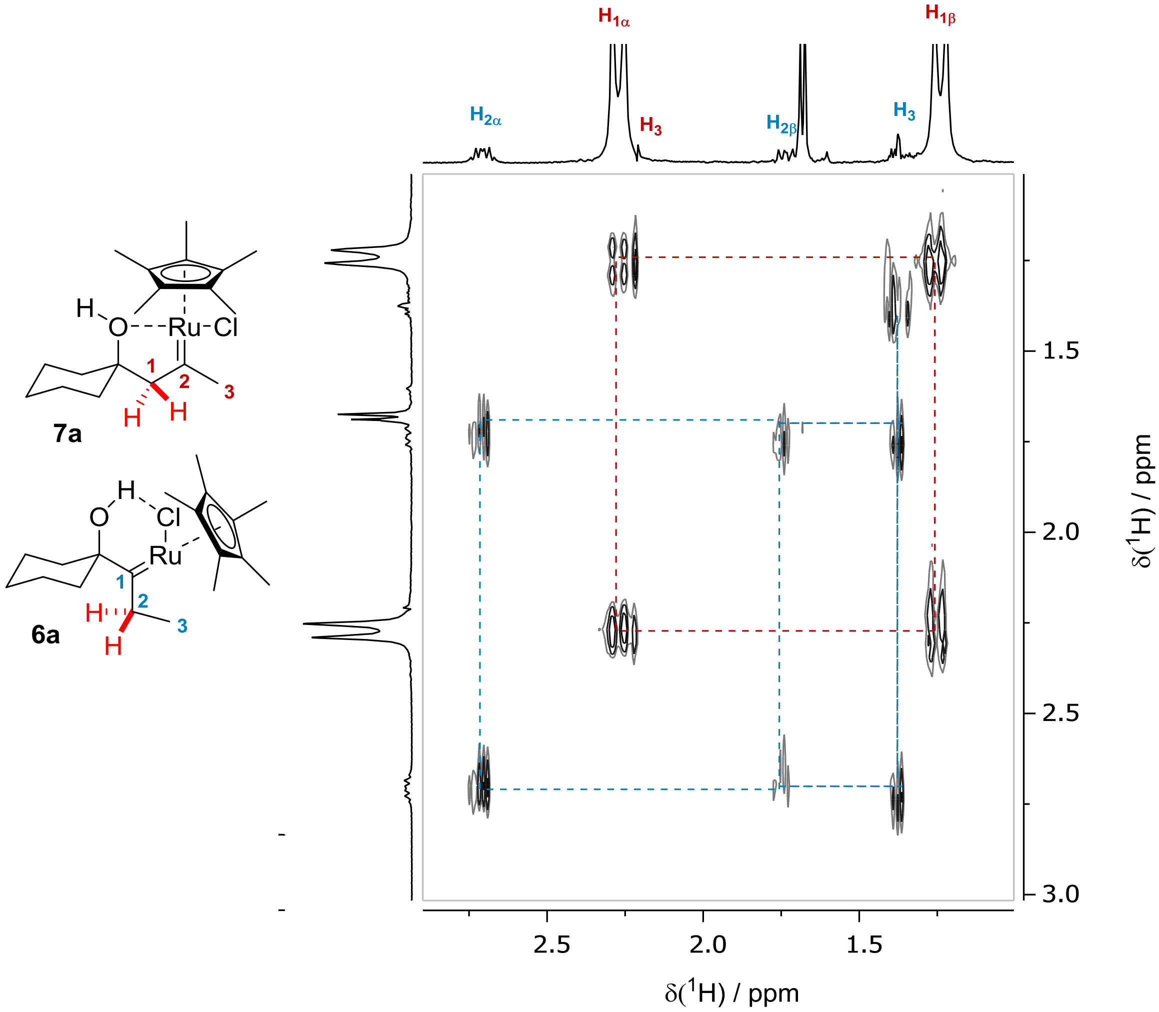

Figure S-8. Aliphatic region of the ^{1}H-OPSY-COSY spectrum confirming the coupling between the observed signals and the coupling to neighbored CH_3-groups

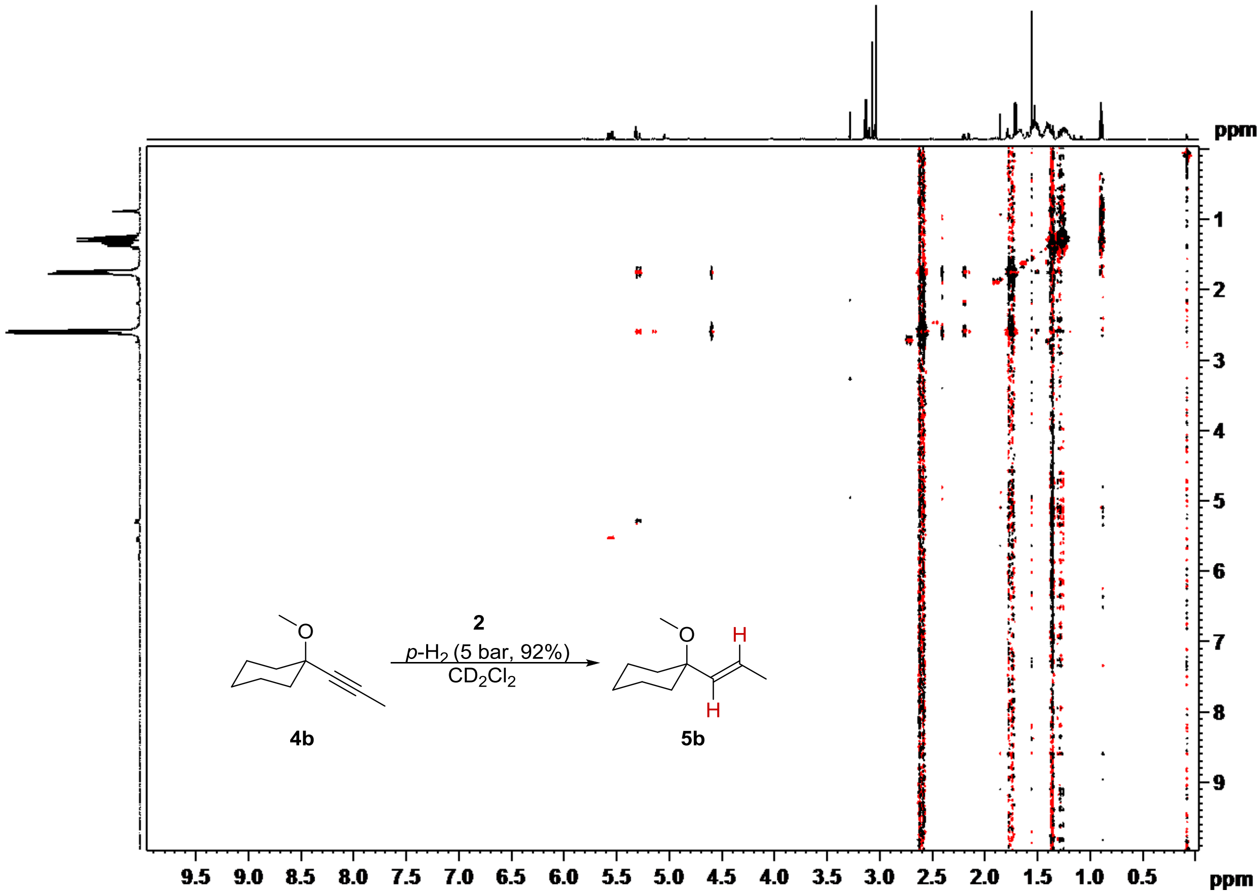

Figure S-9. OPSY-EXSY spectrum of **4b** during the hydrogenation with **2**

Exchange Spectroscopy. The analysis of the OPSY-EXSY spectrum of the reaction **4b** to **5b** finds exchange correlations from the hyperpolarized hydrogens of the carbene **6c** to a number of products and by-products, namely **5b**, **10**, **11** and free H_2, as shown in Figure 4 of the main text of the publication. These results are in excellent accord with the pathways 1 and 2 as proposed in Scheme 3. These observed exchange correlations are depicted by red arrows in Scheme S1. Interestingly, pathway 1 correctly predicts that only one of the olefinic hydrogens originates from the carbene 6c.

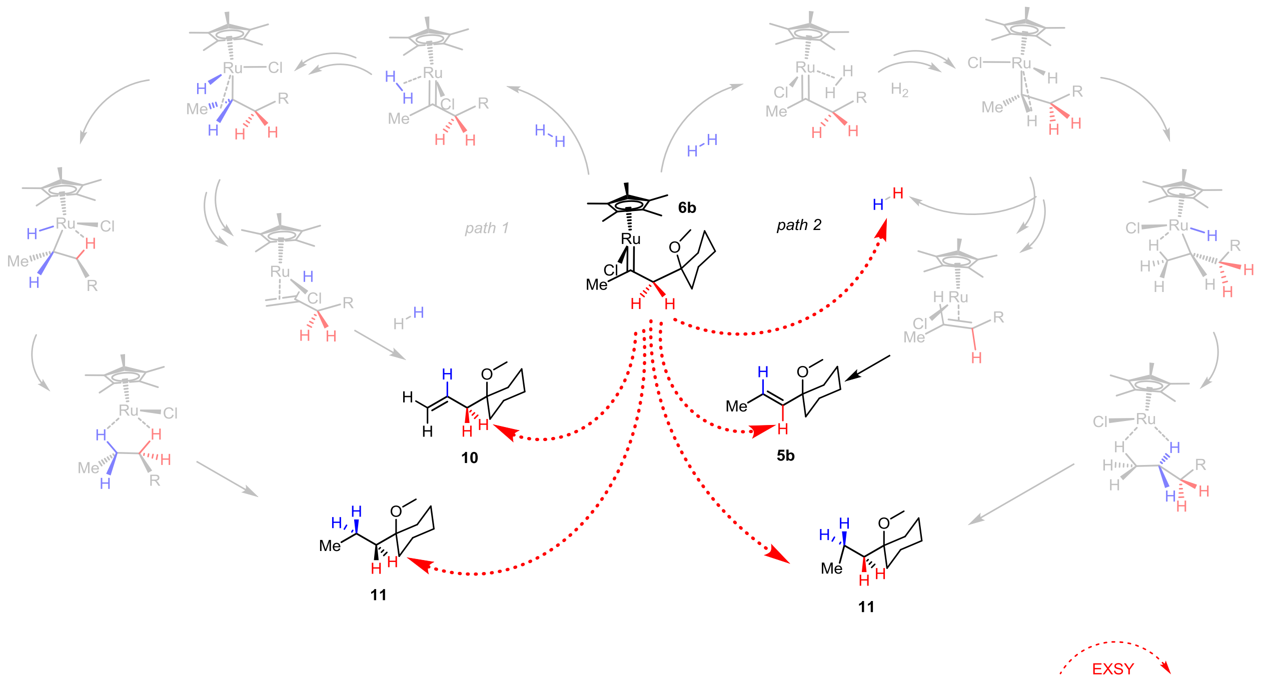

Scheme S1: Comparison of the exchange correlations (red arrows) extracted from the OPSY-EXSY from the carbene 6b. Greyed species are not observed by NMR. Hydrogens are colored to help follow their fate.

References

[1] M. D. Mbaye, B. Demerseman, J.-L. Renaud, L. Toupet, C. Bruneau, *Adv. Synth. Catal.* **2004**, *346*, 835–841.

[2] G. R. Fulmer, A. J. M. Miller, N. H. Sherden, H. E. Gottlieb, A. Nudelman, B. M. Stoltz, J. E. Bercaw, K. I. Goldberg, *Organometallics* **2010**, *29*, 2176–2179.

[3] R. K. Harris, E. D. Becker, S. M. Cabral de Menezes, P. Granger, R. E. Hoffman, K. W. Zilm, *Pure Appl. Chem.* **2008**, *80*, 59–84.

[4] M. Findeisen, S. Berger, *50 and More Essential NMR Experiments: A Detailed Guide*, Wiley-VCH, **2013**.

[5] J. Bargon, J. Kandels, K. Woelk, *Zeitschrift für Phys. Chemie* **1993**, *180*, 65–93.
